# Statescope: an integrative deconvolution framework for discovering cell states in tumors

**DOI:** 10.1038/s41467-026-74997-8

**Published:** 2026-07-06

**Authors:** Jurriaan Janssen, Mischa F. B. Steketee, Aryamaan Bose, Saskia van Asten, Paul P. Eijk, Frederike Dijk, Arantza Farina Sarasqueta, Febe van Maldegem, David P. Noske, Idris Bahce, Jan Koster, Juan J. Garcia Vallejo, Richard Schoonhoven, Mark A. van de Wiel, Teodora Radonic, Bauke Ylstra, Yongsoo Kim

**Affiliations:** 1 Amsterdam UMC, Vrije Universiteit Amsterdam, Department of Pathology, Cancer Center Amsterdam, Amsterdam, The Netherlands; 2 Amsterdam UMC, Vrije Universiteit Amsterdam, Department of Molecular Cell Biology and Immunology, Amsterdam, The Netherlands; 3 Amsterdam UMC, Vrije Universiteit Amsterdam, Department of Neurosurgery, Amsterdam, The Netherlands; 4 Amsterdam UMC, Vrije Universiteit Amsterdam, Department of Pulmonary Medicine, Amsterdam, The Netherlands; 5 Amsterdam UMC, Vrije Universiteit Amsterdam, Laboratory of Experimental Oncology and Radiobiology, Amsterdam, The Netherlands; 6 Amsterdam UMC, Vrije Universiteit Amsterdam, Department of Epidemiology & Data Science, Amsterdam, The Netherlands

**Keywords:** Statistical methods, Cancer microenvironment, Machine learning, Cancer genomics, Tumour heterogeneity

## Abstract

Accurate deconvolution of cell states from bulk tumor RNA-seq is hindered by heterogeneous malignant cells specifically in cancer applications. We present Statescope, a Bayesian framework that incorporates DNA-derived malignant cell purity to overcome this heterogeneity and explicitly models inter-sample variation to accurately identify cell states. Comprehensive benchmarking shows Statescope outperforms existing methods in both cell fraction and state estimation, and is unique in its ability to identify states entirely absent from single-cell references. In real-data applications, Statescope successfully recapitulates established cell states, including multiple states in neutrophils, a cell type often missed by single-cell methods in lung cancer. Critically, in the POPLAR/OAK clinical trials, Statescope identifies a combinatorial signature of effector CD8 + T cells and conventional dendritic cell states that together predict a striking survival benefit from immunotherapy. Collectively, Statescope transforms deconvolution into a versatile discovery platform, enabling deeper biological and clinical insights from widely available bulk multi-omics data.

## Abstract (web summary)

Accurate deconvolution of cell states from bulk tumor RNA-seq can be hindered by heterogeneous malignant cells. Here, the authors present Statescope, a Bayesian framework that incorporates DNA-derived tumor cell purity to overcome heterogeneity, thus identifying cell states and proportions more accurately.

## Introduction

Solid tumors are composed of malignant cells and non-malignant fibroblasts, endothelial, immune, and other microenvironmental components. Accurate identification of distinct cell types and their biological activity is critical and may serve as biomarkers, particularly in immune-oncology. Single-cell RNA sequencing (scRNA-seq) has enabled the characterization of both cell type fractions and gene expression profiles within the tumor microenvironment. However, its technical limitations and high costs make scRNA-seq impractical for studying large patient cohorts and for clinical applications^1,2^. One notable technical limitation is the under-representation of certain cell types, such as neutrophils, which are often depleted during cell sorting due to their low RNA content and sensitivity to enzymatic digestion during tissue dissociation^3^.

An alternative approach to determine cell types and their cellular states is bulk RNA sequencing (RNA-seq) combined with deconvolution algorithms, which have emerged as a cost-effective approach devoid of the aforementioned cost restrictions and technical artifacts. Common deconvolution algorithms, including CIBERSORTx^4^, BayesPrism^5^, BLADE^6^, and TAPE^7^, allow for the estimation of cell-type fractions and cell-type-specific gene expression profiles from RNA-seq data with reference signatures derived from scRNA-seq. A pioneering example is CIBERSORTx, a regression-based deconvolution algorithm that first estimates cell fractions, followed by the inference of cell type-specific gene expression profiles^4^. TAPE was the first deep learning-based method capable of simultaneously estimating both cell fractions and cell type-specific gene expression profiles^7^. Similarly, Bayesian deconvolution approaches such as BLADE^6^ and BayesPrism^5^ provide a unified framework for inferring both in parallel, requiring fewer parameters compared to the deep learning-based model in TAPE. The key distinction between BLADE and BayesPrism lies in their modeling of gene expression variability: BLADE assumes a log-normal distribution and explicitly accounts for the heterogeneity observed within each cell type, whereas BayesPrism employs a multinomial model that does not capture this heterogeneity. Despite the advancements in RNA deconvolution methods, integration of extra information to improve its performance remains underexplored. For instance, DNA-based data is often available, or can be produced, alongside RNA-seq in cancer studies and has been shown to improve malignant cell fraction estimation, as demonstrated by Cao et al.^8^. Furthermore, while many studies have benchmarked the accuracy of deconvolution algorithms in estimating cell fractions^9^, their accuracy and applicability for cell state analysis^10^ remain obscure.

Here, we present Statescope, a comprehensive framework for capturing cell states. Statescope enables multi-omics deconvolution by integrating cell fractions derived from DNA data, followed by capturing inter-sample variations in cell type-specific gene expression profiles and cell states. Using bulk RNA-seq datasets with known gene expression profiles and cell fractions, as well as simulated bulk RNA-seq data from multiple scRNA-seq datasets, we systematically evaluate the Statescope framework, demonstrating its ability to correctly capture cell states for both malignant and non-malignant cells in tumor samples. Applying Statescope to The Cancer Genome Atlas (TCGA) cohorts, we identify previously recognized cell states, including neutrophil subpopulations in non-small cell lung cancer (NSCLC) and mixed malignant cell states in pancreatic ductal adenocarcinoma (PDAC). Statescope can transfer discovered cell states to new single-cell or bulk RNA-seq data. By transferring cell states discovered in the TCGA-NSCLC cohort to the randomized phase 2 POPLAR^11^ and phase 3 OAK^12^ immunotherapy trial cohorts^13^, we identify individual and combined cell states that can serve as predictive biomarkers for immunotherapy response.

## Results

### Statescope framework

**Fig. 1:**
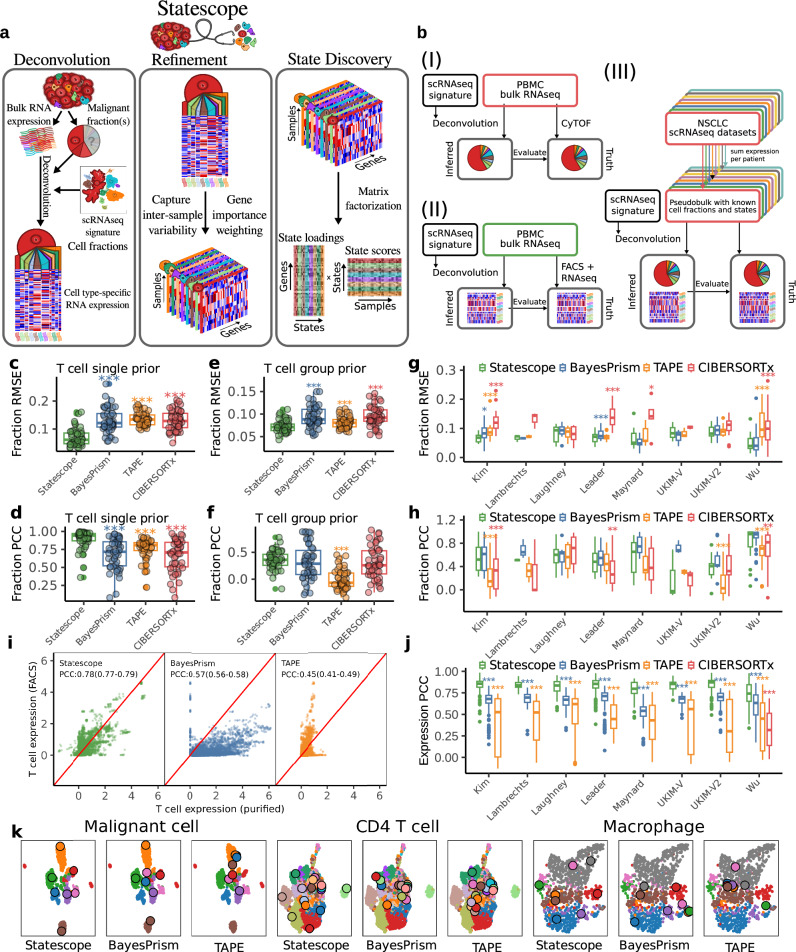
Statescope framework and performance benchmarking. Overview of the Statescope framework (**a**). Overview of the three gold-standard datasets 1, 2 and 3 to evaluate the performance of estimating cell type fraction and cell type-specific gene expression profiles (**b**). Performance of fraction estimation for respectively 10 (**c**, **d**) or 19 (**e**, **f**) cell types by Pearson Correlation Coefficient (PCC) and Root Mean Square Error (RMSE) calculated using predicted fractions and Cytometry by Time-Of-Flight (CyTOF) fractions in gold-standard dataset 1 (*n* = 49). Cell fraction estimation performance by PCC and RMSE calculated using predicted fractions and true fractions in gold-standard dataset 3 (*n* = 114 total) with 15 cell types across eight single-cell RNA sequencing (scRNA-seq) datasets (**g**, **h**). Performance for cell type-specific gene expression estimation measured by PCC using predicted T cell profiles and Fluorescence-Activated Cell Sorting (FACS) T cell profiles in gold-standard dataset 2 across eight samples (**i**). CIBERSORTx could not be applied in this evaluation due to the limited sample size. Performance for cell type-specific gene expression estimation measured by PCC per cell type using predicted cell type-specific gene expression profiles and true cell type-specific gene expression in gold-standard dataset 3 (*n* = 114 total) with 15 cell types across 8 scRNA-seq datasets (**j**). CIBERSORTx could only be applied in the Wu dataset due to limited sample sizes in the other datasets. UMAP visualizations of Malignant cell, CD4 T cell and Macrophage scRNA-seq profiles colored by different patients in the Maynard, Kim and Laughney datasets, respectively (**k**). Circled dots indicate the gene expression profiles predicted by Statescope, BayesPrism and TAPE projected onto the single cell UMAP space and are colored by patient. UMAP coordinates were computed using a KNN-graph using 15 neighbors constructed from the first 50 PCA components. Statistical significance in boxplot comparisons between Statescope (green) and other methods (BayesPrism, blue; TAPE, orange; CIBERSORTx, red) is determined using a one-sided, paired Wilcoxon rank-sum test and indicated as follows: *: *p* ≤ 0.05, **: *p* ≤ 0.005, ***: *p* ≤ 0.0005. Boxplots show RMSE or PCC distributions. The center line indicates the median (50th percentile). The box bounds represent the first and third quartiles (25th and 75th percentiles), defining the interquartile range (IQR). Whiskers extend to the minimum and maximum values within 1.5 × IQR of the quartiles. Points beyond this range are plotted individually, except for (**c**) and (**d**) where all sample PCCs are plotted individually as points. Source data for Fig. 1c–j are provided as a Source Data File. Abbreviations: PBMC Peripheral blood mononuclear cell, NSCLC Non-Small Cell Lung Cancer.

The Statescope framework consists of three computational steps: (I) multi-omic deconvolution, followed by (II) refinement of cell type-specific gene expression profiles to capture the inter-sample heterogeneity; and (III) cell state discovery (Fig. 1a). For an accurate deconvolution of bulk RNA profiles of tumors, we extended our previous Bayesian deconvolution algorithm, BLADE^6^, to integrate prior expectations of cell fractions, in particular, malignant fractions that can be determined from DNA copy number aberrations. The integration aids in overcoming the vast inter-patient gene expression heterogeneity of malignant cells that impedes accurate RNA deconvolution when applied to tumors. Multiple malignant states, such as classical, basal-like and also intermediate malignant subtypes in PDAC^14^, can also be addressed by integrating the overall malignant fraction as the prior expectation for the cumulative fraction of all malignant cell states (Supplementary Note 1). To facilitate the integration, the deconvolution module in Statescope iteratively optimizes the hyperparameter ${\alpha }_{i}^{t}$, which is not optimized in BLADE, to reflect the predetermined cell fractions ${f}_{i}^{t}$ when available (${\underline{{f}_{i}}}^{x}$; Supplementary Fig. 1a). Still, the Bayesian integration allows the final posterior estimates of cell fractions $({\hat{f}}_{i}^{t})$ to deviate from the prior expectation to reconstruct bulk gene expression profiles. The deconvolution step uses a prior expectation on cell type-specific gene expression profiles $({\nu }_{{ij}}^{t})$ to follow scRNA-seq signatures, which results in rather homogeneous estimates of cell type-specific gene expression profiles across the samples. Since inter-sample variation is a crucial component to discover cell states, a refinement step was introduced to allow for a deviation of the homogeneous profiles and capture the inter-sample heterogeneity of both malignant and non-malignant cells (Supplementary Fig. 1b). In this refinement step, residual bulk gene expression profiles are absorbed into cell type-specific gene expression profiles without altering cell fraction estimates. This is largely similar to BayesPrism for updating malignant cell profiles^5^, but in Statescope, it is applied to all cell types, including non-malignant ones. One additional parameter $({\omega }_{j}^{t})$ is inferred during the refinement step, which measures the inter-sample variability of cell type-specific gene expression profiles and hence is used as a gene importance measure to discover cell states. The refinement step that captures inter-sample variability now allows for state discovery. Thereto, the state discovery module was developed, which takes the cell type-specific gene expression profiles weighted by gene importance $({\omega }_{j}^{t})$ to capture various states within a cell-type using convex non-negative matrix factorization (cNMF; Supplementary Fig 1c)^15^. cNMF, a variant of NMF that can handle mixed-sign data seamlessly, identifies major patterns in cell type-specific gene expression profiles across samples and genes by summarizing them into state scores and state loadings. The state scores reflect the abundance of each state for each cell type in the samples. The state loadings reflect the gene signatures for each state and enable biological interpretation of individual states. Using the state loadings for the identified states, cNMF can also retrieve state scores from external cell-type-specific gene expression profiles, including scRNA-seq, for further validation and evaluation. To significantly improve computational time, we refactored the framework with the state-of-the-art Python library and GPU acceleration (PyTorch/CUDA)^16^ (Supplementary Note 2).

### Benchmarking of deconvolution and refinement steps in Statescope

We first evaluated the performance of Statescope to estimate cell type fractions and cell type-specific gene expression profiles, which is critical for accurate cell state analysis. Therefore, we used three gold standard datasets (Fig. 1b, Supplementary Table 1): 1) bulk RNA-seq profiles of 46 peripheral blood mononuclear cell (PBMC) samples for which we predetermined the true cell fractions by Cytometry by Time-Of-Flight (CyTOF) (Supplementary Note 3); 2) bulk RNA-seq profiles of 8 PBMC samples for which the true T cell profiles were determined by Fluorescence-Activated Cell Sorting (FACS) of T cells followed by RNA-seq by Hoek et al.^17^ ; and 3) 114 pseudobulk samples, which are in silico-generated mixtures from 8 independent non-small cell lung cancer (NSCLC) scRNA-seq studies with known true fractions and cell type-specific gene expression profiles (Kim et al.^18^, Lambrechts et al.^19^, Laughney et al.^20^, Leader et al.^21^, Maynard et al.^22^, UKIM and UKIM-V2 from Salcher et al.^3^, and Wu et al.^23^, Supplementary Table 1). As prior knowledge for deconvolution, we established signatures for individual cell types of PBMC and NSCLC from external scRNA-seq datasets (Stephenson et al.^24^ for PBMC and Zilionis et al.^25^ for NSCLC; Supplementary Table 2). The performance of Statescope to estimate cell type fractions was evaluated using the first and third gold standard datasets, and its ability to estimate cell type-specific gene expression profiles using the second and third datasets. We benchmarked Statescope against three state-of-the-art deconvolution algorithms as baseline methods, selected based on their capacity to estimate both cell type fractions and cell type-specific gene expression profiles: BayesPrism^5^, TAPE^7^, and CIBERSORTx^4^.

To benchmark fraction estimation, the first gold-standard dataset of PBMCs was used to deconvolute 10 cell types, including T cells. Statescope demonstrated superior performance compared to the three baseline methods when provided with the true T cell fraction as input to deconvolute 10 cell types (Fig. 1c, d, Supplementary Table 2). The Bayesian integration of the agglomerated, and thus heterogeneous, T cell fractions as an input showed a robust improvement in fraction estimation for all other cell types. The posterior T cell fraction consistently enhanced the prior fraction, even when noise was added to the prior knowledge (Supplementary Fig. 2). Using the same gold standard dataset, we also evaluated the ability of Statescope to estimate the fractions of 19 cell types by dividing the T cells into 10 T cell subsets while using the overall T cell fraction as a prior (T cell group prior, Supplementary Note 1). In this evaluation, Statescope outperformed all baseline methods in terms of absolute performance, as measured by the root-mean-squared error (RMSE) (Fig. 1e). In terms of relative performance, Statescope performed comparably to CIBERSORTx and BayesPrism as measured by the Pearson correlation coefficient (PCC) (Fig. 1f). In NSCLC pseudobulk deconvolution with the true malignant cell fractions as an input, Statescope often achieved the highest performance measured by both the RMSE (Fig. 1g) and PCC (Fig. 1h) across the eight scRNA-seq datasets comprising gold-standard dataset 3. The fraction estimation PCC was low for the UKIM-V and Lambrechts’ dataset, which was likely due to the limited number of samples (*n* = 3) (Supplementary Note 4). Nonetheless, it was frequently on par with other methods, particularly with BayesPrism.

We then evaluated Statescope to estimate cell-type-specific gene expression profiles. In PBMC T cell gene expression profiling using gold-standard dataset 2, Statescope achieved the highest concordance between predicted T cell profiles and FACS-sorted T cell profiles (PCC = 0.78 (range: 0.77–0.79)), significantly outperforming BayesPrism (PCC = 0.57 (range:0.56–0.58); one-sided Wilcoxon *p*-value = 0.003906) and TAPE (PCC = 0.45 (range 0.41–0.49); one-sided Wilcoxon *p*-value = 0.003906; Fig. 1i). Without prior T cell fraction information, Statescope still outperformed both methods (PCC = 0.73 (range: 0.72–0.74); one-sided Wilcoxon p-value = 0.0078). However, the addition of this prior information caused a significant improvement in performance (one-sided Wilcoxon p-value = 0.0078; Supplementary Fig. 3). In NSCLC transcriptome deconvolution with the gold-standard dataset 3, Statescope showed significantly higher concordance between predicted and true cell type-specific gene expression profiles compared to all baseline methods, with BayesPrism being the next best, outperforming TAPE and CIBERSORTx (Fig. 1j). When comparing predicted profiles with the single-cell profiles used to generate pseudobulk data, Statescope captured inter-sample variation in gene expression profiles of the same cell type most accurately, particularly for non-malignant cells (Fig. 1k). Cumulatively, Statescope achieved state-of-the-art performance in cell fraction estimation and significantly outperformed three state-of-the-art methods in recovering the cell type-specific gene expression profiles.

### Benchmarking of cell state recovery by Statescope

**Fig. 2:**
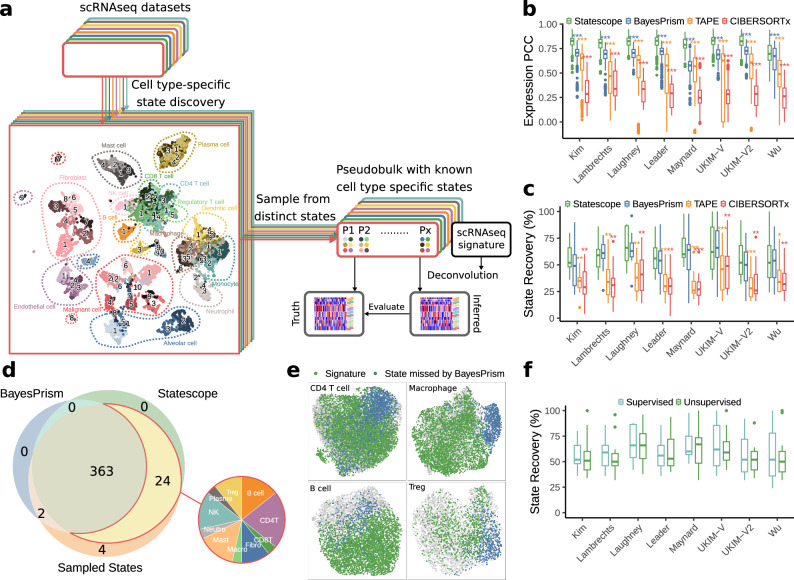
Benchmarking of cell state recovery. Overview of gold-standard Non-Small Cell Lung Cancer (NSCLC) dataset for cell state recovery benchmarking using 8 scRNA-seq NSCLC datasets (**a**). Example UMAP visualization depicting cells in the Maynard dataset where different cell states discovered are colored using Leiden graph-based clustering within 15 cell types (annotated by dashed lines). Benchmarking of cell type-specific gene expression estimation performance by Pearson Correlation Coefficient (PCC) per cell type calculated using predicted cell type-specific gene expression profiles and true cell type-specific gene expression in gold-standard NSCLC pseudobulk mixtures (*n* = 400 total, 50 per dataset) with 15 cell types across 8 scRNA-seq datasets (**b**). Benchmarking of state recovery in gold-standard NSCLC pseudobulk mixtures with 15 cell types across eight scRNA-seq datasets (**c**). The state recovery is determined by projecting predicted cell-type-specific gene expression profiles onto the pseudobulk scRNA-seq data and calculating the percentage of correctly transferred cell-state labels per cell type. Venn diagram depicting the overlap in states detected by Statescope, BayesPrism and the sampled states for pseudobulk dataset (**d**). The right pie-chart depicts the proportions of cell types among the 24 states that are only detected by Statescope but not by BayesPrism. UMAP visualizations of CD4 T cells, macrophages, B cells and regulatory T cells (Tregs) scRNA-seq profiles from the scRNA-seq signature (green) and from the pseudobulk datasets (cells in missed state; blue, other cells in pseudobulk; gray): Kim, UKIM-V, UKIM-V2 and Leader datasets respectively (**e**). UMAP coordinates are calculated using a KNN-graph with 15 neighbors calculated using the embeddings from Salcher et al. Comparison of state recovery by Statescope using the supervised (identical to **c**) and the unsupervised approach by cNMF of 15 cell types across 8 scRNA-seq datasets (**f**). Statistical significance in boxplot comparisons between Statescope (green) and other methods (BayesPrism; blue, TAPE; orange, CIBERSORTx; red) is determined using a one-sided, paired Wilcoxon rank-sum test and indicated as follows: *: *p* ≤ 0.05, **: *p* ≤ 0.005, ***: *p* ≤ 0.0005. Boxplots show PCC or State Recovery distributions. The center line indicates the median (50th percentile). The box bounds represent the first and third quartiles (25th and 75th percentiles), defining the interquartile range (IQR). Whiskers extend to the minimum and maximum values within 1.5 × IQR of the quartiles. Points beyond this range are plotted individually. Source data for Fig. 2b–d, f are provided as a Source Data File. Abbreviations: Treg Regulatory T cell.

We evaluated the ability of Statescope and the three aforementioned baseline methods to capture cellular states. To this end, we created a simulated dataset with known cell states using the data from eight NSCLC scRNA-seq studies of gold-standard dataset 3. From each of the eight scRNA-seq datasets, cell states were first delineated independently by clustering analysis, after which 1000 cells were sampled from specific combinations of cell states to generate 50 pseudobulk mixtures for each of the eight datasets (Fig. 2a, Supplementary Tables 1 and 2; see Methods).

We first assessed the concordance between the predicted and the true cell type-specific gene expression profiles for all four methods. Statescope showed the highest concordance, followed by BayesPrism, TAPE, and CIBERSORTx across all eight scRNA-seq datasets (Fig. 2b). Next, we performed a supervised cell state recovery analysis applicable to Statescope as well as all three baseline methods to calculate the percentage of correctly assigned cell states compared to the true scRNA-seq cell states (see Methods for the details). In this evaluation, Statescope and BayesPrism performed comparably, with both methods assigning the correct state to more than 50% of pseudobulk samples on average across all eight scRNA-seq datasets (Fig. 2c). TAPE and CIBERSORTx were significantly less accurate than Statescope and BayesPrism (paired one-sided Wilcoxon p-value < 8.97×10^-18^, TAPE versus Statescope across all scRNA-seq datasets). The supervised state recovery analysis revealed that most of the states defined in the eight scRNA-seq datasets were recovered by both Statescope and BayesPrism (363 states out of 393 states; Fig. 2d). However, Statescope achieved more complete cell state recovery, identifying 24 states that were missed by BayesPrism. Conversely, BayesPrism recovered two states that Statescope missed. All cell states missed by BayesPrism were non-malignant cell states with a gene expression profile distinct from the cells used for signature construction (Fig. 2e). With dedicated unsupervised state discovery using cNMF and the gene importance measure $({w}_{j}^{t})$, Statescope recovered the cell states comparably to the supervised approach (Fig. 2f). The gene importance measure $({w}_{j}^{t})$ highly correlates with the state specificity of genes expressed in each cell type, which contributed to the state discovery step (Supplementary Fig. 4). Overall, Statescope demonstrated the most accurate performance in recovering malignant and non-malignant cell states from bulk RNA-seq data, including those states unobserved in the scRNA-seq signature.

### Uncovering inter-tumoral heterogeneity in NSCLC with Statescope

**Fig. 3:**
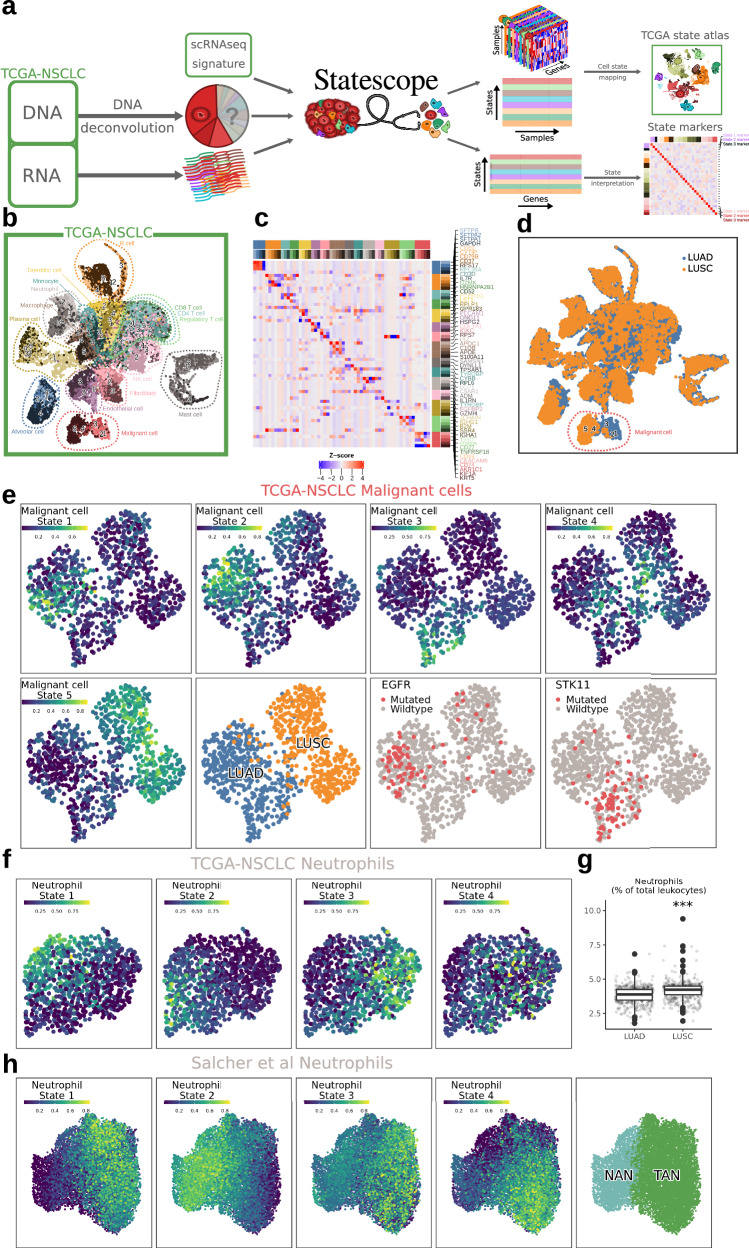
Application of Statescope to TCGA non-small cell lung cancer (NSCLC) cohort. Overview of Statescope application to the TCGA-NSCLC cohort (**a**). UMAP visualization of TCGA-NSCLC weighted cell type-specific gene expression profiles colored by cell states identified by Statescope within 15 cell types (annotated by dashed lines) (**b**). Cell states are discretized by taking the cell state with the highest score for each sample and cell type. UMAP coordinates were computed using a KNN-graph using 10 neighbors derived from the first 50 PCA components based on the top 1000 highly variable genes. Heatmap of top state loadings for each state discovered by Statescope. State loadings were z-score transformed for visualization (**c**). TCGA-NSCLC atlas colored by subtype, lung adenocarcinoma (LUAD), red; lung squamous cell carcinoma (LUSC), green (**d**). Analysis of TCGA-NSCLC malignant cell states (**e**). Analysis of TCGA-NSCLC neutrophil cell state scores (**f**). Neutrophil fraction of the total leukocytes in TCGA-LUAD and TCGA-LUSC samples (*n* = 973) as predicted by Statescope (**g**). Boxplots show neutrophil fraction distributions. The center line indicates the median (50th percentile). The box bounds represent the first and third quartiles (25th and 75th percentiles), defining the interquartile range (IQR). Whiskers extend to the minimum and maximum values within 1.5 × IQR of the quartiles. Points beyond this range are plotted individually. Statistical significance was determined by a one-sided Wilcoxon rank-sum test and indicated as follows ***: *p* ≤ 0.0005 with the exact p-value being smaller than 2.2×10^-16^. Neutrophil states retrieved in annotated scRNA-seq data from Salcher et al. (**h**). UMAP coordinates were computed using a KNN-graph with 15 neighbors derived using the embeddings from Salcher et al.^3^. The normal-associated neutrophil (NAN)/tumor-associated neutrophil (TAN) phenotypes assigned by Salcher et al.^3^ are shown in the rightmost panel. Source data for Fig. 3c, g are provided as a Source Data File. Abbreviations: NK Natural Killer cell.

Statescope was applied to characterize interpatient cell state heterogeneity for NSCLC using The Cancer Genome Atlas (TCGA) dataset (*N* = 973)^26,27^ (Fig. 3a, Supplementary Table 1). First, we constructed prior knowledge of scRNA-seq signatures from the NSCLC scRNA-seq atlas by Salcher et al.^3^ (Supplementary Table 2) and predetermined malignant fractions for each of the TCGA samples from the DNA copy number aberrations with ACE^28^ (see Methods for details). We confirmed that the ACE tumor fraction estimates were highly concordant with orthogonal, variant allele frequency-based estimates derived using ABSOLUTE^29^ (Supplementary Note 6). Notably, the empirical deviation between these two methods was smaller than the ‘low-noise’ condition modeled in our PBMC T-cell benchmarking, confirming the high reliability of the DNA-derived priors for the application of Statescope to real-world tumor data. We then applied Statescope to identify and interpret cell states across cell types. In addition, we annotated and validated cell states by retrieving them in the NSCLC scRNA-seq atlas^3^.

The TCGA-NSCLC cell state atlas consists of a total of 53 cell states across 15 cell types (Supplementary Table 3), where the corresponding state loadings capture the top cell state markers (Fig. 3b, c). As anticipated, the atlas showed that the biggest difference between two histological subtypes, lung adenocarcinoma (LUAD) and squamous cell carcinoma (LUSC), resides in the malignant cells (Fig. 3d). Statescope identified five malignant cell states: three states enriched within LUAD (malignant cell states 1–3), one mixed LUAD/LUSC state (malignant cell state 4) and one state enriched within LUSC samples (malignant cell state 5) (Fig. 3e). The LUSC state (malignant cell state 5) is defined by well-known LUSC markers according to the corresponding state loadings (*KRT5*, *KRT6A*, and *KRT14*; Supplementary Table 3)^30^. The three LUAD states showed associations with driver mutation status; malignant cell state 2 has a significantly higher score in EGFR-mutated patient samples (*p* < 2.2×10^-16^), while malignant cell state 3 is enriched in STK11-mutated (*p* < 2.2×10^-16^) and KRAS-mutated tumors (*p* = 0.0006). These observations are in line with previous scRNA-seq studies demonstrating that LUAD tumors with different mutations exhibit distinct malignant cell states^23,31,32^.

Statescope also identified diverse non-malignant cell states, which align with previous literature of single-cell analysis^3,23,33,34^ (Supplementary Figs. 5, 6, Supplementary Table 3). Many of these states are differentially enriched between LUAD and LUSC^33^, including ‘adventitial fibroblasts’ (Fibroblast state 1) in LUAD^34^ and ‘tissue-resident macrophages’ (Macrophage state 3) in LUSC^33^ (Supplementary Fig. 6). For neutrophils, a cell type often underrepresented in scRNA-seq analysis^3^, Statescope collectively reproduced the findings of scRNA-seq data that was generated with a dedicated protocol to enrich for neutrophils^3^ (Fig. 3f). First, Statescope recapitulated higher neutrophil fractions in LUSC compared to LUAD (*p* < 2.2×10^-16^)^3,23^ (Fig. 3g). Retrieval of neutrophil state scores in the Salcher et al.^3^ neutrophil profiles revealed that the four neutrophil states align with normal-associated neutrophils (NANs; state 2) and tumor-associated neutrophils (TANs; the other states) (Fig. 3h). A complete list of cell state annotations can be found in Supplementary Table 3. In summary, Statescope captures diverse malignant and non-malignant cell states from bulk RNA-seq data, including those that can only be identified through dedicated scRNA-seq protocols.

### Uncovering intra-tumoral heterogeneity in PDAC with Statescope

**Fig. 4:**
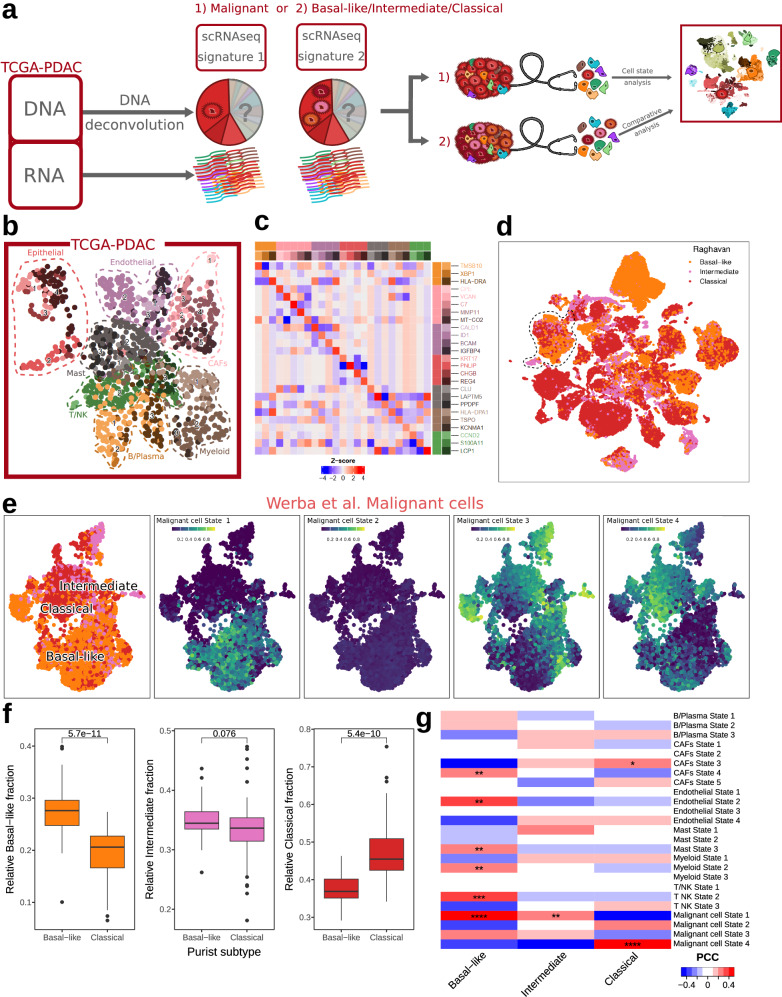
Application of Statescope to pancreatic ductal adenocarcinoma (PDAC) to recapitulate inter- and intra-tumoral heterogeneity. Overview of Statescope applications to the TCGA-PDAC dataset, using two signatures: signature 1, with a single malignant cell type and signature 2 with malignant cells further delineated into the basal-like, intermediate & classical subtypes (**a**). UMAP visualization of TCGA-PDAC weighted cell type-specific gene expression profiles colored by cell states discovered using Statescope within seven cell types (annotated by dashed lines). Cell states were discretized by taking the state with the highest score for each sample and cell type. UMAP coordinates were computed using a KNN-graph using 10 neighbors derived from the first 50 PCA components based on the top 1000 highly variable genes (**b**). Heatmap of top state loading for each state discovered by Statescope. State loadings were z-score transformed for visualization (**c**). PDAC malignant single cell atlas colored by tumor subtype (Basal-like, orange; Intermediate, pink; Classical, red) (**d**). Analysis of TCGA-PDAC malignant cell state scores in scRNA-seq of malignant cells from one patient with all three subtypes in Werba et al.^36^ (**e**). Comparison of the relative tumor subtype fractions with bulk RNA (*n* = 166) subtype labels. Differences between Purist subtypes were tested by two-sided Mann-Whitney U test, p-values are indicated in the plot (**f**). Boxplots show relative distributions of tumor subtype fractions. The center line indicates the median (50th percentile). The box bounds represent the first and third quartiles (25 and 75th percentiles), defining the interquartile range (IQR). Whiskers extend to the minimum and maximum values within 1.5 × IQR of the quartiles. Points beyond this range are plotted individually. Heatmap of Pearson correlation of relative tumor subtype fractions with state scores (**g**). Asterisks indicate two-sided uncorrected p-values of the Pearson Correlation Coefficient (PCC) with * = *p* < 0.05, ** = *p* < 0.01, *** = *p* < 0.001, **** = *p* < 0.0001. Source data for Fig. 4c, f, g are provided as a Source Data File. Abbreviations: NK Natural Killer cell, CAFs Cancer-Associated Fibroblasts.

We then applied Statescope to the TCGA-PAAD cohort (*n* = 166)^35^ to assess its capability to recapitulate the profound intra-tumoral heterogeneity of the malignant cells in PDAC, well-established by single-cell studies^36–38^ (Fig. 4a, Supplementary Table 1). As prior knowledge, we constructed two separate PDAC scRNA-seq signatures from Werba et al.^36^: 1) seven cell types signature including one malignant cell type and 2) nine cell type signature further delineating malignant cells into classical, basal-like, and intermediate subtypes^37^ (Supplementary Table 2, Supplementary Note 1), along with malignant fractions from DNA copy number aberrations with ACE^28^.

Statescope revealed 25 cell states across seven PDAC cell types (Fig. 4b, Supplementary Table 4), defined by their respective top cell state markers (Fig. 4c). Statescope identified four malignant cell states: state 1 defined by basal-like markers^37^ (S100A2, KRT7/KRT17), state 2 defined by exocrine/acinar markers^14^ (PNLIP, CPA1/CPB1), state 3 defined by neuroendocrine differentiation markers^39^ (CHGA, CHGB) and state 4 defined by classical state markers^37^ (REG4, TFF1, CEACAM5 and CLDN18) according to the state loadings (Supplementary Table 4). In addition, state 1 and state 4 showed high state loadings for single-cell-based basal-like and classical marker genes^37^, respectively (Supplementary Fig. 7a), and their state scores were higher in basal-like and classical subtypes classified by bulk RNA-seq analysis^40^ (Supplementary Fig. 7b). Furthermore, basal-like and classical malignant states retrieved in the PDAC scRNA-seq data^36^ (Fig. 4d) align with the basal-like/classical state scores estimated by Statescope (Fig. 4e, Supplementary Fig. 7c). On the other hand, neuroendocrine state scores were high in intermediate cells. A complete list of all cellular states, including non-malignant cell states such as pericyte, iCAF (inflammatory CAF) and myCAF (myofibroblastic CAF) fibroblast states (Supplementary Fig. 8) is provided in Supplementary Table 4.

We further verified the malignant cell states by estimating relative fractions of classical, basal-like, and intermediate subtypes using Statescope with a signature that explicitly delineates these malignant subtypes. The relative basal-like and classical fractions, which significantly correlated with the corresponding bulk RNA-based subtype classification (*p* = 5.7×10^-11^ and *p* = 5.4×10^-10^ respectively; Fig. 4f), verified the basal-like/classical state scores determined using the signature with one malignant cell type (correlation test; *p* = 8.7×10^-12^ and *p* = 3.3×10^-14^; Fig. 4g). We found no correlation of the intermediate subtype with either basal-like or classical bulk RNA-based subtypes (Fig. 4f), but it was significantly associated with the basal-like state (Fig. 4g). When comparing the three malignant subtypes and non-malignant cell states, we identified the following significant associations (Bonferroni adjusted p-value < 0.05, Pearson correlation > 0.25): 1) classical subtype with the iCAF state and 2) basal-like subtype with tumor endothelial cell (TEC), proliferating mast cell, activated macrophage, activated T cell and myCAF states. Among these, spatial co-occurrence between basal-like malignant cells and myCAF states was previously described^41^. No significant association was found for the intermediate subtype. Cumulatively, the intra-tumoral heterogeneity uncovered by Statescope enables a deeper investigation into associations between malignant and non-malignant cell states, providing insights into the tumor microenvironment.

### Associating cellular states in NSCLC with patient outcome

A key potential application of Statescope lies in leveraging the common availability of bulk RNA and DNA-seq data from clinical cohorts, which allows linking cell states with patient outcomes. Therefore, using the TCGA-NSCLC cell state atlas (Fig. 3a–c) as a reference, we applied Statescope and retrieved the 53 cell states for two independent advanced-stage NSCLC cohorts: the Hartwig Medical Foundation cohort that covers RNA and DNA data of tumor samples from real-world patients treated with diverse therapies^42^ (HMF cohort; *n* = 119 of which *n* = 67 had available OS status) and data from two randomized clinical trials of PD-L1 inhibitor atezolizumab versus docetaxel chemotherapy^11–13^ (POPLAR/OAK cohort; *n* = 891).

**Fig. 5:**
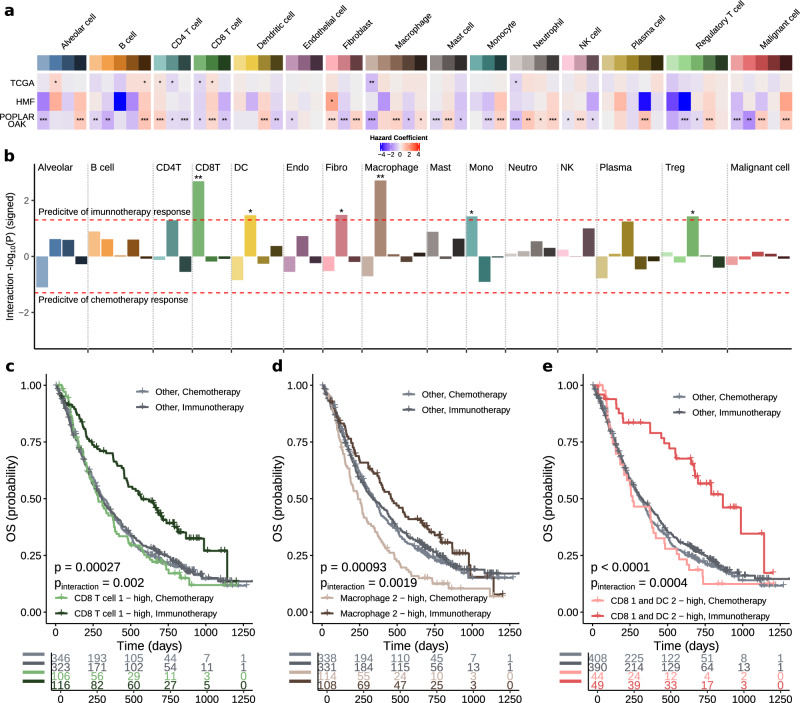
Prognostic and predictive value of cell states from Statescope. Prognostic value of all cell states (*n* = 53) across 15 cell types in the TCGA, HMF, and POPLAR/OAK cohorts (**a**). Hazard coefficients (graded colors blue negative, red positive, z-score transformed) and uncorrected p-values (asterisks) were calculated using univariate analysis of 4-year overall survival (OS) of continuous state scores with Cox regression. Predictive value of state scores for treatment response in POPLAR/OAK (**b**). The y-axis denotes the signed uncorrected -log10(p-values) of the interaction term between categorized state scores and the treatment received using Cox regression of OS. The red dashed line indicates the significance threshold of 0.05. Kaplan-Meier curves of OS for patients with ‘high’ (greens) and without ‘high’ (grays) CD8 T cell state 1 scores receiving chemotherapy (light shading) or immunotherapy (dark shading) (**c**). Kaplan-Meier curves of OS for patients with ‘high’ (browns) and without ‘high’ (grays) macrophage state 2 scores receiving chemotherapy (light shading) or immunotherapy (dark shading) (**d**). Kaplan-Meier curves of OS for patients with ‘high’ (browns) and without ‘high’ (grays) CD8 T cell state 1 and dendritic cell state 2 scores receiving chemotherapy (light shading) or immunotherapy (dark shading) (**e**). P-values in Kaplan-Meier plots indicate global significance determined by the log-rank test. The *p*_interaction_ value indicates the significance of the interaction term in the multivariate Cox model with the state and treatment. The number of patients without an event at different time points are indicated for the groups in the risk tables below the Kaplan-Meier plots. State scores are categorized into ‘high’ by selecting the upper 75% quantile. Statistical significance of Cox regression analysis is indicated as follows: *: *p* ≤ 0.05, **: *p* ≤ 0.005, ***: *p* ≤ 0.0005. Source data for Fig. 5a, b are provided as a Source Data File. Abbreviations: DC Dendritic cell, Endo Endothelial cell, Fibro Fibroblast, Mono Monocyte, Neutro Neutrophil, NK Natural Killer cell, Treg Regulatory T cell.

We first evaluated the prognostic value of individual cell states by univariate analysis of 4-year overall survival (OS) using Cox regression across the three cohorts. In 33 out 53 states (62%), the sign of the hazard coefficient was concordant for all three cohorts (Fig. 5a). Furthermore, in 44 out of 53 (83%) states, the sign of the hazard coefficient was concordant between the advanced-stage cohorts (HMF, POPLAR/OAK), suggesting that the prognostic associations of some cell states may potentially differ based on disease stage or treatment context. States significantly associated with improved OS (*p*-value < 0.05) in more than one cohort included ‘activated CD4 T cells’ (CD4 T cell state 2), ‘effector CD8 T cells’ (CD8 T cell state 1), ‘inflammatory macrophages 1’ (Macrophage state 1), and ‘TANs’ (Neutrophil state 1). States significantly associated with worse OS across more than one cohort included ‘proliferating B cells’ (B cell state 5), ‘proliferating CD4 T cells’ (CD4 T cell state 1), ‘exhausted CD8 T cells’ (CD8 T cell state 2), and ‘myofibroblasts’ (Fibroblast state 1). In the HMF cohort, only the ‘myofibroblast’ (Fibroblast state 1) state reached significance for association with OS, presumably due to the limited sample size (*n* = 67 with OS available).

Leveraging the POPLAR/OAK cohort, we next sought to identify cell states predictive of benefit from immunotherapy with PD-L1 inhibitor atezolizumab compared to chemotherapy with docetaxel. In Cox regression analysis of OS, we found significant interactions between PD-L1 inhibition outcome and multiple cell states (*p* < 0.05, Fig. 5b), including ‘effector CD8 T cells’ (Fig. 5c, CD8 T cell state 1), ‘lipid-associated macrophages 1’ (Fig. 5d, macrophage state 2), ‘cDCsI/II-2’ (dendritic cell state 2), ‘adventitial fibroblasts’ (fibroblast state 2), ‘monocytes-1’ (monocyte state 1), and ‘regulatory-2 Tregs’ (regulatory T cell state 3) (Supplementary Fig. 9). Among these, the ‘effector CD8 T cell’ state, defined by the expression of effector molecules (e.g., GZMK/GZMH) which are essential for an immune response, presented the highest predictive value (interaction *p*-value = 0.00196), with patients having high state scores experiencing a two-year OS of 53% in the PD-L1 inhibition group and 24% in the chemotherapy group (Fig. 5c).

When several predictive states are combined, a stronger interaction with PD-L1 inhibition treatment is observed, indicating their complementarity (Supplementary Fig. 10). Among these, the highest predictive value was observed when combining ‘effector CD8 T cell’ and ‘cDCsI/II-2’ states (interaction *p*-value = 0.00044), which might reflect MHC class I-mediated cytotoxic activation of CD8 T cells by antigen-presenting cDCs. Patients with high scores for both states experienced a two-year OS of 57% in the PD-L1 inhibition group compared to 19% in the chemotherapy group (Fig. 5e). Cumulatively, these results demonstrate that cell states derived by Statescope can be clinically relevant for prognosis and prediction of treatment benefit.

## Discussion

Statescope is a comprehensive framework that can accurately capture cellular composition and states from bulk RNA-seq data by integrating predetermined cell fractions from additional modalities, such as DNA-based data from tumors and prior knowledge of cell type-specific gene expression from scRNA-seq. It delivers state-of-the-art performance in cell fraction estimation and, moreover, consistently enhances cell state identification compared to the baseline methods, even for states not covered in the scRNA-seq data used as prior knowledge (also see Supplementary Note 5). Beyond capturing inter-patient heterogeneity in cell states, Statescope also enables the characterization of intra-tumoral co-occurring states and their relative proportions, as demonstrated for PDAC. A flexible cNMF-based cell state approach enables Statescope to validate and interpret captured cell states from scRNA-seq data, and to transfer them to new cohorts using only bulk RNA-seq, thereby enabling robust capture of cell states and their associations with patient outcomes.

Several considerations are important when applying Statescope. First, a critical parameter in deconvolution is the definition of cell types and selection of marker genes in scRNA-seq signatures^43^. Cell types not present in the sample, and hence not contributing to the bulk RNA profiles, pose a challenge, as Statescope restricts assigning a fraction of zero to a cell type due to its optimization algorithm. Conversely, if an abundant cell type is omitted, Statescope will reassign its signal to the phenotypically most similar cell type, rather than omitting the signal. For example, in our PDAC application, we identified a pericyte state among the fibroblast states and a plasma cell state within the B cell states. These states were not explicitly defined as separate cell types in the signature, but their transcriptomic profiles emerged as distinct states within the most closely resembling cell types^36^. Another important point is the quality of prior knowledge. Although Bayesian modeling provides robustness to errors, higher-quality prior knowledge, such as scRNA-seq-derived signatures and predetermined cell fractions, will enhance overall performance. For instance, malignant fractions predetermined from DNA copy number aberrations with ACE^28^, used in this study, can be more precise if ploidy is accurately determined. Furthermore, we recommend interpreting the output of Statescope in relative rather than absolute terms. Cell fractions and state scores are best understood in comparison across samples, rather than as standalone absolute values. Along the same lines, state discovery by cNMF identifies states by recognizing relative differences between cell type-specific gene expression profiles and thus requires a sufficient number of samples, as in any unsupervised clustering analysis, to discover states. Finally, to translate potential cell state biomarkers into clinical use, further studies are needed to establish dedicated thresholds for the state scores to assign specific cell states to individual patients, accounting for relevant clinical parameters.

Statescope opens the door to a broad range of applications. Prior cell fractions can be derived from various modalities, including histology, cell sorting, and DNA-based analytics^28,29^. As demonstrated in the PDAC application, this prior knowledge can be given to multiple co-occurring malignant subtypes instead of one malignant cell type considered in deconvolution. In oncology, RNA and DNA sequencing are frequently available for large patient cohorts^26,27,35,42,44^ and clinical trials^11–13,45^, creating ideal conditions for deploying Statescope. Furthermore, the framework shows promise for use in spatial transcriptomics^41,46^, where additional priors can be obtained from matched cellular images like histology images. In the future, AI-powered image analysis could enhance Statescope by providing accurate, spatially resolved fractions of additional cell types and compositions^47,48^. Overall, we envision Statescope as a robust and adaptable framework for cell state analysis using widely available bulk RNA-seq data, capable of generating insights into the dynamic and heterogeneous cellular environments within (tumor-) tissues.

## Methods

### Ethical statement

This research complies with all relevant ethical regulations. The majority of the molecular data analyzed in this study were obtained from publicly available repositories, as detailed in the Data Availability section. For one of the benchmark datasets, we generated novel RNA-seq data from peripheral blood mononuclear cell (PBMC) samples. These PBMC samples were collected at Amsterdam UMC, location VU University Medical Center (Amsterdam, The Netherlands) after approval from The Medical Ethics Review Committee of Amsterdam UMC (protocol number 2012/362) and written informed consent.

### Statescope deconvolution module

#### Bayesian deconvolution by Statescope

In Statescope, bulk RNA deconvolution is performed using the Bayesian deconvolution framework BLADE^6^. In BLADE, the bulk gene expression level of each gene *j* in sample *i* is modeled by ${y}_{{ij}}=\log ({\sum }_{t}{{f}_{i}}^{t}\exp ({{x}_{{ij}}}^{t}))+{\epsilon }_{{ij}}$. The hidden variables ${{f}_{i}}^{t}$ and ${{x}_{{ij}}}^{t}$ respectively denote the fraction of cell type *t* for sample *i* and the expression of gene *j* for sample *i* in cell type *t*. The probabilistic assumption for these variables is:

1 $${\rm{P}}\left({y}_{{ij}}|{{x}_{{ij}}}^{1},{{f}_{i}}^{1},\cdots,{{x}_{{ij}}}^{T},{{f}_{i}}^{T},\sigma \right)={\mathcal{N}}({y}_{{ij}}|{\sum }_{t}{{f}_{i}}^{t}\exp \left({{x}_{{ij}}}^{t}\right),\sigma )$$

2 $${\rm{P}}\left({{x}_{{ij}}}^{t}|{{\mu }_{j}}^{t},\frac{1}{{{\lambda }_{j}}^{t}}\right)={\mathcal{N}}\left({{x}_{{ij}}}^{t}|{{\mu }_{j}}^{t},\frac{1}{{{\lambda }_{j}}^{t}}\right)$$

3 $${\rm{P}}\left({{f}_{i}}^{t}|{{\alpha }_{i}}^{t}\right)={\mathcal{D}}\left({{f}_{i}}^{t}|{{\alpha }_{i}}^{t}\right)$$

where N() and D() represent Gaussian and Dirichlet distributions, respectively. With this formulation, ${y}_{{ij}}$ and ${{x}_{{ij}}}^{t}$ follow a log-normal distribution in linear scale ($\exp ({y}_{{ij}})$ and $\exp ({{x}_{{ij}}}^{t})$). To incorporate prior knowledge from scRNA-seq data, a conjugate prior distribution for ${{x}_{{ij}}}^{t}$ is introduced:

4 $${\rm{P}}\left({{\mu }_{j}}^{t},\frac{1}{{{\lambda }_{j}}^{t}}|{{\mu }^{t}}_{0j},{{\kappa }^{t}}_{0j},\,{{\alpha }^{t}}_{0j},\,{{\beta }^{t}}_{0j}\right)={\mathcal{N}}\left({{\mu }_{j}}^{t}|{{\mu }^{t}}_{0j},\frac{1}{{{\kappa }^{t}}_{0j}{{\lambda }_{j}}^{t}}\right){\mathcal{G}}\left({{\lambda }_{j}}^{t}|{{\alpha }^{t}}_{0j},\,{{\beta }^{t}}_{0j}\right)$$

where G() is the Gamma distribution. The hyperparameters, $\theta=\{{{\mu }^{t}}_{0j},{{\kappa }^{t}}_{0j},\,{{\alpha }^{t}}_{0j},\,{{\beta }^{t}}_{0j}\}$ are the gene expression signatures derived from scRNA-seq where they reflect cell-type-specific gene expression profiles $({{\mu }^{t}}_{0j})$ and their gene expression variability $({{\kappa }^{t}}_{0j},\,{{\alpha }^{t}}_{0j},\,{{\beta }^{t}}_{0j})$.

For inference of hidden variables, a collapsed variational inference is employed, which approximates the posterior distribution of the hidden variables using variational distributions:

5 $${\rm{Q}}\left({{x}_{{ij}}}^{t}|{{v}_{{ij}}}^{t},{{w}_{j}}^{t}\right)={\mathcal{N}}\left({{x}_{{ij}}}^{t}|{{v}_{{ij}}}^{t},{{w}_{j}}^{t}\right)$$

6 $${\rm{Q}}\left({{f}_{i}}^{t}|{\beta }_{i}^{t}\right)={\mathcal{D}}\left({{f}_{i}}^{t}|{\beta }_{i}^{t}\right)$$

with variational parameters $\vartheta=\{{{v}_{{ij}}}^{t},{{w}_{j}}^{t},{\beta }_{i}^{t}\}$. Then, the evidence lower-bound (ELBO) which serves as the objective function of BLADE is:

7 $$	 \int {\rm{Q}}\left({\bf{X}},{\bf{F}}\right)\log \frac{{\rm{P}}\left({\bf{Y}},{\bf{X}},{\bf{F}} | {\rm{\theta }}\right)}{{\rm{Q}}\left({\bf{X}},{\bf{F}} | {\rm{\vartheta }}\right)}{\rm{dXdF}}={{\rm{E}}}_{{\rm{Q}}}\left[\log {\rm{P}}\left({\bf{Y}},{\bf{X}},{\bf{F}} | {\rm{\theta }}\right)\right]\\ 	 -{{\rm{E}}}_{{\rm{Q}}}\left[\log {\rm{Q}}\left({\bf{X}},{\bf{F}} | {\rm{\vartheta }}\right)\right]={{\rm{E}}}_{{\rm{Q}}}\left[\log {\rm{P}}\left({\bf{Y}},{\bf{X}},{\bf{F}} | {\rm{\theta }}\right)\right]+{{\rm{E}}}_{{\rm{Q}}}\left[\log {\rm{P}}\left({\bf{X}} | {\rm{\theta }}\right)\right] \\ 	+{{\rm{E}}}_{{\rm{Q}}}\left[\log {\rm{P}}\left({\bf{F}} | {\rm{\alpha }}\right)\right]-{{\rm{E}}}_{{\rm{Q}}}[\log {\rm{Q}}({\bf{X}}|{\rm{\vartheta }})]-{{\rm{E}}}_{{\rm{Q}}}[\log {\rm{Q}}({\bf{F}}|{\rm{\vartheta }})].$$

#### Integration of expected cell fractions by optimizing the hyperparameter ${\alpha }_{i}^{t}$

In the Statescope framework, the BLADE algorithm^6^ is extended to optimize hyperparameter $\alpha=\{{\forall }_{i,t}{\alpha }_{i}^{t}\}$ along with the variational parameters. This extension is introduced to facilitate accurate deconvolution in the presence of cell types with large gene expression variability (e.g., malignant cells). The above ELBO function can be represented as follows:

8 $${\rm{ELBO}}={{\rm{E}}}_{{\rm{Q}}}[\log {\rm{P}}({\bf{F}}|{\rm{\alpha }})]+{\rm{const}}$$

The *const* term refers to the part of the ELBO function that is not influenced by ${\alpha }_{i}^{t}$. Given the ELBO above, the partial derivative of the ELBO with respect to ${\alpha }_{i}^{t}$ is:

9 $$\frac{\partial }{\partial {\alpha }_{i}^{t}}{\rm{ELBO}}={\rm{\phi }}\left({\beta }_{i}^{t}\right)-{\rm{\phi }}\left({\alpha }_{i}^{t}\right)-\left({\rm{\phi }}\left({\sum }_{t}{\beta }_{i}^{t}\right)-{\rm{\phi }}\left({\sum }_{t}{\alpha }_{i}^{t}\right)\right)$$

where ${\rm{\phi }}()$ represents the digamma function. The above formula is 0 when ${\alpha }_{i}^{t}={\beta }_{i}^{t}$ and thus, a closed form update rule of assigning the current ${\beta }_{i}^{t}$ to ${\alpha }_{i}^{t}$ will be maximizing ELBO with respect to ${\alpha }_{i}^{t}$. A constraint is imposed to share the same values for ${\alpha }_{i}^{t}$ across the samples to reduce the number of free parameters to gain overall robustness. With this constraint, the updating rule for ${\alpha }_{i}^{t}$ becomes ${\alpha }_{i}^{t}=\frac{{\beta }_{i}^{t}}{{\sum }_{i}{\beta }_{i}^{t}}$. In addition, an extra constraint is imposed on ${\alpha }_{i}^{t}$ if there is cell fraction given as a known prior for a cell type, ${\hat{f}}_{i}^{t}$, such that ${\hat{f}}_{i}^{t}=\frac{{\alpha }_{i}^{t}}{{\sum }_{t}{\alpha }_{i}^{t}}$ or for a group of cell types, ${\hat{f}}_{i}^{G}\left(t\in G\subset T\right)$, such that ${\hat{f}}_{i}^{G}=\frac{{\sum }_{t\in G}{\alpha }_{i}^{t}}{{\sum }_{t}{\alpha }_{i}^{t}}$. To fulfill this constraint, ${\alpha }_{i}^{t}$ is updated in the following steps:

1. Initial update of ${\alpha }_{i}^{t}=\frac{{\beta }_{i}^{t}}{{\sum }_{i}{\beta }_{i}^{t}}$.
2. Calculate the fractions using updated ${\alpha }_{i}^{t}$: ${f}_{i}^{t}=\frac{{\alpha }_{i}^{t}}{{\sum }_{t}{\alpha }_{i}^{t}}$.
3. (If there is any cell type with a known fraction) Adjust fractions according to the prior knowledge. Specifically, set ${f}_{i}^{t}={\hat{f}}_{i}^{t}$ or ${\sum }_{t\in G}{f}_{i}^{t}={\hat{f}}_{i}^{G}$ for cell types with prior knowledge, and normalize the fraction such that ${\sum }_{t}{f}_{i}^{t}=1$.
4. (If there is any cell type with a known fraction) Adjust ${\alpha }_{i}^{t}$ taking the adjusted fraction as follows: ${\hat{\alpha }}_{i}^{t}={f}_{i}^{t}\left({\sum }_{t}{\alpha }_{i}^{t}\right).$

The adjustment of ${\alpha }_{i}^{t}$ to meet the prior expectation in steps 3-4 is an efficient heuristic constraint without complicating the variational inference framework. The above update rule for ${\alpha }_{i}^{t}$ is done in an iterative manner until model convergence or once the maximum number of iterations is reached (default = 100).

#### PyTorch implementation of Statescope

The deconvolution with BLADE exhibits high computational complexity as the number of parameters increases substantially with the number of samples, cell types and genes. Therefore, in the Statescope deconvolution module, Statescope is refactored to perform computations using PyTorch integrated with CUDA^16^. This strategy allows acceleration using GPU hardware and speeds up optimization significantly (See Supplementary Note 2).

### Statescope refinement module

#### Further optimization of cell type-specific gene expression profiles ${\hat{x}}_{{ij}}^{t}$

An extra step is introduced to further optimize the posterior estimates of cell-type-specific gene expression profiles, ${\hat{x}}_{{ij}}^{t}$, where extra genes that were not used in the initial optimization can be added. The part of ELBO that concerns ${x}_{{ij}}^{t}$ can be represented as follows:

10 $${\rm{ELBO}}={{\rm{E}}}_{{\rm{Q}}}\left[\log {\rm{P}}\left({\bf{Y}}|{\bf{X}},{\bf{F}},\sigma \right)\right]+{{\rm{E}}}_{{\rm{Q}}}\left[\log {\rm{P}}\left({\bf{X}}|{\rm{\theta }}\right)\right]-{{\rm{E}}}_{{\rm{Q}}}\left[\log {\rm{Q}}\left({\bf{X}}|{\rm{\vartheta }}\right)\right]+{\rm{const}}.$$

In this formula, only the variational parameters for ${x}_{{ij}}^{t}$ (${v}_{{ij}}^{t}$ and ${w}_{j}^{t}$) are updated. The first two components in the ELBO function reflect reconstruction performance of bulk gene expression profile $\left({{\rm{E}}}_{{\rm{Q}}}\left[\log {\rm{P}}\left({\bf{Y}}|{\bf{X}},{\bf{F}},\sigma \right)\,\right]\right)$ and concordance with single-cell prior knowledge $\left({{\rm{E}}}_{{\rm{Q}}}\left[\log {\rm{P}}\left({\bf{X}}|{\rm{\theta }}\right)\right]\right)$, respectively. To address interpatient heterogeneity during the refinement step, we relax the model’s reliance on prior cell-type-specific gene expression profiles by down-weighting the second term of the objective function, ${{\rm{E}}}_{{\rm{Q}}}\left[\log {\rm{P}}\left({\bf{X}}|{\rm{\theta }}\right)\right]$, by a factor of 100. This scaling factor is derived from the empirical observation that the second term is typically 15–20 times larger than the first term. This size difference arises because the second term evaluates a much larger parameter space (Genes × Samples × Cell Types) than the first term (Genes × Samples). During the initial deconvolution, this magnitude difference is advantageous as it strictly enforces adherence to the single-cell prior knowledge. However, to capture patient-specific tumor variations, the model must be allowed flexibility. By applying the $\frac{1}{100}$ scaling factor, the first term (representing bulk data reconstruction) becomes roughly 5–6 times larger than the second term. This shift in the optimization landscape allows ${x}_{{ij}}^{t}$ to deviate from the reference single-cell profiles, absorbing sample-specific residuals in the bulk gene expression data and thereby achieving a more accurate, patient-specific reconstruction.

Since the above ELBO only concerns ${\hat{x}}_{{ij}}^{t}$, which is independent for each gene given ${\hat{f}}_{j}^{t}$, optimization of ELBO is done separately in parallel for each gene *j* for computational efficiency.

#### Weighing of posterior cell type-specific gene expression profiles

Statescope infers the two variational parameters for ${\hat{x}}_{{ij}}^{t}$, the cell-type-specific gene expression level $({\nu }_{{ij}}^{t})$ and the cell-type-specific gene expression variability $({\omega }_{j}^{t})$. For each cell type and gene, posterior estimate of ${w}_{j}^{t}$ reflects the standard deviation of cell type-specific gene expression in the scRNA-seq reference as well as inter-sample variability observed in bulk RNA-seq data, which can serve as an importance measure for each gene in cell state analysis. Therefore, a weighted cell type-specific gene expression matrix, ${{\boldsymbol{X}}}^{{\boldsymbol{t}}}$, for which its element ${X}_{{ij}}^{t}$ is obtained by centering by ${v}_{{ij}}^{t}$ and weighing by ${w}_{j}^{t}$ as follows:

11 $${X}_{ij}^{t}={\omega }_{j}^{t}({\nu }_{ij}^{t}-{\underline{v}}_{i}^{t})$$

The resulting matrix ${\boldsymbol{X}}$ is mixed-sign and is effectively weighed to capture cell type-specific biological variation.

### Statescope state discovery module

#### Unsupervised state discovery from weighted cell type-specific gene expression profiles

An unsupervised clustering module is introduced to identify biological patterns in the high-dimensional cell type-specific gene expression matrix, ${{\boldsymbol{X}}}^{{\boldsymbol{t}}}$, termed cell states. To this aim, a variation of the non-negative matrix factorization (NMF) algorithm, convex-NMF (cNMF)^15^, is employed. Unlike the standard NMF used in other state analysis methods like Ecotyper^10^, cNMF does not require a data transformation to make the input data non-negative, as it accepts mixed-sign data to achieve an inherently sparse matrix factorization, which improves the interpretability of cell states. For each cell type *t*, the cNMF decomposes the *J* (number of genes) by *I* (number of samples) matrix ${{\boldsymbol{X}}}^{{\boldsymbol{t}}}$ into cell state loadings, the *J* by *K* (number of state) matrix ${{\boldsymbol{F}}}^{{\boldsymbol{t}}}$, and cell state scores, *K* by *I* matrix ${{\boldsymbol{H}}}^{{\boldsymbol{t}}}$, according to the following formulation:

12 $${{\boldsymbol{X}}}^{{\boldsymbol{t}}}\approx {{{\boldsymbol{F}}}^{{\boldsymbol{t}}}{\boldsymbol{H}}}^{{\boldsymbol{t}}}\approx {{\boldsymbol{X}}}^{{\boldsymbol{t}}}{{{\boldsymbol{W}}}^{{\boldsymbol{t}}}{\boldsymbol{H}}}^{{\boldsymbol{t}}}$$

In the convex-NMF algorithm, ${{\boldsymbol{F}}}^{{\boldsymbol{t}}}$ is constrained to be a non-negative linear combination of input matrix ${{\boldsymbol{X}}}^{{\boldsymbol{t}}}$ by introducing the non-negative coefficient matrix ${{\boldsymbol{W}}}^{{\boldsymbol{t}}}$ (*I* by *K* matrix; i.e., ${{\boldsymbol{F}}}^{{\boldsymbol{t}}}={{\boldsymbol{X}}}^{{\boldsymbol{t}}} {{\boldsymbol{W}}}^{{\boldsymbol{t}}}$). This formulation allows a mixed-sign input while keeping all the parameters non-negative. At the start, ${{\boldsymbol{W}}}^{{\boldsymbol{t}}}$ is initialized by randomly selecting *k* samples, and ${{\boldsymbol{H}}}^{{\boldsymbol{t}}}$ is initialized by calculating *k* cluster centroids using the k-means algorithm. Next, ${{\boldsymbol{H}}}^{{\boldsymbol{t}}}$ and ${{\boldsymbol{W}}}^{{\boldsymbol{t}}}$ are iteratively updated as follows:

13 $${H}_{{ki}}^{t}\leftarrow {H}_{{ki}}^{t}\sqrt{\frac{{[{({{{\boldsymbol{X}}}^{{\boldsymbol{t}}}}^{T}{{\boldsymbol{X}}}^{{\boldsymbol{t}}})}\!{\scriptstyle{+}\atop} {{\boldsymbol{W}}}^{{\boldsymbol{t}}}]}_{{ki}}+{[{{\boldsymbol{H}}}^{{\boldsymbol{t}}}{{{\boldsymbol{W}}}^{{\boldsymbol{t}}}}^{T}{({{{\boldsymbol{X}}}^{{\boldsymbol{t}}}}^{T}{{\boldsymbol{X}}}^{{\boldsymbol{t}}})}\!{\scriptstyle{-}\atop}{{\boldsymbol{W}}}^{{\boldsymbol{t}}}]}_{{ki}}}{{[{({{{\boldsymbol{X}}}^{{\boldsymbol{t}}}}^{T}{{\boldsymbol{X}}}^{{\boldsymbol{t}}})}\!{\scriptstyle{-}\atop}{{\boldsymbol{W}}}^{{\boldsymbol{t}}}]}_{{ki}}+{[{{\boldsymbol{H}}}^{{\boldsymbol{t}}}{{{\boldsymbol{W}}}^{{\boldsymbol{t}}}}^{T}{({{{\boldsymbol{X}}}^{{\boldsymbol{t}}}}^{T}{{\boldsymbol{X}}}^{{\boldsymbol{t}}})}\!{\scriptstyle{+}\atop}{{\boldsymbol{W}}}^{{\boldsymbol{t}}}]}_{{ki}}}}$$

14 $${W}_{{jk}}^{t}\leftarrow {W}_{{jk}}^{t}\sqrt{\frac{{[{({{{\boldsymbol{X}}}^{{\boldsymbol{t}}}}^{T}{{\boldsymbol{X}}}^{{\boldsymbol{t}}})}\!{\scriptstyle{+}\atop}{{\boldsymbol{H}}}^{{\boldsymbol{t}}}]}_{{jk}}+{[{({{{\boldsymbol{X}}}^{{\boldsymbol{t}}}}^{T}{{\boldsymbol{X}}}^{{\boldsymbol{t}}})}\!{\scriptstyle{-}\atop}{{\boldsymbol{W}}}^{{\boldsymbol{t}}}{{{\boldsymbol{H}}}^{{\boldsymbol{t}}}}^{T}{{\boldsymbol{H}}}^{{\boldsymbol{t}}}]}_{{jk}}}{{[{({{{\boldsymbol{X}}}^{{\boldsymbol{t}}}}^{T}{{\boldsymbol{X}}}^{{\boldsymbol{t}}})}\!{\scriptstyle{-}\atop}{{\boldsymbol{H}}}^{{\boldsymbol{t}}}]}_{{jk}}+{[{({{{\boldsymbol{X}}}^{{\boldsymbol{t}}}}^{T}{{\boldsymbol{X}}}^{{\boldsymbol{t}}})}\!{\scriptstyle{+}\atop}{{\boldsymbol{W}}}^{{\boldsymbol{t}}}{{{\boldsymbol{H}}}^{{\boldsymbol{t}}}}^{T}{{\boldsymbol{H}}}^{{\boldsymbol{t}}}]}_{{jk}}}}$$

Where ${({{{\boldsymbol{X}}}^{{\boldsymbol{t}}}}^{T}{{\boldsymbol{X}}}^{{\boldsymbol{t}}})}^{+}$ and ${({{{\boldsymbol{X}}}^{{\boldsymbol{t}}}}^{T}{{\boldsymbol{X}}}^{{\boldsymbol{t}}})}^{-}$ concern the non-negative and negative components of ${{{\boldsymbol{X}}}^{{\boldsymbol{t}}}}^{T}{{\boldsymbol{X}}}^{{\boldsymbol{t}}}$, respectively. The optimization is performed until the maximum number of iterations is reached (default = 1000) or convergence as measured by the Frobenius norm. After the optimization the state loadings, ${{\boldsymbol{F}}}^{{\boldsymbol{t}}}$, are computed by ${{\boldsymbol{F}}}^{{\boldsymbol{t}}}={{\boldsymbol{X}}}^{{\boldsymbol{t}}}{{\boldsymbol{W}}}^{{\boldsymbol{t}}}$.

#### Determining the number of cell states

A previously established heuristic algorithm^10^ based on the cophenetic coefficient was used to determine the optimal *k* for each cell type (i.e., the number of cell states). Here, different values of *k* ranging between 2–10 are used for 10 random cNMF initializations. For each *k*, the connectivity matrix between the 10 runs is calculated, followed by the cophenetic coefficient. Based on the heuristic algorithm by Luca et al.^10^, the most stable value for *k* is selected as the optimal number of cell states. Here, a minimal value of the cophenetic coefficient is used as a parameter to determine the stable clustering solutions (default=0.9). The dynamics of the cophenetic coefficients and our final choice of *k* for each cell type are reported in Supplementary Figs. 11 and 12 for the NSCLC and the PDAC applications, respectively. In the PDAC application, we deviated from the chosen *k* in two instances: malignant, where we chose k = 4 instead of k = 3 and CAFs where we chose *k* = 5 instead of *k* = 3. This was done after inspection of the states retrieved in the scRNA-seq data, in which these values of *k* gave a clearer split of known states. After a value for *k* chosen, a final convex-NMF run is performed with 100 random initializations and the optimal value for *k*, where the model with the lowest error according to the Frobenius norm is selected.

#### Retrieving cell states in external datasets

After state discovery, the state loadings ${{\boldsymbol{F}}}^{{\boldsymbol{t}}}$ can be used to assign state scores to samples in weighted cell type-specific gene expression matrices from external datasets $({{{\boldsymbol{X}}}^{{\boldsymbol{*}}}}^{{\boldsymbol{t}}})$. To this end, the state loadings from state discovery $({{\boldsymbol{F}}}^{{\boldsymbol{t}}})$ are fixed and only state scores $({{{\boldsymbol{H}}}^{{\boldsymbol{*}}}}^{{\boldsymbol{t}}})$ are inferred according to the same procedures as described above:

15 $${{{\boldsymbol{X}}}^{*}}^{{\boldsymbol{t}}}\approx {{\boldsymbol{F}}}^{{\boldsymbol{t}}}{{{\boldsymbol{H}}}^{*}}^{{\boldsymbol{t}}}$$

### Bulk and single-cell RNA sequencing datasets used in this study

#### PBMC datasets

For the evaluation and application of Statescope, several bulk and single-cell RNA sequencing datasets are used (overview and accession codes in Supplementary Table 1). We established a bulk RNA sequencing dataset with matched CyTOF for 19 cell types. To this end, we sourced extra cryopreserved vials of 48 PBMCs with available CyTOF data from the previous study on peripheral blood in brain tumor patients collected between April 2015 and November 2018^49^. Since they are only used for benchmarking, patient information such as gender/sex was not collected. The PBMC samples were subject to sorting live cells to best match with CyTOF data, and then to RNA-seq analysis using the KAPA mRNA HyperPrep Kit and Novaseq 6000. Two samples with a large proportion of unknown cell type fractions (16% and 50%) are excluded (gold standard dataset 1; GSE263756, See Supplementary Note 3 for details). Moreover, paired bulk RNA sequencing data of PBMC and FACS-sorted T cell samples and matched CyTOF from 8 individuals from Hoek et al.^17^ (GSE64655) are obtained to evaluate cell type-specific gene expression profile estimation performance (gold standard dataset 2). A scRNA-seq dataset consisting of 605044 phenotyped cells from 143 PBMC samples from Stephenson et al.^24^ (E-MTAB-10026) is obtained to create scRNA-seq signatures for deconvolution.

#### PDAC datasets

Bulk RNA-seq and DNA copy number aberration data of 166 samples from the TCGA-PAAD dataset^35^ are downloaded from the Genomic Data Commons (GDC). A scRNA-seq dataset consisting of 139.446 phenotyped cells from 27 PDAC samples from Werba et al.^36^ (GSE205013) is obtained to create scRNA-seq signatures for deconvolution.

#### NSCLC datasets

Bulk RNA sequencing and DNA copy number aberration data of 1005 NSCLC samples from the TCGA-LUAD^26^ (*n* = 512) and TCGA-LUSC^27^ (*n* = 493) datasets are downloaded from the GDC. Moreover, additional bulk RNA sequencing and tumor purity data from 119 NSCLC intrapulmonary metastasis samples are obtained from the clinically annotated HMF-NSCLC dataset^42^. Another set of bulk RNA sequencing data for 891 primary NSCLC samples from the POPLAR/OAK trials^13^ is obtained from EGA (EGAS00001005013) for state retrieval and biomarker discovery. The POPLAR/OAK trials (POPLAR: NCT01903993, OAK: NCT02008227)^11,12^ are phase 2/3, two-arm randomized controlled trials of previously treated advanced stage NSCLC where patients received either chemotherapy (*n* = 452; docetaxel) or immunotherapy (*n* = 439; PD-L1 inhibitor atezolizumab). An integrated scRNA-seq atlas consisting of 1,283,972 cells from 318 lung samples across 29 datasets from Salcher et al.^3^ is downloaded from the Zenodo repository. Cells without an assigned phenotype (*n* = 19,784) and those derived from material other than the primary tumor (*n* = 690,240) are removed, resulting in a selection of 573,948 phenotyped cells from 176 NSCLC samples across 22 datasets that were used to create scRNA-seq signatures for deconvolution. This phenotyped scRNA-seq dataset is also used to evaluate fraction estimation and cell type-specific gene expression performance by selecting all cells derived from the Zilionis et al.^25^, dataset to create scRNA-seq signatures and creating pseudobulk mixtures from eight separate scRNA-seq datasets (gold standard dataset 3; derived from the Kim et al.^18^, Lambrechts et al.^19^, Leader et al.^21^, Maynard et al.^22^, UKIM-V/UKIM-V2 from Salcher et al.^3^, and Wu et al.^23^).

### Signature creation and deconvolution

#### Creation of scRNA-seq signatures

To create scRNA-seq signatures for Statescope, the expected expression level $({\mu }_{j}^{t})$ and gene expression variability $({\lambda }_{j}^{t})$ for each gene *j* in cell type *t*. ${\mu }_{j}^{t}$ is obtained as the average expression of gene *j* in cell type *t* in the scRNA-seq data. ${\lambda }_{j}^{t}$ is estimated by fitting a LOWESS curve to the mean-variance trend measured in the scRNA-seq data using the fitTrendVar function implemented in scran(v1.34.0)^50^. To exclude zero values in ${\lambda }_{j}^{t}$, a pseudocount of 0.001 is added. For the deconvolution module, cell type-specific marker genes are identified using AutoGeneS (v1.0.4) with default settings^43^. In the evaluation analysis, only the top 3000 highly variable genes according to the CellRanger method^51^ are considered. In state discovery and retrieval analyses, all available genes were used (Supplementary Table 2).

#### Generation of NSCLC pseudobulk mixtures

To evaluate both fraction- and cell type-specific gene expression estimation performance, pseudobulk mixtures are in silico generated from the NSCLC scRNA-seq atlas^3^. Two different strategies were employed to create pseudobulk mixtures: (I) creating patient-specific pseudobulk mixtures (Related to Fig. 1; gold-standard dataset 3) and (II) creating state-specific pseudobulk mixtures (Related to Fig. 2). Patient-specific pseudobulk mixtures are created by calculating the cumulative sum of raw counts from all cells per patient sample in the pseudobulk datasets (Supplementary Table 1). Patient-specific pseudobulk mixtures are generated to evaluate the performance of fraction- and cell type-specific gene expression estimation.

State-specific pseudobulk mixtures are created by randomly sampling cells from a distinct set of cell states. To this aim, the log, library-size-corrected counts were selected to perform scaling and principal component analysis (PCA), followed by computing the K-nearest neighbors (KNN) graph using Scanpy (v1.7.0)^52^ with default settings. Using the KNN graph, unsupervised clustering analysis using Leiden graph-based clustering^53^ is applied to cluster individual cells into cell states using Scanpy, with the resolution set to 0.5. This procedure is performed separately for each cell type and each pseudobulk dataset. For each dataset, 50 different combinations of distinct cell states are sampled without replacement, and to generate 50 accompanying sets of cell type compositions by randomly sampling from a Dirichlet distribution with a strong informative prior *D*(ɑ), with ɑ = 100(*f*_1_…*f*_*t*_) where *f*_*t*_ denotes the fraction of cell type t in the scRNA-seq dataset. To create 50 state-specific pseudobulk mixtures, a total of 1000 cells are sampled without replacement from distinct cell states that matched the predefined cell type composition. State-specific pseudobulk mixtures are generated to evaluate the performance of state-specific gene expression estimation.

#### Evaluation of (pseudo)bulk transcriptome deconvolution

For benchmarking using the PBMC dataset with CyTOF, deconvolution is performed using signatures with 19 cell types and also 10 cell types with the T cell lineage aggregated (Supplementary Table 2). In these two experiments, prior knowledge of T cell fractions determined by CyTOF, either as a group or as a single prior, is given to Statescope. To evaluate the fraction estimation performance, the PCC and RMSE between the predicted fractions and CyTOF fractions for each sample are calculated. In addition, to evaluate the robustness of Statescope to imprecise prior fractions, the following three levels of Gaussian noise are imposed (low: N(0,0.1), intermediate N(0,0.15), high N(0,0.5)) to the prior T cell fractions. For benchmarking using the PBMC dataset with FACS sorted T cells^17^, scRNA-seq signatures for 10 cell types and a prior T cell fraction determined by the CyTOF is used for deconvolution. CIBERSORTx could not be included in this evaluation due to the limited sample size. To evaluate the cell type-specific gene expression performance, the PCC between the predicted T cell expression and FACS sorted T cell expression over all samples are calculated.

For benchmarking using the NSCLC pseudobulk mixtures derived from the 8 separate NSCLC datasets^3,18–23^, a scRNA-seq signature with 15 cell types derived from the Zillionis et al. dataset^25^ is used (Supplementary Table 1-2). The true malignant cell fraction in the pseudobulk mixtures is used as a single prior for deconvolution with Statescope. For the patient-specific pseudobulk mixtures, the PCC and RMSE between predicted fractions and true fractions are calculated per sample to evaluate the fraction estimation performance. For both the patient- and state-specific pseudobulk mixtures, the PCC between the true and predicted cell-type-specific gene expression is calculated per sample and cell type to evaluate the performance of cell-type-specific gene expression estimation. In the patient-specific pseudobulk mixtures, CIBERSORTx was only able to estimate cell type-specific gene expression in the Wu et al. dataset^23^ due to limited sample sizes. In the state-specific pseudobulk mixtures, the percentage of correctly recovered states is calculated as a performance measure. This analysis is performed using a supervised approach for all methods by projecting the predicted cell type-specific gene expression profiles onto the scRNA-seq data for each cell type and pseudobulk dataset using *scanpy.tl.ingest*^52^ and transferring the cell state labels. Next, the percentage of correctly mapped states is calculated for each cell type and pseudobulk dataset. For Statescope, an unsupervised state discovery using cNMF is also applied and evaluated. The number of states for cNMF is set to the number of states sampled from in the cell type, and for each sample, the state with the highest state score is assigned as the inferred state. Next, the percentage of correctly assigned states is calculated for each cell type and pseudobulk dataset.

In all Statescope applications, the default hyperparameter configuration is applied: ${\alpha }_{i}^{t}=1$, ${\alpha }_{0j}^{t}=1000$, ${\kappa }_{0j}^{t}=$1, s=1 and 100 optimization iterations. These hyperparameters are uninformative priors, except for ${\alpha }_{0j}^{t}=1000$ that puts a high confidence on observed gene expression variability in single-cell RNAseq data. These default configurations allow us to omit the time-consuming hyperparameter optimization based on the previous Empirical Bayes framework. The BayesPrism R-package (2.0)^5^ receives raw counts as input following the documentation with default settings. BayesPrism input cell states are identified by reclustering of cells within each cell type using Leiden graph-based clustering^53^ with default settings. Cell states with less than 20 cells are assigned to an unknown cell state as described in the documentation. The TAPE python package (v1.1.2)^7^ is implemented using raw counts as input according to the documentation with default settings. The Docker version of CIBERSORTx (v1.0)^4^ is used with library-size corrected linear counts as input according to the documentation, with default settings. In fraction estimation evaluation experiments, only marker genes selected by AutoGeneS^43^ are used. In cell type-specific gene expression estimation evaluation experiments, the top 3000 highly variable genes according to the CellRanger method^51^ are considered.

#### Application of Statescope to TCGA, HMF and POPLAR/OAK bulk RNA datasets

Raw bulk RNA counts were retrieved for the TCGA-NSCLC^26,27^, TCGA-PAAD^35^, HMF-NSCLC^42^ and POLAR/OAK datasets^13^. Raw counts were total count normalized to 10,000 reads per sample and subjected to Statescope. For the TCGA datasets, copy number aberration segments were retrieved and ACE (1.17.0) is used to predetermine malignant cell fractions by fitting a range of purities (0.05–1.0) and ploidies (1–5)^28^. The purity of the fit with the lowest RMSE is selected as prior knowledge of the malignant cell fraction. Samples with a purity of 1.0 determined by ACE are excluded (TCGA-NSLC: *n* = 36, TCGA-PAAD: *n* = 12) since these are samples with no copy number aberrations and therefore unreliable, as described by Poell et al.^28^. In the HMF application, whole-genome sequencing-based tumor purity as presented by HMF^42^ is used as prior knowledge. Prior knowledge of the malignant fraction was not available and therefore not used for the POPLAR/OAK dataset. For the application of Statescope to TCGA-PAAD, a scRNA-seq signature was created using the phenotyped scRNA-seq data by Werba et al. (*n* = 7 cell types)^36^. For CAFs, subtype labels were available to the authors for validation of the CAF states discovered by Statescope. A second scRNA-seq signature was created using the tumor basal-like, intermediate and classical subtypes, which were determined using the gene set scoring method from Raghavan et al^37^. The PurIST classifier^40^ was applied to the raw bulk RNA counts of TCGA-PAAD, to classify the samples into classical and basal-like. This classification was used for further validation of the malignant cell states. For the NSCLC application, a scRNA-seq signature was created using the phenotyped scRNA-seq atlas integrated by Salcher et al.^3^ (*n* = 15 cell types; see Supplementary Table 2).

State discovery was performed for both the TCGA-NSCLC and TCGA-PAAD datasets. The weighted cell type-specific gene expression matrices were concatenated, and PCA, KNN graph and Uniform Manifold Approximation and Projection (UMAP) embeddings^54^ were calculated to create TCGA bulk atlases. The TCGA state loadings were used to retrieve state scores in external datasets. State retrieval in phenotyped single cell datasets (PDAC: Werba et al.^36^, NSCLC: Salcher et al.^3^, and Hanley et al.^34^) was performed by mean centering of scRNA-seq counts followed by multiplication with the gene expression standard deviation measured from the scRNA-seq data. State retrieval in bulk RNA sequencing datasets (HMF, POPLAR/OAK) was performed by using cell-type-specific gene expression profiles weighted by gene importance. For visualization of state scores in the Salcher et al. scRNA-seq data, UMAP representations were recomputed after making cell type selections using the scANVI embeddings^55^ established by the original authors^3^.

#### Statistics & reproducibility

To evaluate statistical significance between continuous variables, a one-sided, paired Wilcoxon rank-sum test is used unless otherwise specified. A P-value threshold of 0.05 is used to define significance, where more significant P-values under 0.005 and 0.0005 are also indicated by asterisks. The prognostic value of continuous state scores is evaluated by univariate analysis of 4-year OS using Cox regression. The predictive value of state scores is evaluated by multivariate analysis of 4-year OS using Cox regression with the treatment arm in the POPLAR/OAK cohort as the interaction term. For Kaplan-Meier analysis, states are dichotomized into ‘high’ and ‘Other’ by setting a threshold at the 75th percentile of state scores. Global Kaplan-Meier P-values are determined using the log-rank test. All survival analyses are based solely on cell states identified from RNA-seq data, without accounting for other patient information such as sex and gender.

### Reporting summary

Further information on research design is available in the Nature Portfolio Reporting Summary linked to this article.

## Supplementary information

Supplementary Information

Reporting Summary

Transparent Peer Review file

## Source data

Source Data

## Data Availability

A complete overview of datasets and accession codes are available in Supplementary Table 1. Processed scRNA-seq datasets are publicly available: Stephenson et al.^24^ [https://www.ebi.ac.uk/biostudies/arrayexpress/studies/E-MTAB-10026/files], Salcher et al.^3^ [https://zenodo.org/records/7227571] and Werba et al.^36^ [https://www.ncbi.nlm.nih.gov/geo/query/acc.cgi?acc=GSE205013]. Raw and processed bulk RNA-seq data from the PBMC samples with matched CyTOF are deposited in the NCBI Gene Expression Omnibus (GEO) under accession code GSE263756. Bulk RNA sequencing data from Hoek et al. are deposited on GEO under accession code GSE64655. Access to bulk RNA and clinical data from the HMF cohort can be requested through https://www.hartwigmedicalfoundation.nl/en/data/data-access-request/ and is granted upon evaluation of the HMF Data Access Board. Access to the bulk RNA and clinical data from the POPLAR/OAK cohort can be requested through the European Genome-Phenome Archive database under accession code EGAD00001007703. TCGA copy number aberration-, RNA- and clinical data were downloaded from the GDC data portal [https://portal.gdc.cancer.gov]. The TCGA cell state atlas results can be interacted with and found on the R2 platform upon registration [http://r2platform.com/statescope/]. Source data are provided with this paper. This includes the state loadings of the NSCLC and PDAC TCGA state discovery, enabling users to retrieve these states in their data. Source data are provided with this paper.

## Code availability

A dedicated webpage for Statescope is available at https://tgac-vumc.github.io/Statescopeweb/ which includes a Python package, a Bioconductor R package (10.18129/B9.bioc.StatescopeR) and user-friendly documentation. Source code, released under a MIT-license, is hosted on Github at https://github.com/tgac-vumc/Statescope and in Zenodo [https://zenodo.org/records/19557998]. Scripts to run ACE from copy number aberration segments of the TCGA datasets can be found at https://github.com/tgac-vumc/TCGA_purity. Other custom code is part of the advanced tutorial of the Python package.
