# Supplementary material for: Statescope: an integrative deconvolution framework for discovering cell states in tumors
Source: Nat Commun. 2026 Jul 6;17:8465. doi: 10.1038/s41467-026-74997-8 (PMC13478200; doi:10.1038/s41467-026-74997-8)
Supplement: Supplementary file 1 [file 41467_2026_74997_MOESM1_ESM.pdf]

Supplementary Material

Supplementary Tables

Supplementary Table 1: Overview of datasets used in this study

Dataset	Dataset name	Platform	Accession	Number of samples analyzed	Number of cells analyzed	Number of cell types analyzed	Figure
**PBMC**							
scRNA-seq	Stephenson et al (1)	10X	E-MTAB-10026	143	605044	9, 19	1c-f, 1i
Bulk RNA (I)	PBMCs + CyTOF (2)		GSE263756	46		9, 19	1c-f
Bulk RNA (II)	Hoek et al (3) (PBMCs + FACS)	HiSeq2000	GSE64655	8		9	1i
**NSCLC**							
scRNA-seq atlas	Salcher et al (4)		Zenodo	254	573948	15	3, 5
scRNA-seq signature	Zilionis et al (5)	InDrop	Zenodo	18	25386	15	1g-h, 1j-k, 2
scRNA-seq pseudobulk (I)	Kim et al (6)	10X	Zenodo	15	47211	15	1g-h, 1j-k, 2
scRNA-seq pseudobulk (II)	Lambrechts et al (7)	10X	Zenodo	3	28552	15	1g-h, 1j-k, 2
scRNA-seq pseudobulk (III)	Laughney et al (8)	10X	Zenodo	8	12362	15	1g-h, 1j-k, 2
scRNA-seq pseudobulk (IV)	Leader et al (9)	10X	Zenodo	21	61521	15	1g-h, 1j-k, 2
scRNA-seq pseudobulk (V)	Maynard et al (10)	Smart-seq	Zenodo	8	6941	15	1g-h, 1j-k, 2
scRNA-seq pseudobulk (VI)	Salcher et al (4) (UKIM-V)	BD-Rhapsody	Zenodo	3	11269	15	1g-h, 1j-k, 2
scRNA-seq pseudobulk (VII)	Salcher et al (4) (UKIM-V2)	BD-Rhapsody	Zenodo	14	53580	15	1g-h, 1j-k, 2
scRNA-seq pseudobulk (VIII)	Wu et al (11)	Singleron	Zenodo	42	57472	15	1g-h, 1j-k, 2
Bulk DNA/RNA TCGA	TCGA-NSCLC (12, 13)		GDC	973		15	3, 5
Bulk RNA HMF	HMF (14)		HMF	119		15	5a
Bulk RNA POPLAR/OAK	POPLAR/OAK (15)		EGAD00001007703	891		15	5
**PDAC**							
scRNA-seq	Werba et al (16)	10X	GSE205013	27	139446	7, 9	4
Bulk DNA/RNA TCGA	TCGA-PAAD (17)		GDC	166		7, 9	4

Supplementary Table 2: Characteristics of scRNA-seq signatures used in this study

Signature	Celltypes	N	Prior given	Number of marker genes identified	Number of genes analyzed	Figure
**PBMC**						
Signature (I)	**T cells**, B cells, Monocytes non-classical, NK cells, B cells memory, Late NK cells, Early NK cells, Monocytes classical, ILCs	9	**T cell**	485	3000	1c-d, i
Signature (II)	**CD4T eff. mem.**, **CD4T central. mem.**, **CD4T naïve**, **CD4T eff.**, **CD8T eff. mem.**, **CD8T central. mem.**, **CD8T naïve**, **CD8T eff.**, **CD4-CD8-T cells**, **Treg**, B cells, Monocytes non-classical, NK cells, B cells memory, Late NK cells, Early NK cells, Monocytes classical, ILCs	19	**T cell group**	459	3000	1e-f
**NSCLC**						
scRNA-seq evaluation signature	**Malignant cell**, CD8 T cell, B cell, Plasma cell, Regulatory T cell, Mast cell, CD4 T cell, Macrophage, Alveolar cell, NK cell, Monocyte, Neutrophil, Dendritic cell, Endothelial cell	15	**Malignant cell**	742	3000	1g-h, 1j-k, 2
scRNA-seq atlas signature				613	17007	3, 5
**PDAC**						
'Simple' signature	**Malignant cell**, B cell, Fibroblast, Endothelial cell, Mast cell, Myeloid cell, T cell	7	**Malignant cell**	626	21635	4
'Detailed' signature	**Basal-like cell**, **Intermediate cell**, **Classical cell**, B cell, Fibroblast, Endothelial cell, Mast cell, Myeloid cell, T cell	9	**Malignant cell group**	638	21635	4f-g

Supplementary Table 3: Annotation of NSCLC cell states

Cell state	Given name	Top 5 State loadings	Salcher et al annotation	References
**Malignant cell**				
State 1	**LUAD-EGFR-1**	CEACAM6, S100A6, KRT7, HLA-DRA, HLA-DRB1	No enrichment	
State 2	**LUAD-EGFR-2**	CEACAM6, IGHG1, CD74, HLA-DRA, C3	No enrichment	
State 3	**LUAD-STK11**	AKR1C1, AKR1C2, FGG, FGA, MSLN	No enrichment	
State 4	**NSCLC-NOS**	KIF1A, PEG10, STMN1, TOP2A, CALCA	No enrichment	
State 5	**LUSC**	KRT5, KRT6A, KRT17, KRT14, PKP1	No enrichment	
**Alveolar cell**				
State 1	**AT1/2-1**	SFTPB, SLC34A2, PIGR, NAPSA, SFTPA2	AT1/AT2/Club	Salcher et al (4)
State 2	**AT2**	SFTPB, SFTPA2, PGC, SFTPA1, SCGB3A2	AT2	Salcher et al (4)
State 3	**AT1/2-2**	SFTPA1, SFTPB, SFTPA2, TPT1, NAPSA	AT1/AT2/Club	Salcher et al (4)
State 4	**Ciliated**	GAPDH, S100A11, HSP90AA1, PTMA, RPL35A	Ciliated	Salcher et al (4)
**Endothelial cell**				
State 1	**Capillary/Arterial**	PECAM1, VWF, SPARCL1, ENG, A2M	Capillary/Arterial	Salcher et al (4)
State 2	**Venous/Lymphatic**	GNG11, RPL32, RPS9, VAMP5, RPLP2	Venous/Lymphatic	Salcher et al (4)
State 3	**Venous**	HNRNPA2B1, HSPG2, APP, HSP90AA1, GAPDH	Lymphatic	Salcher et al (4)
**Plasma cell**				
State 1	**Plasma-1**	IGKC, JCHAIN, IGLC2, IGHA1, IGLC3	N.A	
State 2	**Plasma-2**	IGKC, IGHG1, MALAT1, IGHG2, RPS19	N.A	
State 3	**Plasma-3**	B2M, RPLP1, RPL10, RPL41, RPS27	N.A	
State 4	**TLS/Memory**	**SSR4**, **MZB1**, FKBP11, **DERL3**, **CD27**	N.A	Patil et al (15), Meylan et al (18)
State 5	**Plasma-4**	IGKC, B2M, IGHA1, IGHG1, MALAT1	N.A	
**CD4 T cell**				
State 1	**Proliferating**	RPS17, RPL36A, NACA, RPL38, RPL4	N.A	Szabo et al (19)
State 2	**Activated**	CD3D, **CD69**, CD52, TRAC, CD2	N.A	Szabo et al (19)
State 3	**Resting**	**IL7R**, CYTIP, PTPRC, CD37, ZFP36L2	N.A	Szabo et al (19)
**Macrophage**				
State 1	**Infl-TAM-1**	**LYZ**, **MRC1**, HLA-DMA, **HLA-DRA**, **HLA-DQA1**	No enrichment	Ma et al (20)
State 2	**LA-TAM-1**	**APOC1**, C1QA, APOE, TYROBP, SRGN	No enrichment	Ma et al (20)
State 3	**RTM-TAM**	LYZ, C1QB, C1QC, HLA-DQA1, **CCL18**	No enrichment	Ma et al (20)
State 4	**LA-TAM-2**	**APOE**, C1QA, C1QB, CTSD, HLA-DRA	No enrichment	Ma et al (20)
State 5	**Infl-TAM-2**	**S100A11**, RPLP1, SERF2, GAPDH, RPS19	No enrichment	Ma et al (20)
**CD8 T cell**				
State 1	**Effector**	CD3D, **GZMK**, NKG7, **GZMH**, CD2	No enrichment	
State 2	**Exhausted**	HNRNPA2B1, GAPDH, NCL, PRRC2C, FUS	No enrichment	
State 3	**Early activated**	**CD69**, CD52, ITM2A, GMFG, ALOX5AP	No enrichment	
**Dendritic cell**				
State 1	**cDC-type I/II-1**	FCER1G, MS4A6A, SPI1, ALOX5AP, LCP1	cDC1/cDC2	Salcher et al (4)
State 2	**cDC-type I/II-2**	LST1, AIF1, FCER1G, MS4A6A, TYROBP	cDC1/cDC2	Salcher et al (4)

State 3	**pDC**	RPLP1, NACA, HNRNPA1, RPL6, RPS17	pDC/Mature DC	Salcher et al (4)
State 4	**cDC-type II**	GPR183, MS4A6A, AIF1, FCER1G, ALOX5AP	cDC2	Salcher et al (4)
**Fibroblast**				
State 1	**Myofibroblast**	**COL1A1**, COL1A2, FN1, COL12A1, **COL3A1**	Myofibroblast (Hanley)	Hanley et al (21)
State 2	**Adventitial**	IGKC, JCHAIN, IGHA1, IGHM, IGLC2	Adventitial (Hanley)	Hanley et al (21)
State 3	**Alveolar**	RPS17, RPLP1, RPS7, RPLP0, RPL35A	Alveolar (Hanley)	Hanley et al (21)
**NK cell**				
State 1	**Activated**	LCP1, **CD69**, TYROBP, **CD52**, FCER1G	N.A	Rebuffet et al (22)
State 2	**Early**	**FGFBP2**, RPS17, YWHAZ, TMA7, RPS21	N.A	Rebuffet et al (22)
State 3	**Mature**	GZMH, KLRD1, **CD247**, NKG7, PRF1	N.A	Rebuffet et al (22)
**Mast cell**				
State 1	**Mature**	SAMSN1, CD69, **FCER1G**, ALOX5AP, TYROBP	N.A	Wu et al (23)
State 2	**Early**	HNRNPA2B1, DYNLL1, HSP90AB1, HNRNPA1, MALAT1	N.A	Wu et al (23)
State 3	**Activated**	**TPSAB1**, **TPSB2**, MS4A2, **CPA3**, HPGDS	N.A	Wu et al (23)
**Monocyte**				
State 1	**Mono-1**	TYROBP, AIF1, FCER1G, LST1, BCL2A1	No enrichment	
State 2	**Mono-2**	LYZ, FCER1G, CYBB, CD14, LCP1	No enrichment	
State 3	**Proliferating**	RPL6, RPS2, RPLP1, RPL7, RPS28	No enrichment	
**B cell**				
State 1	**Activated-1**	**CD69**, CD52, GPR183, CD37, CYTIP	N.A	Fitzsimons et al (24)
State 2	**Activated-2**	CYTIP, CD37, **CD69**, GPR183, MALAT1	N.A	Fitzsimons et al (24)
State 3	**Naïve**	CD79B, CD37, CD79A, **MS4A1**, **CD19**	N.A	Fitzsimons et al (24)
State 4	**Mature**	**CD37**, CD52, HLA-DQA1, LAPTM5, EVI2B	N.A	Fitzsimons et al (24)
State 5	**Proliferating**	RPS17, COX4I1, RPLP0, RPL36A, PPIA	N.A	Fitzsimons et al (24)
**Regulatory T cell**				
State 1	**Regulatory-1**	CD3D, **TIGIT**, CD2, TRBC2, CD27	N.A	Miragaia et al (25)
State 2	**Resting**	ITM2A, CYTIP, **IL7R**, ETS1, MALAT1	N.A	Miragaia et al (25)
State 3	**Regulatory-2**	**TIGIT**, CYTIP, CD27, TNFRSF4, CD37	N.A	Miragaia et al (25)
State 4	**Effector-1**	**TNFRSF18**, HNRNPA2B1, SOD1, HNRNPA1, GAPDH	N.A	Miragaia et al (25)
State 5	**Effector-2**	ITM2A, **CD52**, **CD69**, GMFG, CREM	N.A	Miragaia et al (25)
**Neutrophil**				
State 1	**TAN**	LCP1, EVI2B, ALOX5AP, FCER1G, IGSF6	TAN	Salcher et al (4)
State 2	**NAN**	C5AR1, FPR1, AQP9, ALOX5AP, BCL2A1	NAN	Salcher et al (4)
State 3	**Intermediate**	ADM, VEGFA, BASP1, IER3, EIF1	NAN/TAN	Salcher et al (4)
State 4	**TAN-2**	LCP1, IL1RN, HCAR3, IL1B, BCL2A1	TAN	Salcher et al (4)

Supplementary Table 4: Annotation of PDAC cell states

Cell state	Given name	Top 5 State loadings	Werba et al. annotation	References
**Malignant cell**				
State 1	**Basal-like**	**KRT17, S100A2**, TGM2, **KRT7**, ANXA1	Basal-like*	Raghavan et al (26)
State 2	**Exocrine**	**PNLIP, CPA1, CPB1**, PRSS1, PRSS2	N.A.	Mofitt et al (27)
State 3	**Neuroendocrine/Int.**	**CHGB**, TTR, **CHGA**, PCSK1N, APLP1	Intermediate*	Weisbrod et al (28)
State 4	**Classical**	**REG4**, **TFF1, CEACAM5**, **CLDN18**, MUC5AC	Classical*	Raghavan et al (26)
**Endothelial cell**				
State 1	**TEC-1**	CALD1, **ITGA6**, HSPG2 , TIMP3, CLIC4	N.A	Kim et al (29)
State 2	**TEC-2**	IDI1, S100A16, S100A11, **TAGLN2**, RHOC	N.A	Jin et al (30)
State 3	**TEC-3**	BCAM, **TSPAN7**, MAP1B, NPDC1, ENPP2	N.A	Wuttig et al (31)
State 4	**Vascular/Normal**	IGFBP4, MGP, ADAMTS1, HLA-DRA, PECAM1	N.A	
**CAF cell**				
State 1	**Pericyte**	CPE, SCG5, PTPRN, SCG2, GPX3	Pericytes	
State 2	**Mixed/myCAF**	**VCAN**, **FN1**, ITGB1, THBS1, **THBS2**	Mixed/myCAF	Kazakova et al (32)
State 3	**iCAF**	**C7, C3**, MGP, COL14A1, CCDC80	iCAF	Helms et al (33)
State 4	**myCAF**	**MMP11**, FN1, COL6A2, LGALS1, MMP14	myCAF	Cords et al (34)
State 5	**Proliferating**	MT-CO2, S100A6, S100A11, MT-CO3, RPL13	Chondrocyte-like, Proliferating, Peri-islet Schwann cells	
**B/Plasma cell**				
State 1	**Proliferating**	TMSB10, **NEAT1**, MT-ND1, MT-CO3, MT-ND2	N.A	Qian et al (35)
State 2	**Plasma**	**XBP1**, SEC11C, ERO1B, SSR4, ATP2A3	N.A	Shaffer et al (36)
State 3	**Antigen presenting**	HLA-DRA, HLA-DPA1, CD74, HLA-DRB1, IGHG1	N.A	
**T/NK cell**				
State 1	**Activated NK**	**CCND2**, TENT5C, GNG2, SSR4, TPST2	N.A	Campbell et al (37)
State 2	**Activated T**	**S100A11**, S100A10, PPDPF, S100A6, FXYD5	N.A	Zhou et al (38)
State 3	**Infiltrating**	**LCP1**, IL7R, LAPTM5, CXCR4, ETS1	N.A	Zeng et al (39)
**Myeloid cell**				
State 1	**General**	HLA-DRA, HLA-DPA1, HLA-DQA1, PTPRC, LCP1	N.A	
State 2	**Activated Mφ**	**TSPO**, GSTP1, PPDPF, ANXA2, CSTB	N.A	Lanfranca et al (40)
State 3	**M1 Mφ**	KCNMA1, MAFB, OTULINL, **RTN1,** KCTD12	N.A	Zhu et al (41)
**Mast cell**				
State 1	**General**	CLU, AZGP1, CADPS, P2RX1, BEX4	N.A	
State 2	**Tumor promoting**	**LAPTM5**, RGS1, PTPRC, LCP1, TNFAIP3	N.A	Sun et al (42)
State 3	**Proliferating**	PPDPF, ATP5F1E, COX4I1, ATP5MC2, GPX4	N.A	

*Malignant cell annotation was performed using the same procedure as used by Werba et al (16)

Supplementary Figures

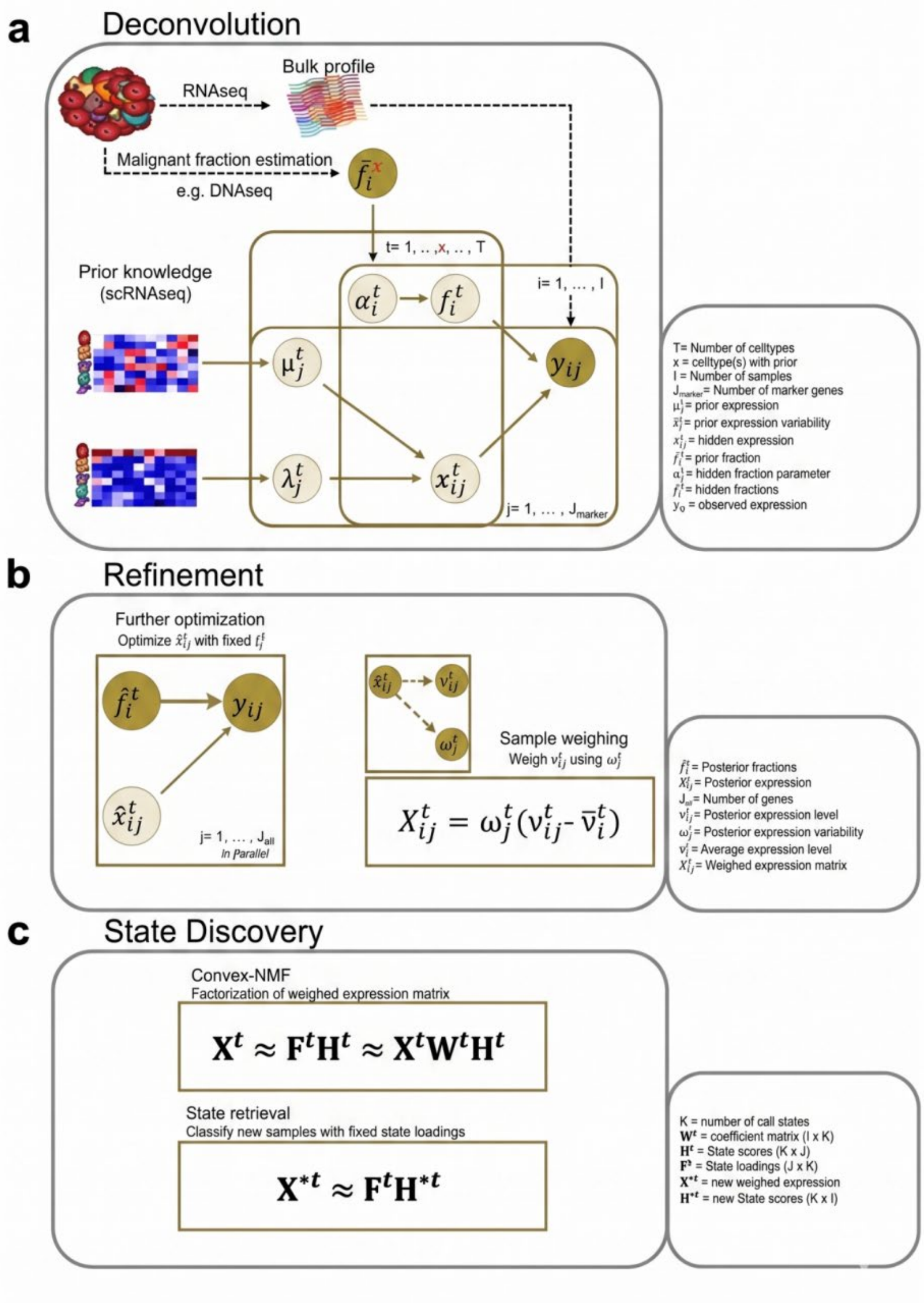

Supplementary Figure 1. Overview of the Statescope framework. The graphical model depicts dependency (solid arrows) between the hidden variables (off-white) and the observed variables (olive-green) in the Statescope deconvolution module (**a**). For each sample i and gene j, the observed bulk gene expression level (y_{ij}) is reconstructed by two hidden variables; the cell fraction (f_i^t) of each cell type t in sample i and the cell type-specific gene expression profiles x_{ij}^t. Statescope uses scRNA-seq signatures composed of the average cell type specific-gene expression level (μ_j^t) and cell type-specific gene expression variability (λ_j^t). Prior knowledge of the expected malignant cell fraction ($\bar{f}_i^x$) is integrated and used to optimize hyperparameter α_i^t. The first deconvolution step is performed using only marker genes (J_{marker}). Overview of the Refinement module in the Statescope framework (**b**). In the refinement module, the posterior estimate of cell fractions ($\hat{f}_i^t$) after the first deconvolution is fixed while further optimizing the estimates of the cell type-specific gene expression profiles ($\hat{x}_{ij}^t$). The posterior estimate of the cell type-specific gene expression profiles ($\hat{x}_{ij}^t$) consists of two parameters, the cell type-specific gene expression level (v_{ij}^t) and the cell type-specific gene expression variability (ω_j^t) which can be used as an gene importance measure. Both parameters are used to produce the weighed cell type-specific gene expression matrices ($\mathbf{X}^t$). Overview of the State Discovery module in the Statescope Framework (**c**). Statescope leverages convex-NMF to decompose the weighed gene expression matrices ($\mathbf{X}^t$) into state scores ($\mathbf{H}^t$) and state loadings ($\mathbf{F}^t$) for each cell type *t*. State scores of new weighed cell type-specific gene expression matrices ($\mathbf{X}^{*t}$, e.g. scRNA-seq data) can be retrieved using predefined state loadings ($\mathbf{F}^t$). For each variable, the sub- and superscripts denote elements as follows: t, celltype; i, sample; j, gene.

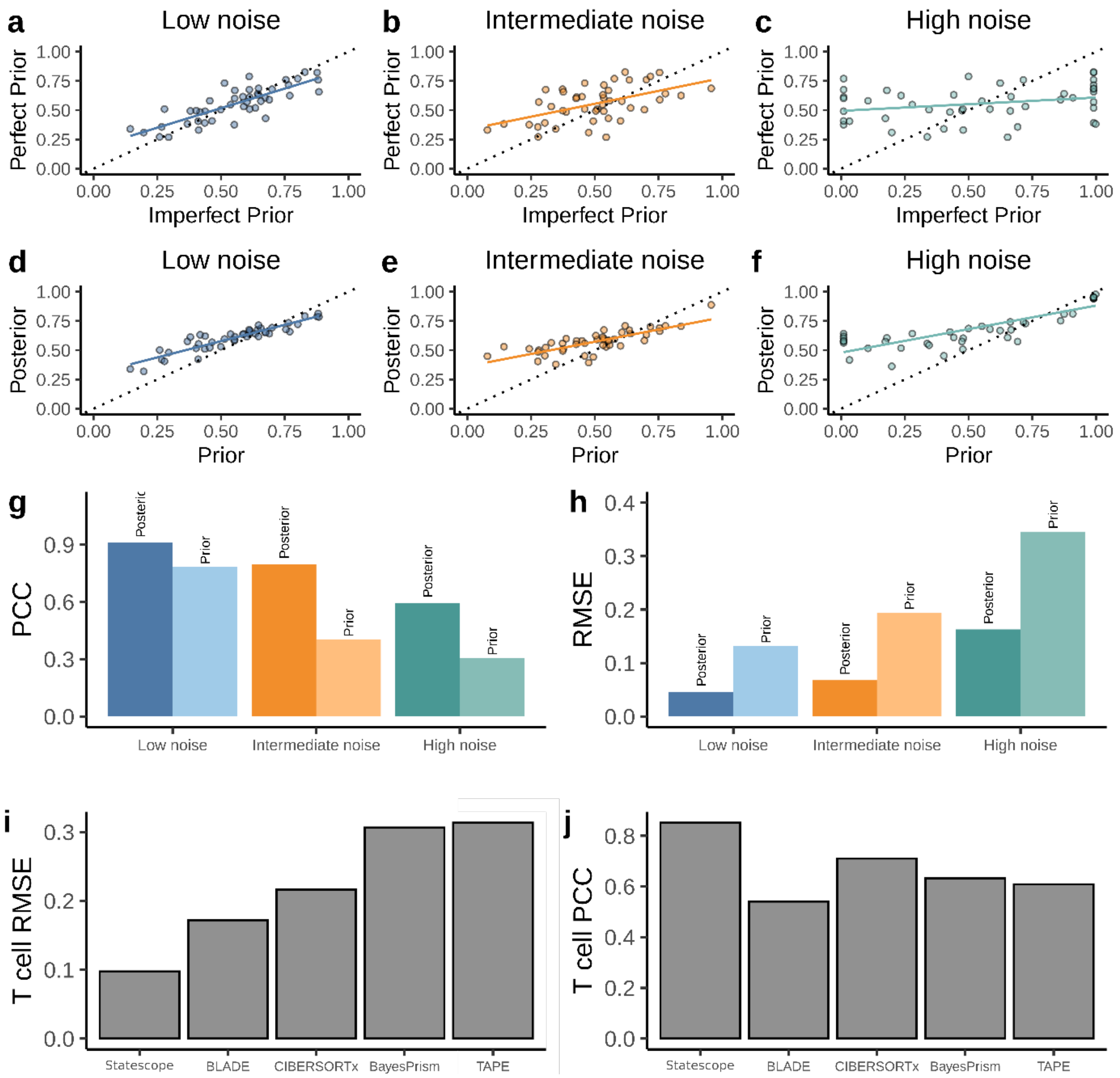

Supplementary Figure 2. Statescope deconvolution with imperfect prior knowledge. Imposing three levels of Gaussian noise: low: N(0,0.1) (**a**), intermediate N(0,0.15) (**b**), high N(0,0.5) (**c**) on prior knowledge of T cell fractions (n=46). The y-axis denotes the perfect prior (true T cell fraction) and the x-axis denotes the prior after imposing noise. The effect of adding low (**d**), intermediate (**e**) and high (**f**) noise to the prior T cell fraction on the posterior outcome of the T cell fraction after Statescope deconvolution. The dotted lines indicate the y=x line and the colored lines indicate the regression line. Pearson correlation coefficient (PCC) (**g**) and Root Mean Square Error (RMSE) (**h**) of posterior and prior T cell fractions compared to the posterior T cell fractions when giving a perfect prior across three levels of noise. Benchmarking of performance in estimating T cell fractions as measured by RMSE (**i**) and PCC (**j**). Source data for supplementary figure 2a-j are provided as a Source Data File.

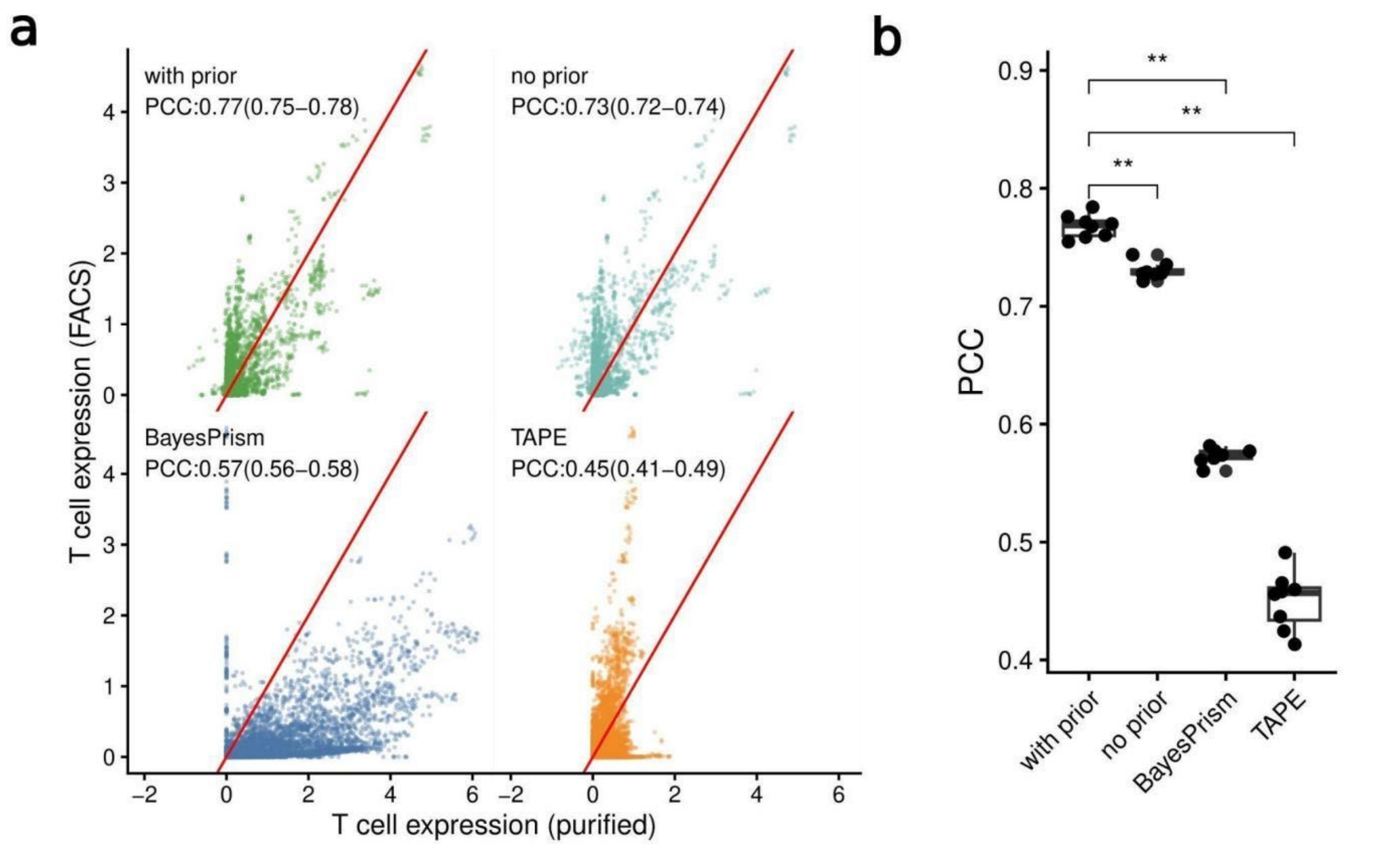

Supplementary Figure 3. Impact of T cell fraction prior on T cell gene expression profiles. Performance of cell type-specific gene expression estimation measured by PCC using predicted-T cell profiles and FACS-sorted T cell profiles in the gold-standard dataset 2 across 8 samples (**a**). Statescope performance is shown with T cell fraction prior (with prior) or without T cell fractions prior (no prior) compared to BayesPrism and TAPE. PCC measuring T cell gene expression estimation performance per sample (**b**). CIBERSORTx could not be applied in these evaluations due to limited sample size. Statistical significance in boxplot comparisons between Statescope with prior (with prior), Statescope with no prior (no prior) and other methods is determined using a one-sided, paired Wilcoxon rank-sum test and indicated as follows: **: p<=0.01. Boxplots show PCC distributions. The centre line indicates the median (50th percentile). The box bounds represent the first and third quartiles (25th and 75th percentiles), defining the interquartile range (IQR). Whiskers extend to the minimum and maximum values within 1.5 × IQR of the quartiles. All samples are shown as dots. Source data are provided as a Source Data File.

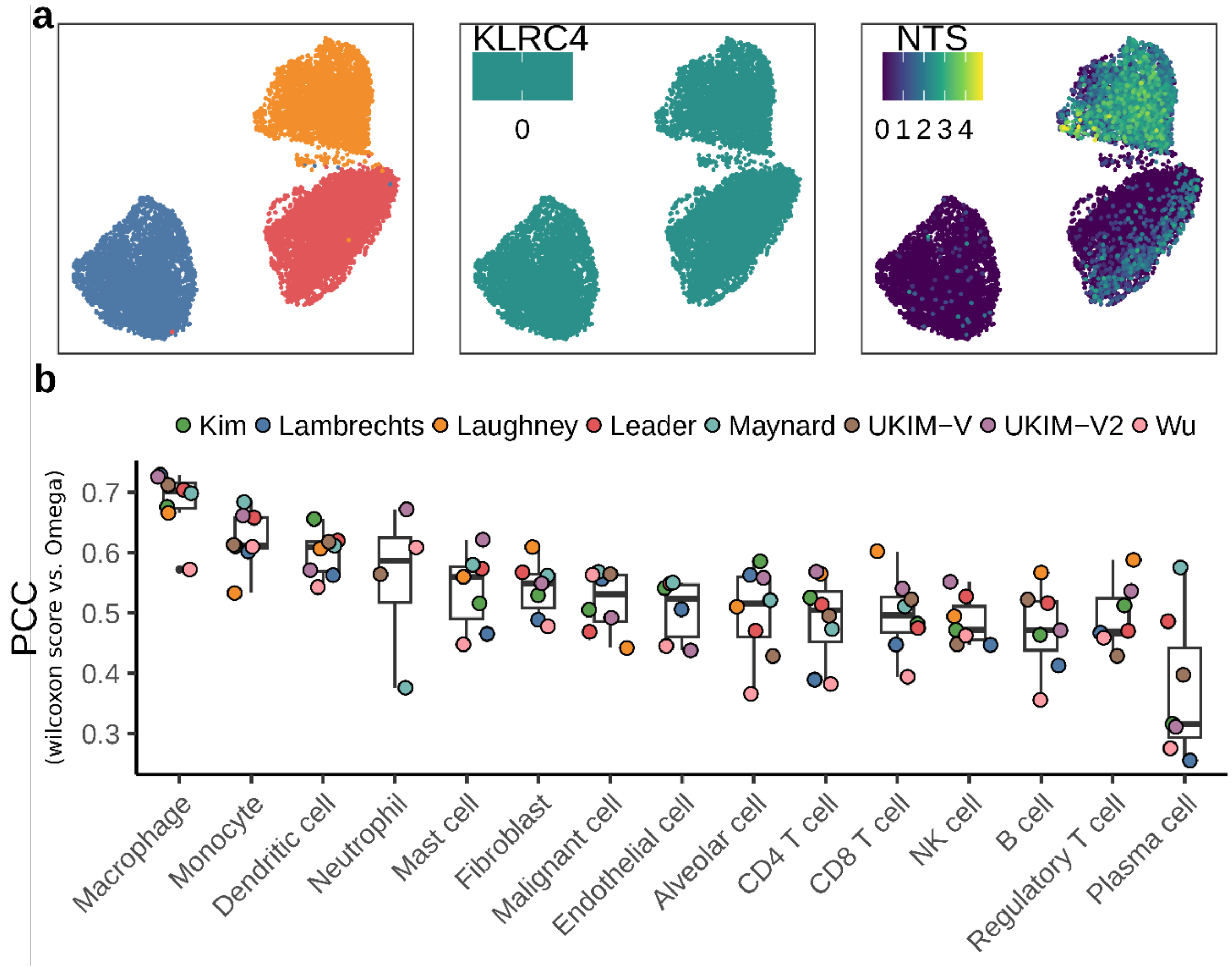

Supplementary Figure 4. Posterior estimates of gene expression variability as a gene importance measure. Difference between genes with low and high gene importance (Omega), i.e., posterior estimates of gene expression variability (**a**). The left panel shows scRNA-seq profiles of malignant cells of states 1-3 (n=12212) sampled from the Wu dataset, colored by cell state (state 1: red, state 2: blue, state 3: orange). The middle and right panel shows the expression of the gene with the lowest (middle) and the highest (right) gene importance as estimated by Statescope in the deconvolution of the 50 pseudobulk mixtures generated from the Wu dataset. Pearson correlation between Wilcoxon scores from differential gene expression analysis and the gene importance of 15 cell types across 8 scRNA-seq datasets (**b**). Wilcoxon scores were generated by performing a Wilcoxon rank-sum test for each gene between each state and all other states. Only states used for creating pseudobulk mixtures were used in this analysis. Boxplots show PCC distributions. The centre line indicates the median (50th percentile). The box bounds represent the first and third quartiles (25th and 75th percentiles), defining the interquartile range (IQR). Whiskers extend to the minimum and maximum values within 1.5 × IQR of the quartiles. All scRNA-seq dataset PCCs are shown as dots. Source data for supplementary figure 4b are provided as a Source Data File.

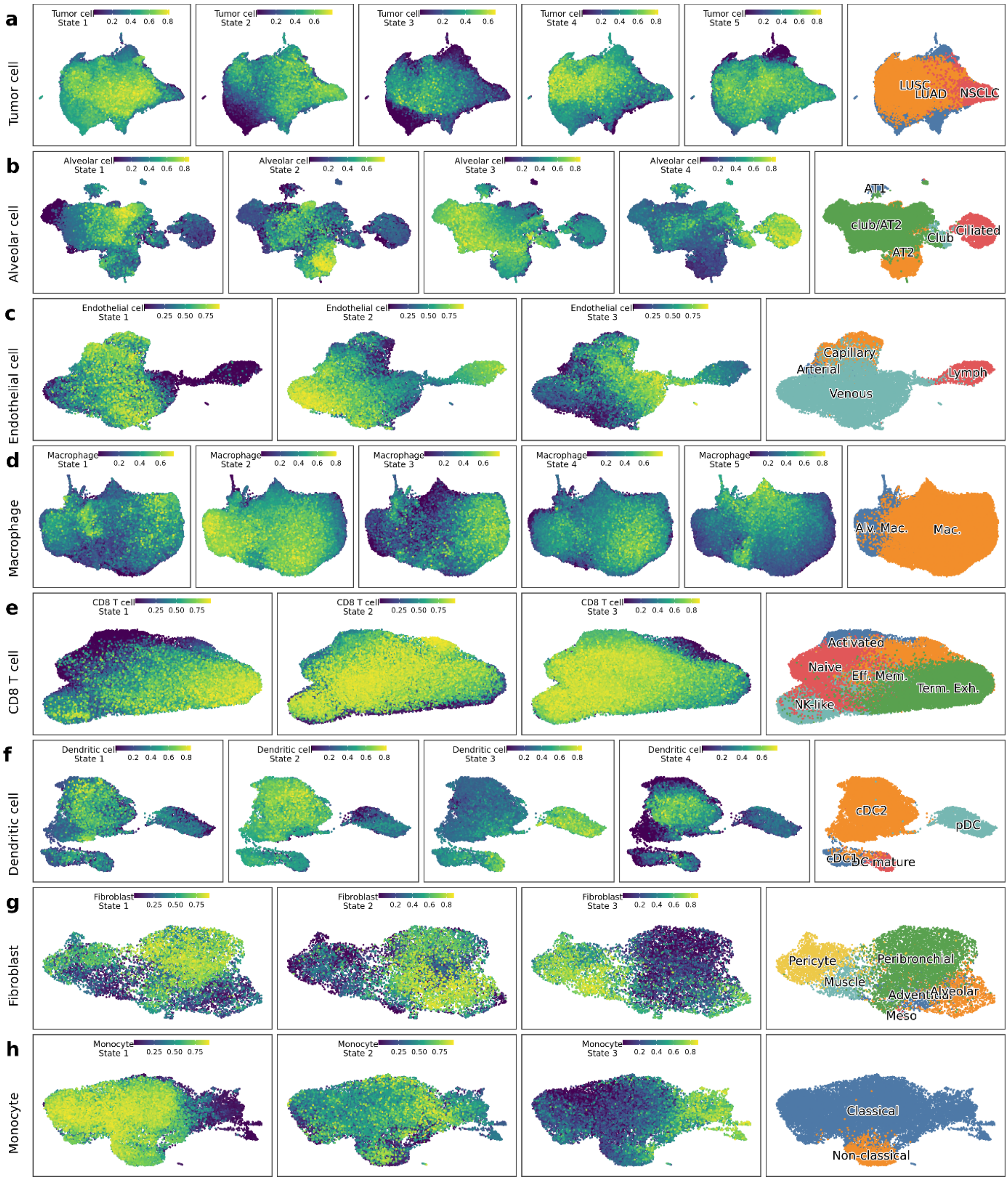

Supplementary Figure 5. Annotation of TCGA-NSCLC cell states using phenotyped Salcher et al scRNA-seq atlas (n=573948 cells). Retrieval of TCGA-NSCLC cell states in Salcher et al scRNA-seq atlas (4) for Malignant cells (n=61695) (**a**), Alveolar cells (n=27509) (**b**), Endothelial cells (n=15907) (**c**), Macrophages (n=60590) (**d**), CD8 T cells (n=65905) (**e**), Dendritic cells (n=16750) (**f**), Fibroblasts (n=9466) (**g**) and Monocytes (n=19652) (**h**). For each row the last column contains the annotations of Salcher et al (4). Only cell types with additional annotations were used. After selecting cells from the specific cell types, the UMAP coordinates were calculated using the embeddings provided by Salcher et al (4).

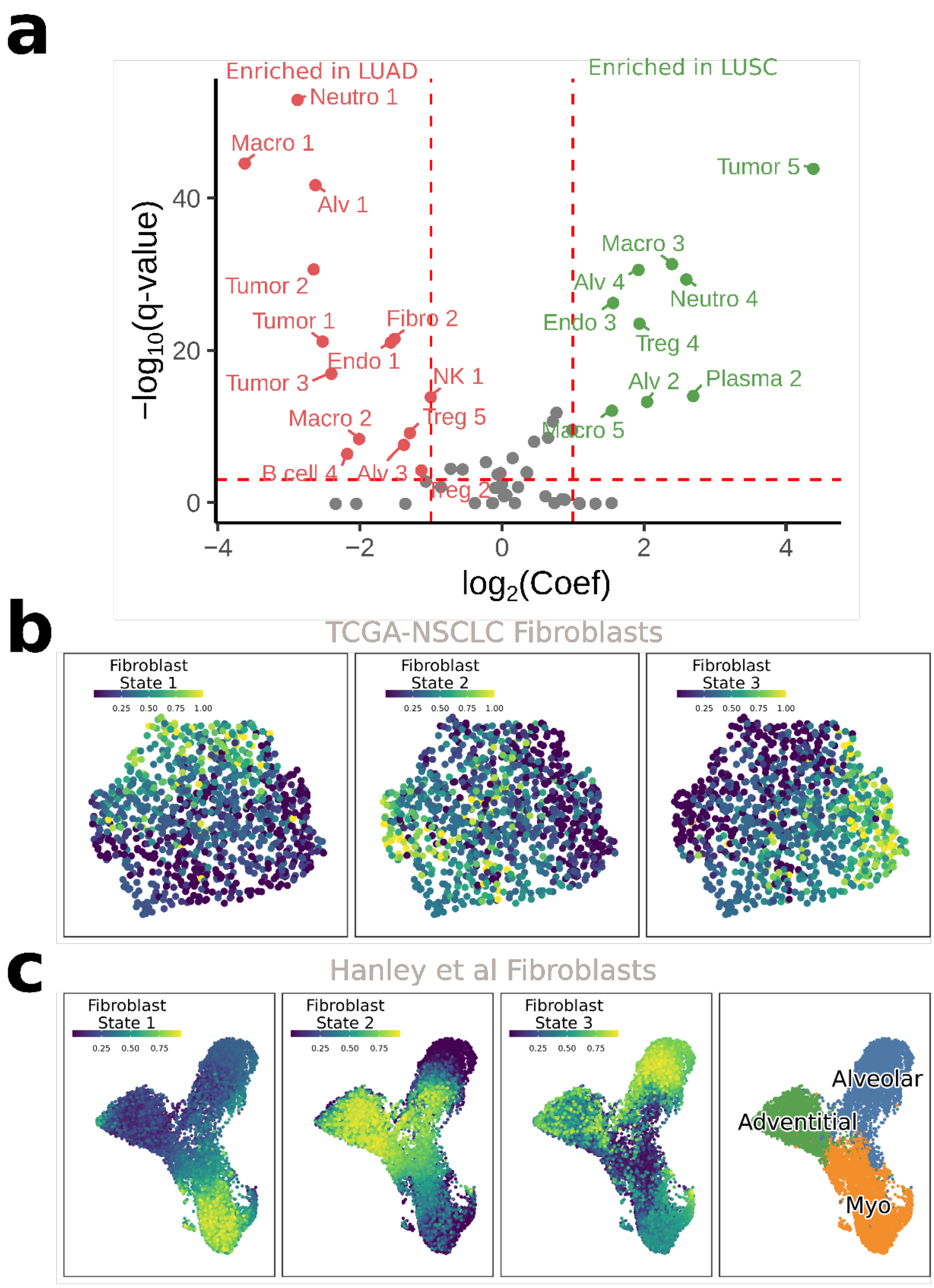

Supplementary Figure 6. Enrichment of NSCLC states in histological subtypes and fibroblast annotation. Volcanoplot depicting the differential enrichment of state scores in LUAD and LUSC samples (n=973 samples) (**a**). The x-axis denotes the $\log_2$ coefficient and the y-axis denotes the $-\log_{10}$ q-values (Benjamini-Hochberg adjusted p-values) of univariate logistic regression models with the subtype as the response variable and the state scores as the covariate for each cell state. Horizontal dashed lines indicate a q-value threshold of 0.001 and vertical dashed lines indicate a $\log_2$ coefficient of 1. Fibroblast state scores in the fibroblasts from the TCGA-NSCLC atlas (n=973 samples) (**b**) and retrieved from the Hanley et al scRNA-seq data (n=9673 cells) (**c**) (21). The annotations (right panel) and embeddings were retrieved from the original publication by Hanley et al. (21). Source data for supplementary figure 6a are provided as a Source Data File.

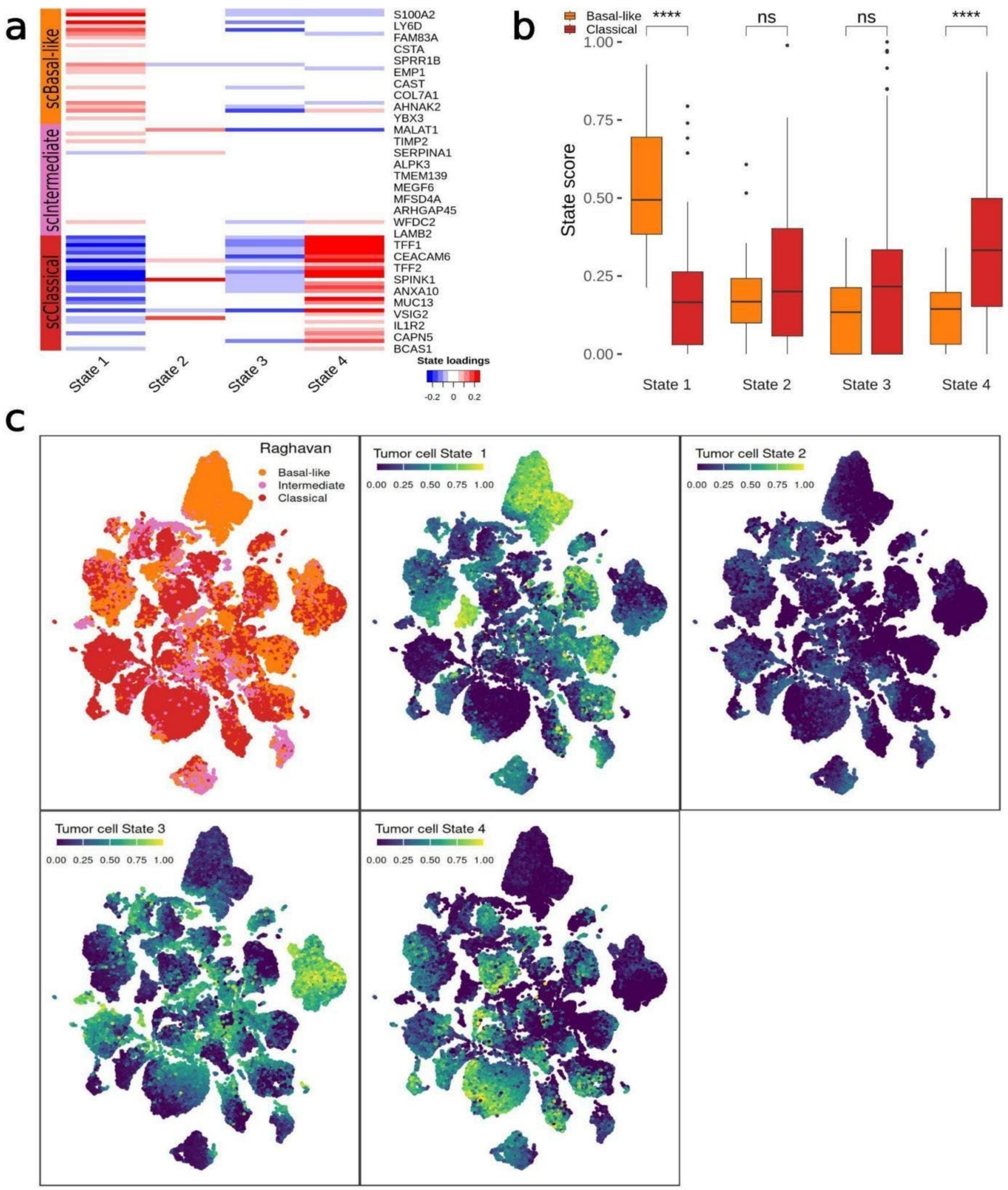

Supplementary Figure 7. Statescope malignant cell states show clear Basal-like and Classical phenotypes. **a)** Heatmap of Statescope malignant cell state loadings for the signature genes from Raghavan et al (26) (**a**). State 1 & State 4 show distinct malignant cell state loadings for the scRNA-seq basal-like and classical genes respectively. State 2 shows a few positive loadings for some classical and some intermediate genes and state 3 shows negative loadings for most selected genes. Comparison of state scores between classical and basal-like bulk RNA subtypes (n=166) classified using PurIST classification (43) (**b**). Boxplots show state score distributions. The centre line indicates the median (50th percentile). The box bounds represent the first and third quartiles (25th and 75th percentiles), defining the interquartile range (IQR). Whiskers extend to the minimum and maximum values within 1.5 × IQR of the quartiles. Points beyond this range are plotted individually. Differences between bulk RNA-seq labels were tested by the two-sided Mann-Whitney U test with ns = $p>0.05$, **** = $p<0.0001$. UMAPs showing the

cells (n=61768) from all samples (n=27) in Werba et al (16), colored according to their tumor subtype and state scores (**c**). Source data for supplementary figure 7a-b are provided as a Source Data File.

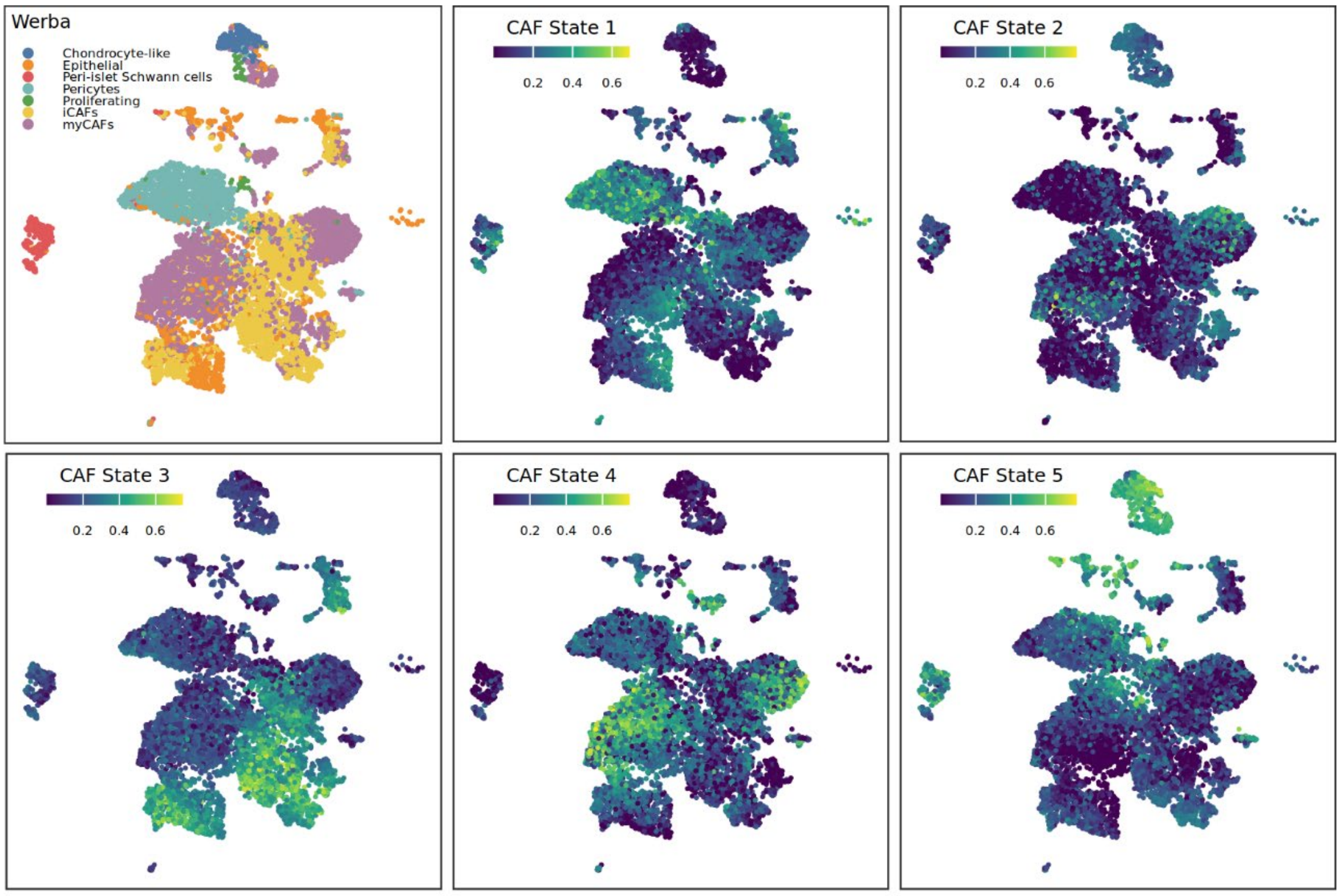

Supplementary Figure 8. scRNA-seq projection analysis of PDAC CAF cell states. In the top left a UMAP projection of the CAF scRNA-seq (n=9731 cells) with their labels from Werba et al (16). To the right of that and underneath, the same projection is colored by the state score of each CAF state. State 1 is enriched with Pericytes, State 2 is mixed, State 3 with iCAFs, State 4 with myCAFs and finally State 5 with multiple minor CAFs subtypes: Chondrocyte-like, Epithelial, Peri-islet Schwann cells & Proliferating CAFs.

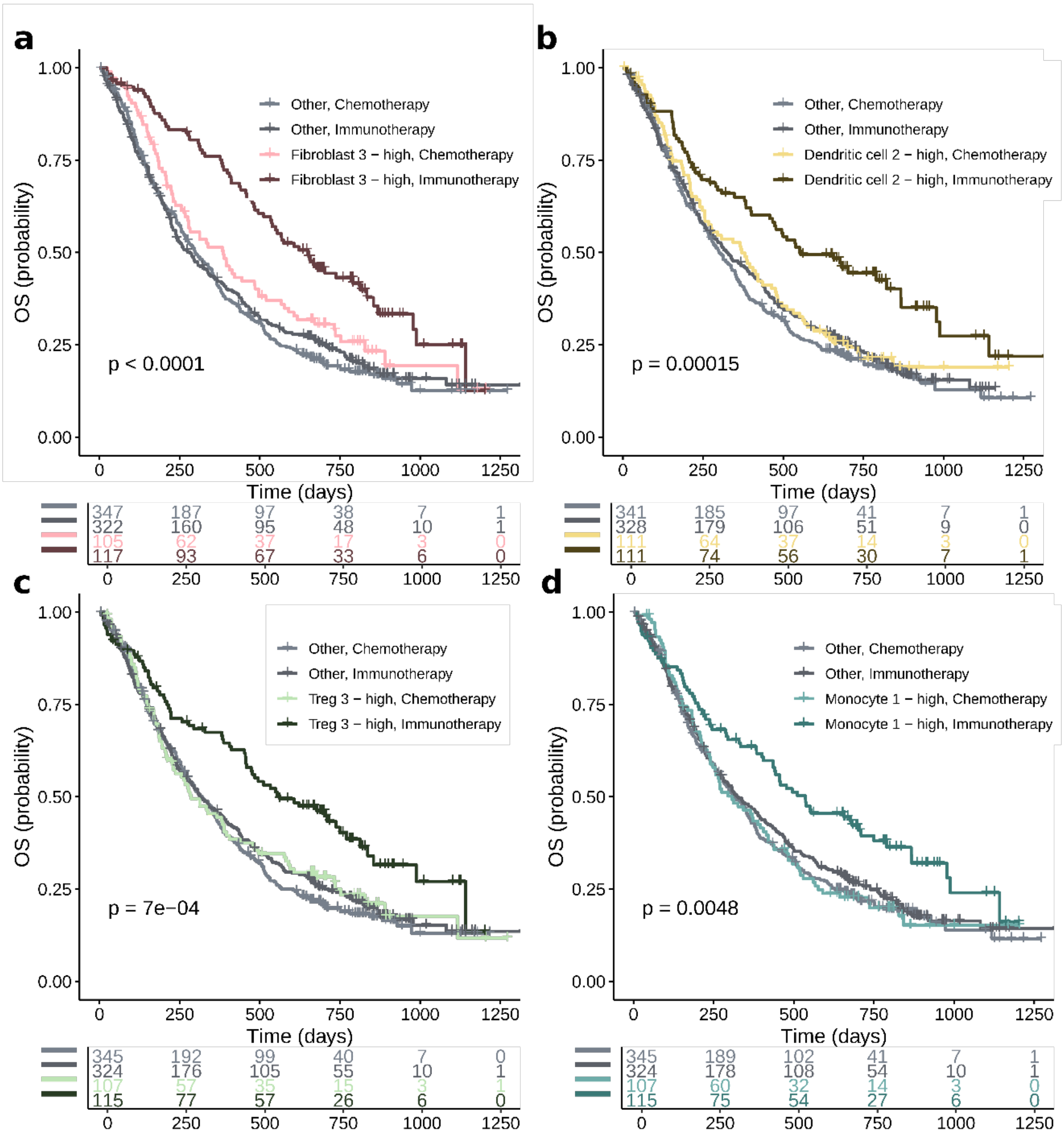

Supplementary Figure 9. States predictive of treatment outcome in POPLAR/OAK. Kaplan-Meier curves of OS for POPLAR/OAK patients (n=891) without (greys) and with 'high' scores for Fibroblast state 3 (**a**), Dendritic cell state 2 (**b**), Regulatory T cell state 3 (**c**) and Monocyte state 1 (**d**), receiving either chemotherapy (light shading) or immunotherapy (dark shading). P-values in Kaplan-Meier plots indicate global significance determined by the log-rank test. The number of patients without an event at different time points are indicated for different groups in the risk tables below the Kaplan-Meier plots. State scores are categorized into 'high' by selecting the upper 75% quantile.

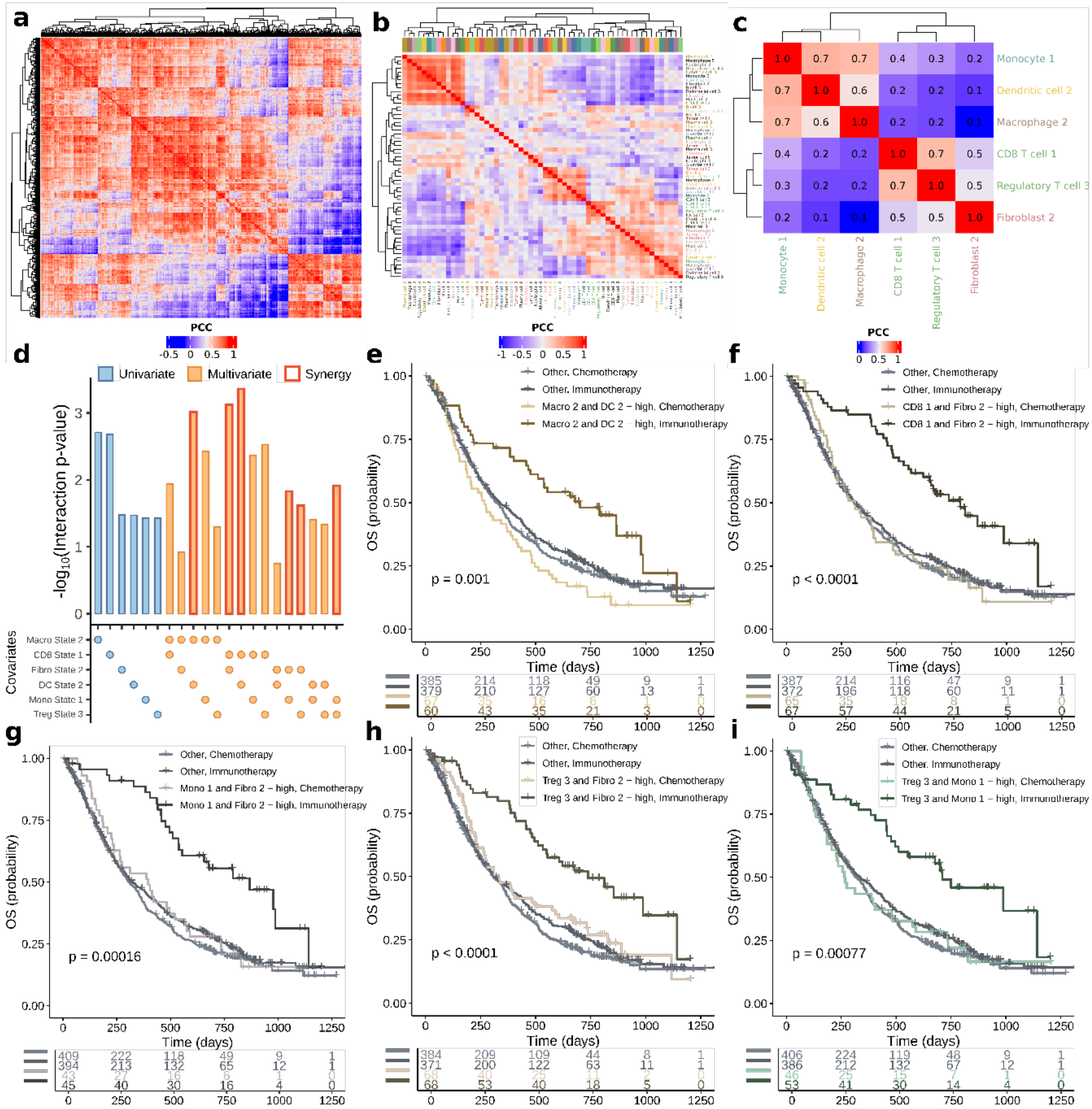

Supplementary Figure 10. Correlation of cell states and multivariate analysis of predictive cell states. Pearson correlation between state scores calculated over sample (n=891) (**a**) and cell states (n=53) (**b**) in the POPLAR/OAK dataset (15). Samples and states were ordered by as shown by the dendrograms. Cell states are annotated by cell type in the columns. Correlation between state scores for states predictive of immunotherapy (**c**). Dendrograms were created using hierarchical clustering with complete linkage and Euclidean distance. Interaction p-value between categorized states in univariate (blue) and multivariate (orange) Cox models of overall survival with treatment arm as the interaction term (**d**). The x-axis denotes the covariates used. States that have higher significance in multivariate models compared to univariate models, and thus display synergy, are outlined in red. Kaplan-Meier curves of OS for patients without (grey) and with 'high' scores for both Macrophage state 2 and Dendritic cell state 2 (**e**), CD8 T cell state 1 and Fibroblast state 2 (**f**), Monocyte state 1 and Fibroblast state 2 (**g**), Regulatory T cell state 3 and Fibroblast state 2 (**h**) and Regulatory T cell state 3 and Monocyte state 1 (**i**). The Kaplan-Meier curves of the patients with 'high' scores for both CD8 T cell state 1 and Dendritic cell state 2 are presented in Figure 5d. P-values in Kaplan-Meier plots indicate global significance determined by the log-rank test. The number of patients without an event at different time points are indicated for different groups in the risk tables below the Kaplan-Meier plots. State scores are categorized into 'high' by selecting the upper 75% quantile. Source data for supplementary figure 10a-d are provided as a Source Data File.

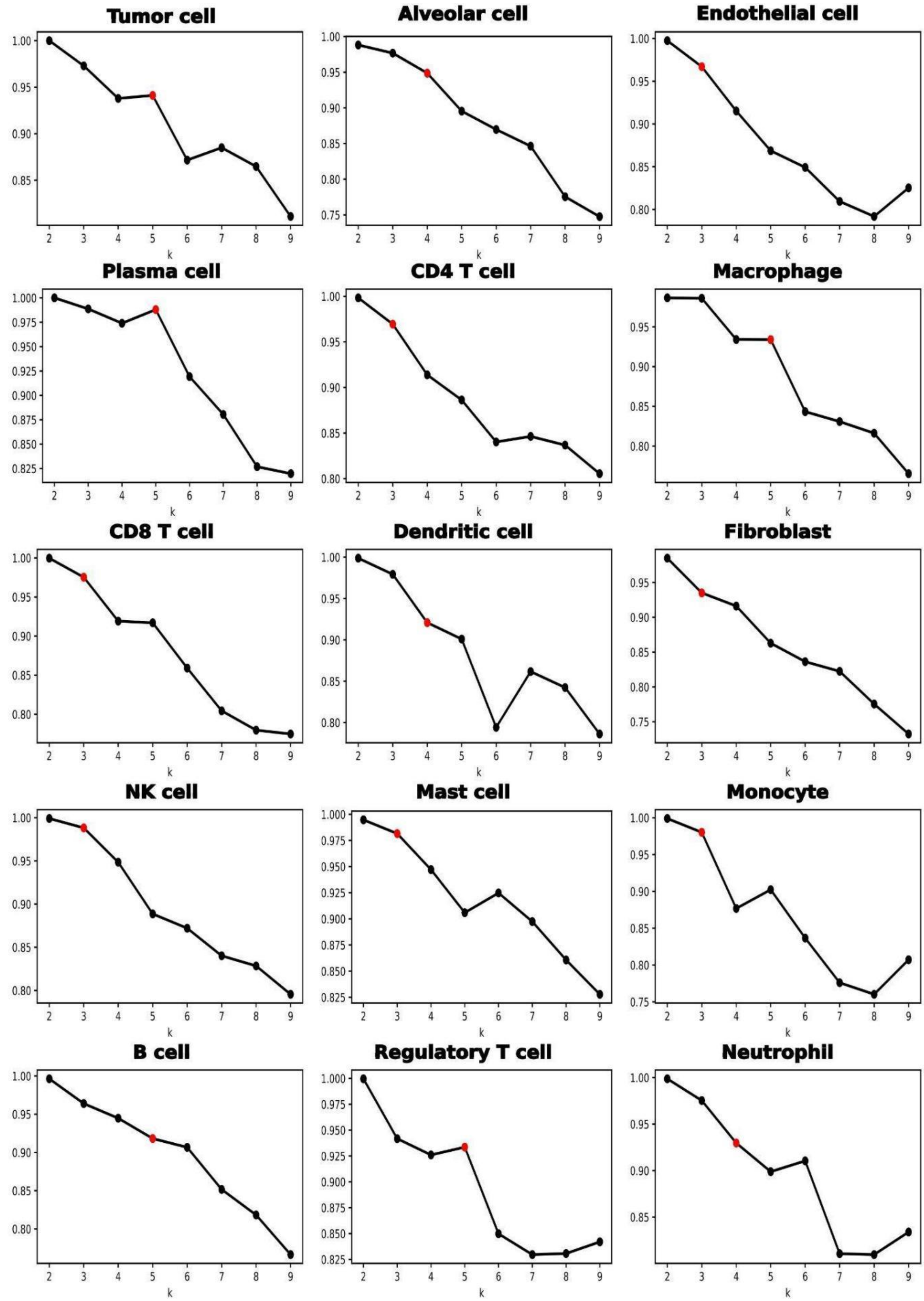

Supplementary Figure 11. Cophenetic coefficient plots for the NSCLC TCGA State Discovery. Cophenetic coefficients of the initial cNMF runs (10 random initializations) for k's ranging from 2-9 for all cell types. The red dots represent the k we chose for the final cNMF run (100 random initializations).

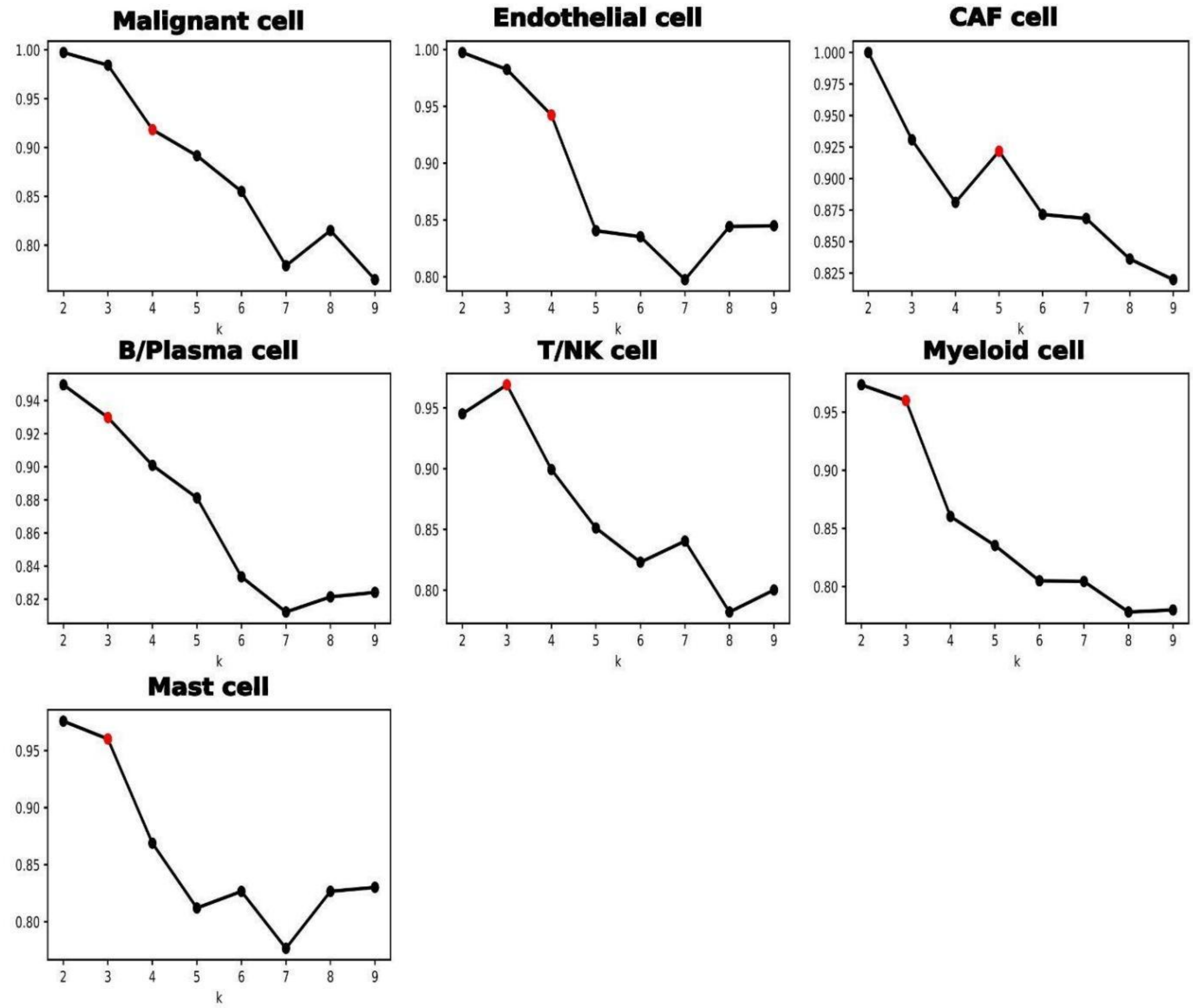

Supplementary Figure 12. Cophenetic coefficient plots for the PDAC TCGA State Discovery.
Cophenetic coefficients of the initial cNMF runs (10 random initializations) for k's ranging from 2-9 for all cell types. The red dots represent the k we chose for the final cNMF run (100 random initializations).

Supplementary Notes

Supplementary Note 1: Estimating cell type subpopulations with Statescope using prior fractions in group-mode

In some instances, prior knowledge of fractions is available for a ‘parent’ cell type but scRNAseq signatures are available for more delineated ‘daughter’ subpopulations of the same cell type lineage (examples shown in Supplementary Figure 13a). To effectively enable the integration of a prior expectation of the ‘parent’ cell type fraction, while further delineating subpopulations in deconvolution (‘group mode’, Supplementary Figure 13b) , Statescope integrates the prior such that the cumulative sum of the ‘daughter’ fractions reflect the prior expectation (Supplementary Figure 13b). This feature enables detailed Statescope analysis of cell populations for which prior fractions are generally scarce (e.g. a T cell fraction prior is easier obtainable than a $CD4^{-}CD8^{-}$ T cell fraction prior).

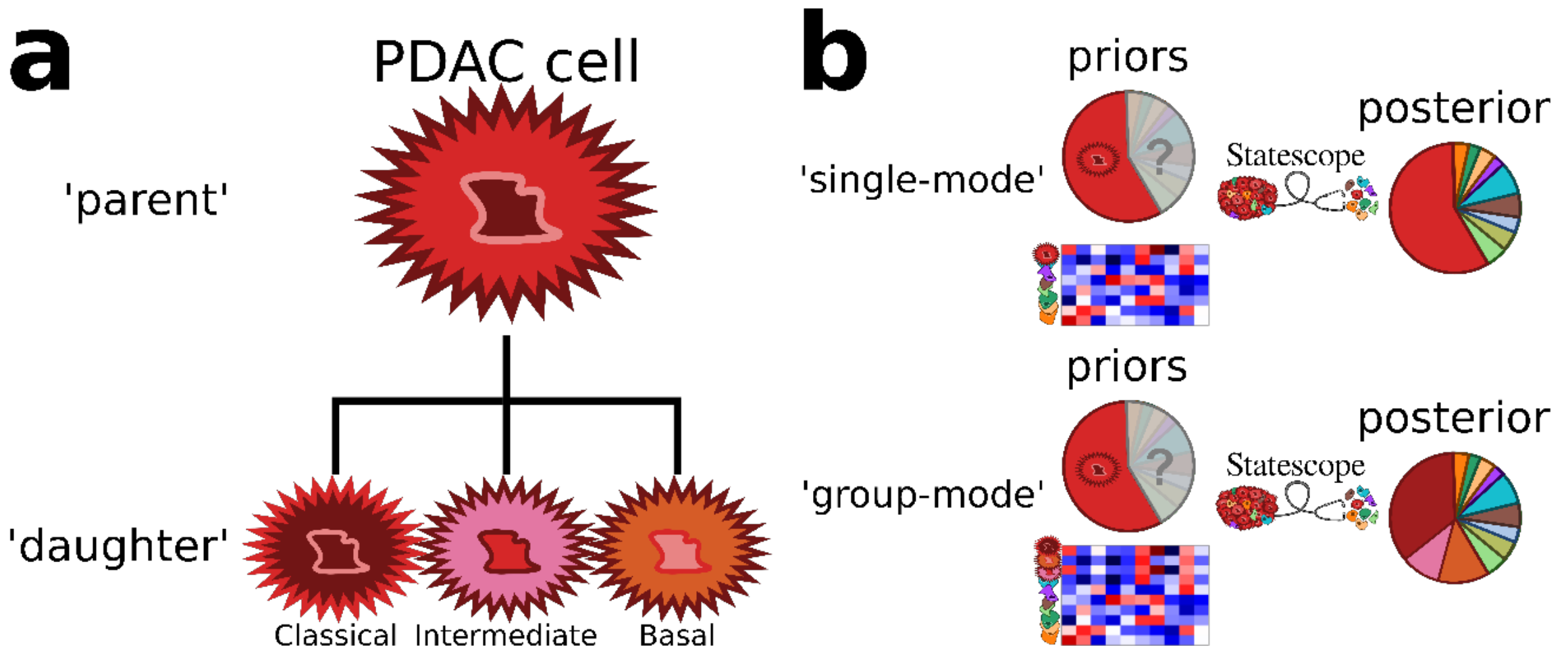

Supplementary Figure 13: Schematic overview of Statescope integration of priors in group-mode. Example of a ‘parent’ cell type with ‘daugther’ subpopulations from the same lineage in PDAC (**a**). Overview of Statescope in ‘single-mode’ and ’group-mode’ (**b**). In both modes, a prior fraction is given for the expected ’parent’ (PDAC cell) fraction. However, in group-mode, scRNA-seq signatures are given as prior knowledge for the ‘daughter’ (classical, intermediate, basal) subpopulations, resulting in posterior fractions of the ‘daughter’ cell types that cumulatively sum to the prior expected ‘parent’ fraction.

Supplementary Note 2: Benchmarking computational efficiency of Statescope

Computational efficiency of Statescope was compared: 1) against an earlier version without PyTorch/CUDA acceleration (44) and 2) against the three baseline methods CIBERSORTx (45), BayesPrism (46) and TAPE (47). We used the 46 PBMC bulk transcriptomes (Supplementary Table 1) to evaluate the performance. The computational time was measured for ten repetitions. All evaluations were carried out on a system with an AMD EPYC 7502 32-Core Processor running on 2.50 GHz and in case of GPU computation on an Nvidia RTX-A100.

First, we compared the PyTorch/CUDA implementation (48) with the non-PyTorch/CUDA version. The optimized version showed a significant speedup for key computational steps

within one optimization iteration: approximately three times faster when run on a CPU and nine times faster when run on the GPU (**Supplementary Figure 14a**). This acceleration in core steps contributes to a notable reduction in overall execution time.

We then compared the computational speed of the Statescope workflow (deconvolution and refinement steps) with the other algorithms using the PBMC dataset (**Supplementary Figure 14b**). Statescope required approximately 2.5 hours when using a GPU for deconvolution and a single CPU core for the refinement step. Utilizing four CPU cores in parallel for the refinement step, Statescope completed the analysis in roughly 45 minutes (¾ of an hour). CIBERSORTx was faster, requiring roughly 30 minutes (½ hour) regardless of using one or four cores. Statescope outperformed BayesPrism in terms of computing time; where Statescope took ~2.5 hours (1 core) or ~45 minutes (4 cores), BayesPrism required approximately 5 hours and 1.5 hours, respectively. Finally, TAPE, run only on GPU here, is by far the fastest method, completing the analysis within ~5 minutes. This was in-line with expectation as it is a deep learning based model, heavily optimized for GPU acceleration.

Overall, the PyTorch/CUDA implementation significantly improves the performance of Statescope in terms of computing time. The benchmark results show that Statescope offers competitive performance, being substantially faster than BayesPrism and comparable to CIBERSORTx with multiple cores.

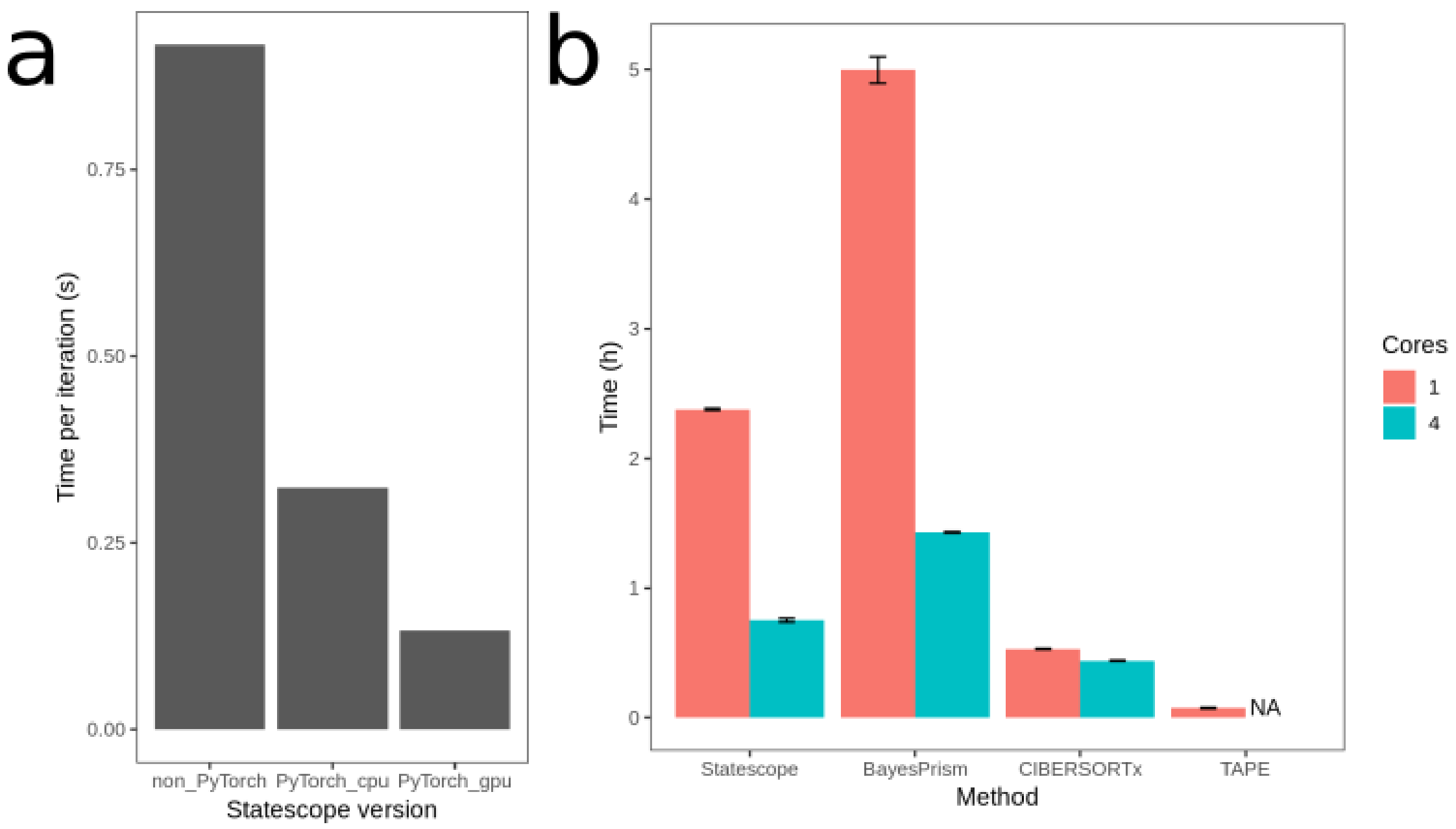

Supplementary Figure 14: Computational evaluation of Statescope. a) Shows the benefit of the PyTorch/CUDA implementation over the earlier version. The average time per iteration was measured over 1000 iterations. The earlier version (non_PyTorch) is compared to the new PyTorch version on cpu (PyTorch_cpu) and gpu (PyTorch_gpu). b**)** Compares Statescope with the other baseline methods. Here the average time consumption is measured for the deconvolution and refinement steps of Statescope vs the other methods which do all in one step. Time is measured over 10 repetitions with black error bars showing the standard deviations of these runs. Note that TAPE is only run on a GPU and thus does not have a 4 core result. Source data are provided as a Source Data File.

Supplementary Note 3: Patients, laboratory procedures and RNA sequencing of patient blood samples with known cell type fractions

Blood was drawn from 48 patients undergoing surgery for glioblastoma (n=20), meningioma (n=16) or astrocytoma (n=6), as well as healthy donors (n=6). PBMCs were isolated using gradient density centrifugation with lymphoprep (Axis-Shield). Multiple vials of PBMCs were cryopreserved per donor, which were partly used in a previous CyTOF study (2) (Supplementary Figure 15). 48 Donors for which CyTOF data was available were included in the present study. In CyTOF only live cells are phenotyped, therefore only live cells were used for RNA isolation to obtain a matching dataset. First PBMCs were thawed through dropwise addition of IMDM (Gibco) with 10% fetal calf serum (FCS, Biowest), 100 IU Penicillin and 100 IU Streptomycin (Lonza) at 37 °C. Thawed cells were centrifuged at 500 g for 8 min at 4 °C. Cell pellets were taken up in cold sterile-filtered 0.1% BSA (Roche) in PBS (Fresenius Kabi) and again centrifuged. Washed cells were stained with ice cold 7-AAD in 0.1% BSA in PBS. Live cells were sorted at 5 °C using a FACS Aria Fusion (BD Biosciences) with IMDM with 20% FCS, 100 IU Penicillin and 100 IU Streptomycin as collection medium. After sorting 0.7-1 million live cells collection tubes were immediately centrifuged for 8 min at 500 g and 4 °C. The supernatant was poured off, the remaining liquid was used to resuspend the cell pellet before addition of TRIzol™ Reagent (Invitrogen; Thermo Fisher Scientific). RNA isolation was performed according to the manufacturer's protocol. Remaining TRIzol™ contaminations were removed using the buthanol extraction protocol described by Krebs et al (49). RNA dissolved in water was mixed with water-saturated 1-buthanol (Sigma-Aldrich), briefly centrifuged, followed by removal of the upper organic layer. After repeating the previous step once with 1-buthanol, these steps were performed twice with water-saturated diethyl ether (Riedel-de Haën). The final diethyl ether layer was left to evaporate in a fume hood for 15 minutes. RNA quality was assessed using the Agilent RNA 6000 Pico Kit and the 2100 Bioanalyzer (Agilent Technologies). Samples with an RNA integrity number (RIN) value of at least 6.9 and a yield of at least 0.72 µg included for library preparation using the KAPA mRNA HyperPrep Kit (Roche) and were sequenced on a Novaseq 6000 instrument (Illumina). Two samples with ~16% and ~50% of undefined fractions in CyTOF were excluded from analysis.

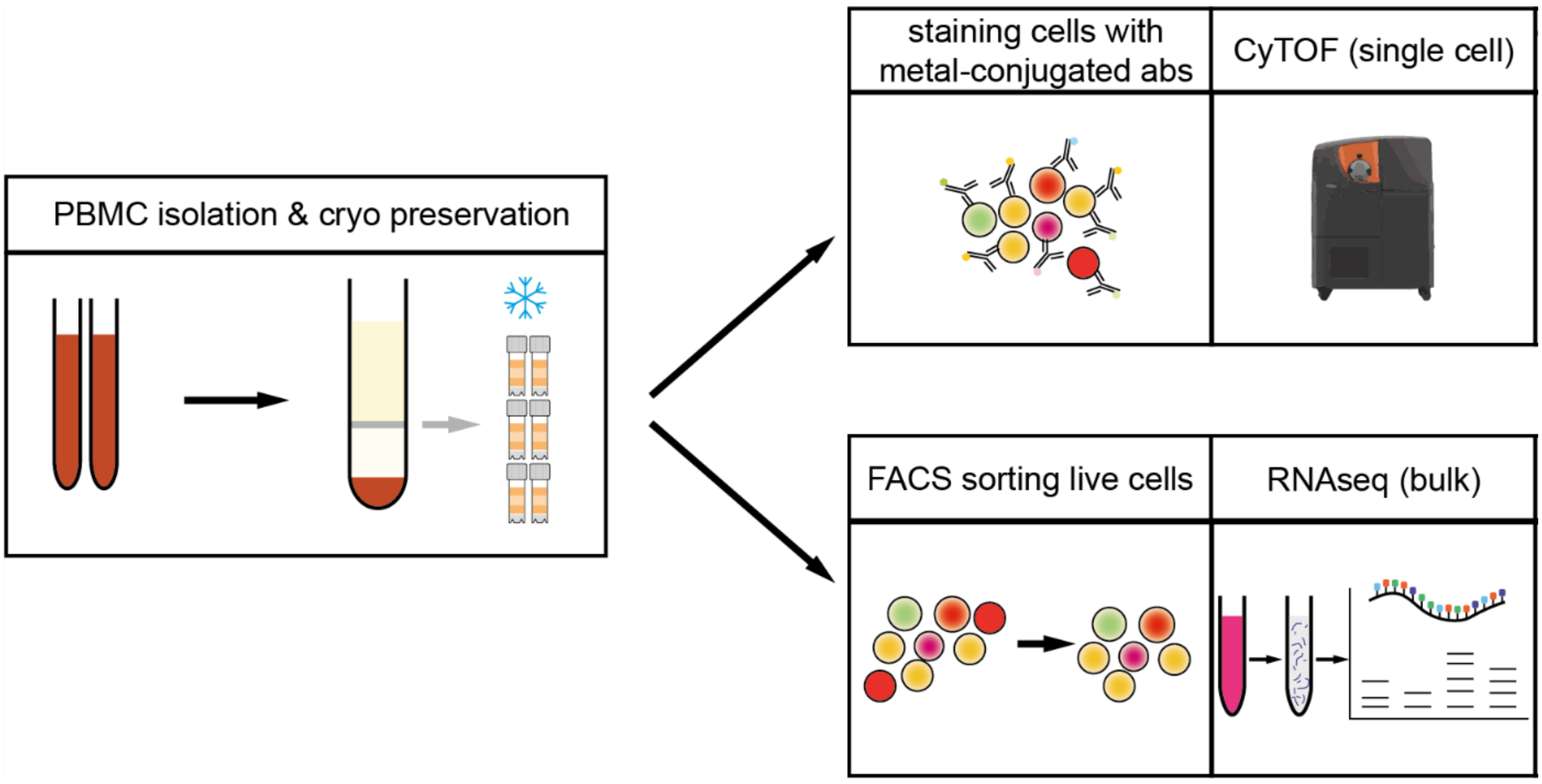

Supplementary Figure 15: Overview of PBMC RNAseq and CyToF experiments.

Supplementary Note 4: Effect of cell type and dataset characteristics on Statescope deconvolution performance

Leveraging the NSCLC simulation datasets with 15 cell types across 8 datasets, we further investigated the effect of cell type- and dataset characteristics on the performance measures of Statescope presented in **Figure 1g,h,j** and **Figure 2f**. We observed a clear association between the average abundance and the number of cells with the cell type heterogeneity (**Supplementary Figure 16a-b**). When comparing the fraction estimation performance presented in Supplementary Figure 16g (RMSE) with Supplementary Figure 16h (PCC), we observed that Statescope performed particularly poorly in terms of PCC for the Lambrechts and UKIM-V datasets but not RMSE. In Supplementary Figure 16c, we show that for these particular datasets, large variation in fraction estimation PCC can be observed across cell types. We observed a clear trend between the number of samples used in the deconvolution experiment with the PCC as well as a decrease in PCC variation. The low fraction estimation performance observed in these datasets is thus a direct effect of the imprecise PCC estimate caused by the low number of samples (n=3). We observed a strong correlation between the average abundance and the fraction RMSE (**d**). This is expected because the scale of the (true) fraction is directly linked to the measured error.

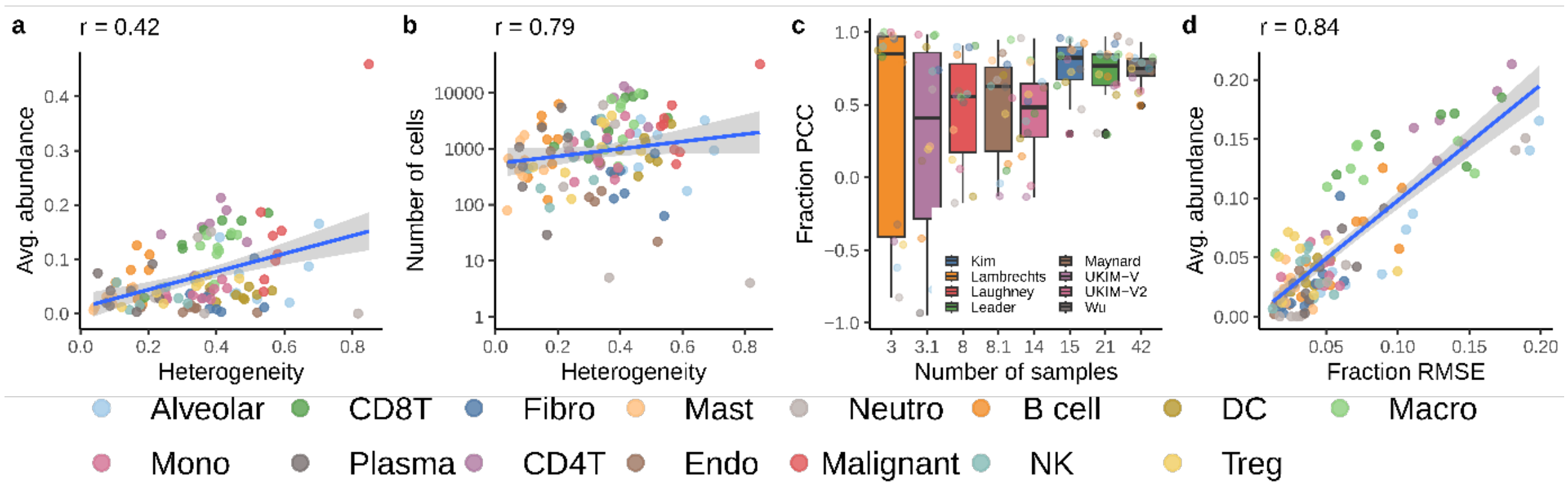

Supplementary Figure 16: Correlation of cell type characteristics and influence of number of samples on fraction estimation PCC. Average cell type abundance (**a**) and number of cells (**b**) plotted against the cell type heterogeneity for each of the 8 datasets (n=278908 cells from 114 total samples). The heterogeneity is calculated by subtracting the average Pearson correlation between all cells within a cell type from 1. Boxplots of the fraction estimation performance against the number of samples, colored by dataset (**c**). The jittered points indicate the fraction estimation performance per cell type. Boxplots show PCC distributions. The centre line indicates the median (50th percentile). The box bounds represent the first and third quartiles (25th and 75th percentiles), defining the interquartile range (IQR). Whiskers extend to the minimum and maximum values within 1.5 × IQR of the quartiles. All celltype PCCs are shown as dots. Average cell type abundance and plotted against the fraction estimation performance as measured by the RMSE (**d**). For (**c**) and (**d**) the fraction estimation performance of the malignant cells, which is given as a prior, is excluded. For the scatter plots, the correlation between the two plotted variables is indicated by the linear regression line (blue line, grey area indicates the confidence interval) and the Pearson correlation (*r*) indicated in the top left corner. The point legend below the plots indicates the colors per cell type. Source data are provided as a Source Data File.

Next, we evaluated the effect of the abundance and heterogeneity on the fraction estimation (Supplementary Figure 17a), cell type-specific gene expression profile estimation (Supplementary Figure 17b), and supervised/unsupervised state recovery performance of Statescope (Supplementary Figure 17c-d). We observed no correlation between cellular abundance and a slight negative correlation between heterogeneity with the fraction estimation

performance (Supplementary Figure 17a). This indicates that in terms of fraction estimation, Statescope can deal similarly with both rare and abundant cell types, but suffers slightly from cell types with high gene expression heterogeneity. We observed a moderate correlation between cellular abundance and no correlation with the gene expression estimation performance (Supplementary Figure 17b). This indicates that in terms of gene expression estimation, Statescope can deal with heterogeneous cell types, but suffers slightly from cell types with low abundance. Nevertheless, the expression PCC is relatively high for any of the cell types (PCC>0.5) including those with low abundance. In terms of supervised (Supplementary Figure 17c) and unsupervised (Supplementary Figure 17d) state recovery, moderate correlations between the abundance and the heterogeneity was observed. This observation can be explained since a higher heterogeneity is associated with a clearer distinction between cell states within the cell type, which makes it easier to distinguish these states in both the supervised and unsupervised state recovery analyses. In this analysis, the abundance of cell types is likely a proxy for the heterogeneity, which showed a weaker correlation with the state recovery performance.

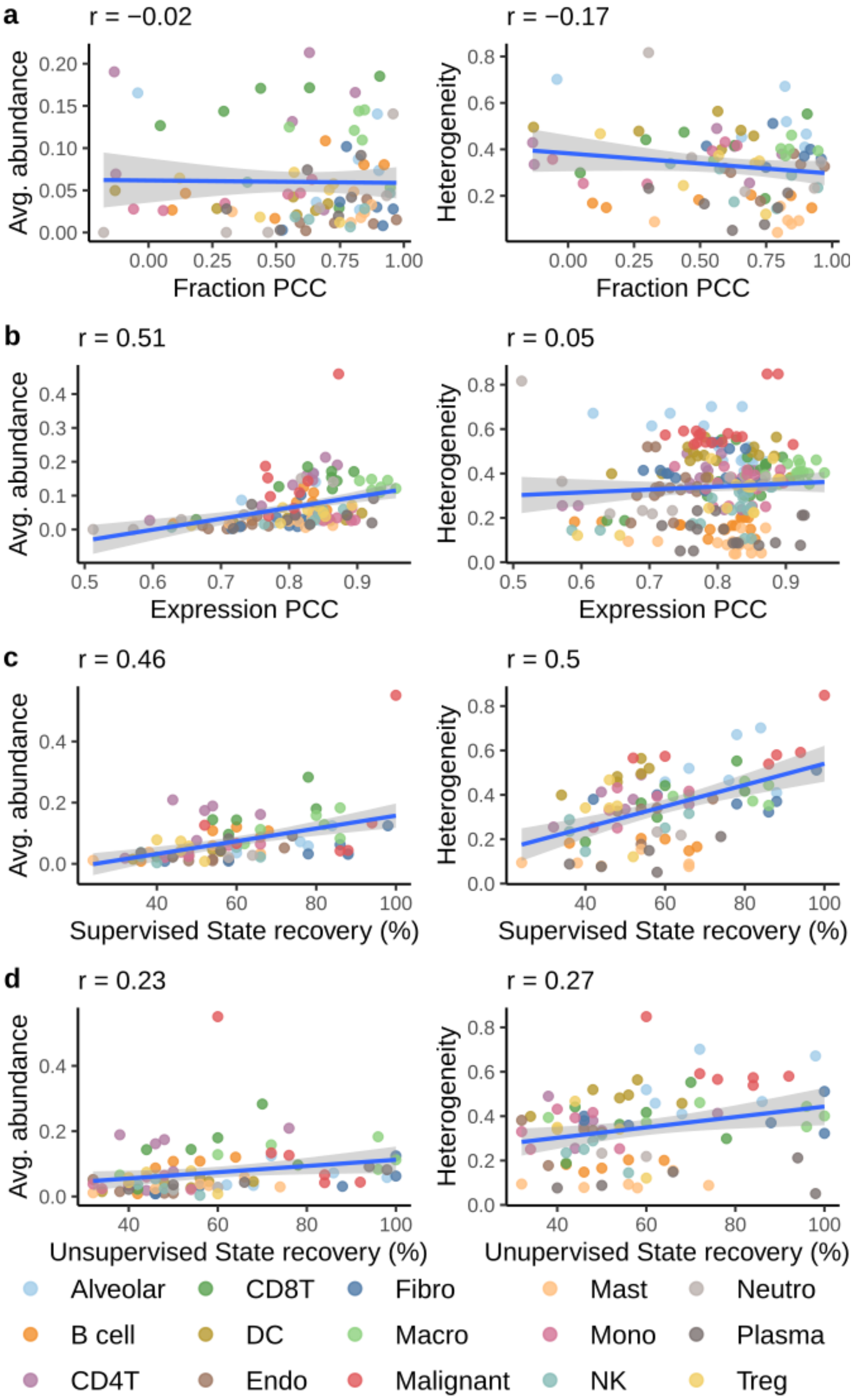

Supplementary Figure 17: Correlation of abundance and heterogeneity on Statescope deconvolution performance. Average cell type abundance (left) and heterogeneity (right) plotted against the fraction estimation performance (**a**), cell type-specific gene expression estimation performance (**b**), supervised state recovery (**c**) and unsupervised state recovery (**d**) using the 8 NSCLC scRNAseq datasets (n=278908 cells from 114 total samples). Performance metrics shown in **a** and **b** relate to simulation experiments presented in Figure 1 and **c** and **d** relate to Figure 2. In Figure 1a, fraction estimation performance from the UKIM-V (n=11269 cells from 3 samples) and Lambrechts (n=28552 cells from 3 samples) are excluded. Moreover, the fraction estimation performance of the malignant cells, which is given as a prior, is excluded. The correlation between the two plotted variables is indicated by the linear regression line (blue line, grey area indicates confidence interval) and the Pearson correlation (*r*) indicated in the top left corner. The heterogeneity is calculated by subtracting the average Pearson correlation between all cells within a cell type from 1. The point legend below the plots indicates the colors per cell type. Source data are provided as a Source Data File.

Supplementary Note 5: Theoretical comparison between Statescope and benchmark methods

A key technical advancement in Statescope is its Bayesian deconvolution strategy, an enhanced version of BLADE (44). Bayesian models such as Statescope and BayesPrism (46) have the advantage of modeling both cell type-specific gene expression profiles and cell fractions with a restricted number of parameters. In contrast, CIBERSORTx (45) requires two separate steps where scRNA-seq signatures are not accounted for the estimation of cell type-specific gene expression profiles, and TAPE (47) relies on an excessive number of parameters (>10 million for 10,000 genes). All baseline methods demonstrated competitive performance in cell fraction estimation, except for absolute fraction estimation, where Statescope outperformed due to its ability to integrate prior expectations of cell fractions (**Figure 1c-h**). However, the performance gap widened when estimating cell type-specific gene expression profiles (**Figure 1i-k** and **Figure 2b**). CIBERSORTx dropped out in multiple benchmarks, as it cannot infer cell type-specific gene expression profiles with small sample sizes. TAPE exhibited limited performance, likely due to overfitting caused by its large number of parameters and the fact that bulk data in the benchmarking datasets were independently generated from the scRNA-seq data used for training.

A key distinction between the two Bayesian methods, Statescope and BayesPrism, is their underlying assumption for modeling gene expression variability. BayesPrism employs a multinomial distribution, where there is only one parameter to account for the relative expression level of each gene in each cell type. The gene expression variability is then implicitly determined by total read counts. In contrast, Statescope adopts a log-normal distribution, which explicitly incorporates two parameters to account for cell type-specific gene expression levels and variations observed in scRNA-seq data. This comprehensive modeling of gene expression variability allows Statescope to achieve an enhanced capacity to capture inter-sample variability in both tumor and non-malignant cell states, including those not observed in scRNA-seq data (**Figure 2d-e**).

Supplementary Note 6: Comparison of Gaussian noise and estimation tool-based noise.

To study the effect of noise on prior information we added different levels of Gaussian noise to the prior and studied its impact (Supplementary Figure 2). To evaluate how comparable Gaussian noise was to a more realistic source of noise, we compared the Gaussian noise with the difference between tumor fraction estimation tools. ACE, used in our manuscript, based on copy number profiles and ABSOLUTE based on mutational data. When compared with the distributions of the Gaussian noise levels, it seems then that this tumor purity estimation noise, defined as ACE-ABSOLUTE estimates, is very similar to our Gaussian low noise setting, shifted a bit to the left (Supplementary Figure 18).

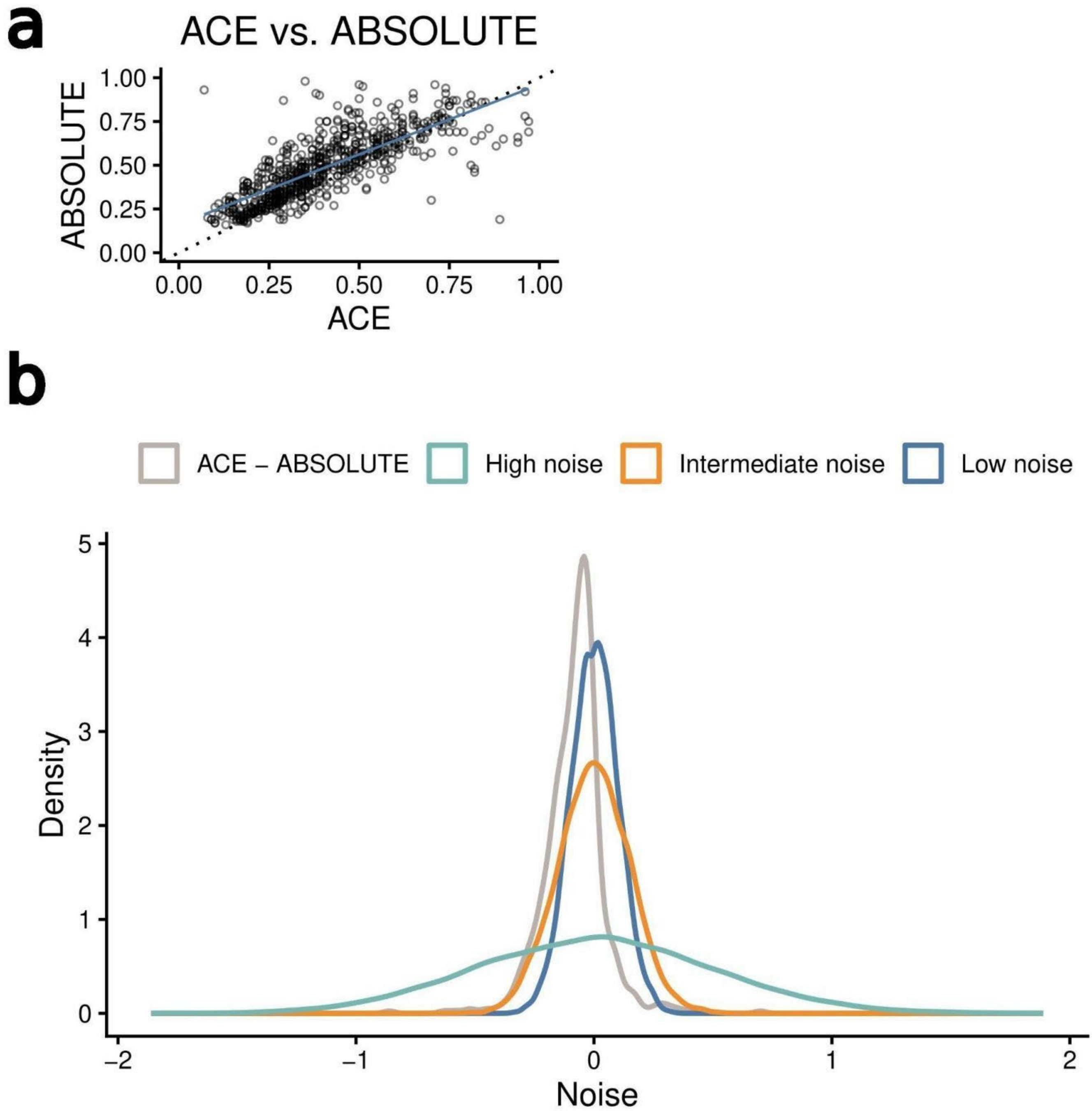

Supplementary Figure 18: Comparison of different Gaussian noise levels with a more realistic estimation tool-based noise. Estimated tumor fractions by ABSOLUTE against estimated tumor fractions by ACE in 663 TCGA samples (a). Density plot comparing the low, intermediate and high Gaussian noise levels with the difference between the ACE and ABSOLUTE tumor fraction estimations (b). Source data are provided as a Source Data File.

Supplementary References

1. Stephenson E, Reynolds G, Botting RA, Calero-Nieto FJ, Morgan MD, Tuong ZK, et al. Single-cell multi-omics analysis of the immune response in COVID-19. Nat Med. 2021;27(5):904-16.
2. Dusoswa SA, Verhoeff J, van Asten S, Lubbers J, van den Braber M, Peters S, et al. The immunological landscape of peripheral blood in glioblastoma patients and immunological consequences of age and dexamethasone treatment. Front Immunol. 2024;15:1343484.
3. Hoek KL, Samir P, Howard LM, Niu X, Prasad N, Galassie A, et al. A cell-based systems biology assessment of human blood to monitor immune responses after influenza vaccination. PLoS One. 2015;10(2):e0118528.
4. Salcher S, Sturm G, Horvath L, Untergasser G, Kuempers C, Fotakis G, et al. High-resolution single-cell atlas reveals diversity and plasticity of tissue-resident neutrophils in non-small cell lung cancer. Cancer Cell. 2022;40(12):1503-20 e8.
5. Zilionis R, Engblom C, Pfirschke C, Savova V, Zemmour D, Saatcioglu HD, et al. Single-Cell Transcriptomics of Human and Mouse Lung Cancers Reveals Conserved Myeloid Populations across Individuals and Species. Immunity. 2019;50(5):1317-34 e10.
6. Kim N, Kim HK, Lee K, Hong Y, Cho JH, Choi JW, et al. Single-cell RNA sequencing demonstrates the molecular and cellular reprogramming of metastatic lung adenocarcinoma. Nat Commun. 2020;11(1):2285.
7. Lambrechts D, Wauters E, Boeckx B, Aibar S, Nittner D, Burton O, et al. Phenotype molding of stromal cells in the lung tumor microenvironment. Nat Med. 2018;24(8):1277-89.
8. Laughney AM, Hu J, Campbell NR, Bakhoum SF, Setty M, Lavallee VP, et al. Regenerative lineages and immune-mediated pruning in lung cancer metastasis. Nat Med. 2020;26(2):259-69.
9. Leader AM, Grout JA, Maier BB, Nabet BY, Park MD, Tabachnikova A, et al. Single-cell analysis of human non-small cell lung cancer lesions refines tumor classification and patient stratification. Cancer Cell. 2021;39(12):1594-609 e12.
10. Maynard A, McCoach CE, Rotow JK, Harris L, Haderk F, Kerr DL, et al. Therapy-Induced Evolution of Human Lung Cancer Revealed by Single-Cell RNA Sequencing. Cell. 2020;182(5):1232-51 e22.
11. Wu F, Fan J, He Y, Xiong A, Yu J, Li Y, et al. Single-cell profiling of tumor heterogeneity and the microenvironment in advanced non-small cell lung cancer. Nat Commun. 2021;12(1):2540.
12. Cancer Genome Atlas Research N. Comprehensive genomic characterization of squamous cell lung cancers. Nature. 2012;489(7417):519-25.
13. Cancer Genome Atlas Research N. Comprehensive molecular profiling of lung adenocarcinoma. Nature. 2014;511(7511):543-50.
14. Priestley P, Baber J, Lolkema MP, Steeghs N, de Bruijn E, Shale C, et al. Pan-cancer whole-genome analyses of metastatic solid tumours. Nature. 2019;575(7781):210-6.
15. Patil NS, Nabet BY, Muller S, Koeppen H, Zou W, Giltnane J, et al. Intratumoral plasma cells predict outcomes to PD-L1 blockade in non-small cell lung cancer. Cancer Cell. 2022;40(3):289-300 e4.
16. Werba G, Weissinger D, Kawaler EA, Zhao E, Kalfakakou D, Dhara S, et al. Single-cell RNA sequencing reveals the effects of chemotherapy on human pancreatic adenocarcinoma and its tumor microenvironment. Nat Commun. 2023;14(1):797.
17. Cancer Genome Atlas Research Network. Electronic address aadhe, Cancer Genome Atlas Research N. Integrated Genomic Characterization of Pancreatic Ductal Adenocarcinoma. Cancer Cell. 2017;32(2):185-203 e13.
18. Meylan M, Petitprez F, Becht E, Bougouin A, Pupier G, Calvez A, et al. Tertiary lymphoid structures generate and propagate anti-tumor antibody-producing plasma cells in renal cell cancer. Immunity. 2022;55(3):527-41 e5.
19. Szabo PA, Levitin HM, Miron M, Snyder ME, Senda T, Yuan J, et al. Single-cell transcriptomics of human T cells reveals tissue and activation signatures in health and disease. Nat Commun. 2019;10(1):4706.

20. Ma RY, Black A, Qian BZ. Macrophage diversity in cancer revisited in the era of single-cell omics. Trends Immunol. 2022;43(7):546-63.
21. Hanley CJ, Waise S, Ellis MJ, Lopez MA, Pun WY, Taylor J, et al. Single-cell analysis reveals prognostic fibroblast subpopulations linked to molecular and immunological subtypes of lung cancer. Nat Commun. 2023;14(1):387.
22. Rebuffet L, Melsen JE, Escaliere B, Basurto-Lozada D, Bhandoola A, Bjorkstrom NK, et al. High-dimensional single-cell analysis of human natural killer cell heterogeneity. Nat Immunol. 2024;25(8):1474-88.
23. Wu C, Boey D, Bril O, Grootens J, Vijayabaskar MS, Sorini C, et al. Single-cell transcriptomics reveals the identity and regulators of human mast cell progenitors. Blood Adv. 2022;6(15):4439-49.
24. Fitzsimons E, Qian D, Enica A, Thakkar K, Augustine M, Gamble S, et al. A pan-cancer single-cell RNA-seq atlas of intratumoral B cells. Cancer Cell. 2024;42(10):1784-97 e4.
25. Miragaia RJ, Gomes T, Chomka A, Jardine L, Riedel A, Hegazy AN, et al. Single-Cell Transcriptomics of Regulatory T Cells Reveals Trajectories of Tissue Adaptation. Immunity. 2019;50(2):493-504 e7.
26. Raghavan S, Winter PS, Navia AW, Williams HL, DenAdel A, Lowder KE, et al. Microenvironment drives cell state, plasticity, and drug response in pancreatic cancer. Cell. 2021;184(25):6119-37 e26.
27. Moffitt RA, Marayati R, Flate EL, Volmar KE, Loeza SG, Hoadley KA, et al. Virtual microdissection identifies distinct tumor- and stroma-specific subtypes of pancreatic ductal adenocarcinoma. Nat Genet. 2015;47(10):1168-78.
28. Weisbrod AB, Zhang L, Jain M, Barak S, Quezado MM, Kebebew E. Altered PTEN, ATRX, CHGA, CHGB, and TP53 expression are associated with aggressive VHL-associated pancreatic neuroendocrine tumors. Horm Cancer. 2013;4(3):165-75.
29. Kim HS, Yoon JH, Baek GO, Yoon MG, Han JE, Cho HJ, et al. Tumor Endothelial Cells-Associated Integrin Alpha-6 as a Promising Biomarker for Early Detection and Prognosis of Hepatocellular Carcinoma. Cancers (Basel). 2023;15(16).
30. Jin H, Zheng W, Hou J, Peng H, Zhuo H. An Essential NRP1-Mediated Role for Tagln2 in Gastric Cancer Angiogenesis. Front Oncol. 2021;11:653246.
31. Wuttig D, Zastrow S, Fussel S, Toma MI, Meinhardt M, Kalman K, et al. CD31, EDNRB and TSPAN7 are promising prognostic markers in clear-cell renal cell carcinoma revealed by genome-wide expression analyses of primary tumors and metastases. Int J Cancer. 2012;131(5):E693-704.
32. Kazakova AN, Lukina MM, Anufrieva KS, Bekbaeva IV, Ivanova OM, Shnaider PV, et al. Exploring the diversity of cancer-associated fibroblasts: insights into mechanisms of drug resistance. Front Cell Dev Biol. 2024;12:1403122.
33. Helms E, Onate MK, Sherman MH. Fibroblast Heterogeneity in the Pancreatic Tumor Microenvironment. Cancer Discov. 2020;10(5):648-56.
34. Cords L, Tietscher S, Anzeneder T, Langwieder C, Rees M, de Souza N, et al. Cancer-associated fibroblast classification in single-cell and spatial proteomics data. Nat Commun. 2023;14(1):4294.
35. Qian CS, Li LJ, Huang HW, Yang HF, Wu DP. MYC-regulated lncRNA NEAT1 promotes B cell proliferation and lymphomagenesis via the miR-34b-5p-GLI1 pathway in diffuse large B-cell lymphoma. Cancer Cell Int. 2020;20:87.
36. Shaffer AL, Shapiro-Shelef M, Iwakoshi NN, Lee AH, Qian SB, Zhao H, et al. XBP1, downstream of Blimp-1, expands the secretory apparatus and other organelles, and increases protein synthesis in plasma cell differentiation. Immunity. 2004;21(1):81-93.
37. Campbell AR, Regan K, Bhave N, Pattanayak A, Parihar R, Stiff AR, et al. Gene expression profiling of the human natural killer cell response to Fc receptor activation: unique enhancement in the presence of interleukin-12. BMC Med Genomics. 2015;8:66.
38. Zhou R, Pei B, Li X, Zhang X. Involvement of S100A6/S100A11 in T-Cell Immune Regulatory in HCC Revealed by Single Cell RNA-seq. Technol Cancer Res Treat. 2024;23:15330338241252610.

39. Zeng Q, Li L, Feng Z, Luo L, Xiong J, Jie Z, et al. LCP1 is a prognostic biomarker correlated with immune infiltrates in gastric cancer. Cancer Biomark. 2021;30(1):105-25.
40. Lanfranca MP, Lazarus J, Shao X, Nathan H, Di Magliano MP, Zou W, et al. Tracking Macrophage Infiltration in a Mouse Model of Pancreatic Cancer with the Positron Emission Tomography Tracer [11C]PBR28. J Surg Res. 2018;232:570-7.
41. Zhu S, Zu L, Xu S. [The Expression of RTN1 in Lung Adenocarcinoma and Its Effect on Immune Microenvironment]. Zhongguo Fei Ai Za Zhi. 2022;25(6):385-95.
42. Sun PP, Liao SX, Sang P, Liu MM, Yang JB. Lysosomal transmembrane protein 5: Impact on immune cell function and implications for immune-related deficiencies. Heliyon. 2024;10(17):e36705.
43. Rashid NU, Peng XL, Jin C, Moffitt RA, Volmar KE, Belt BA, et al. Purity Independent Subtyping of Tumors (PurIST), A Clinically Robust, Single-sample Classifier for Tumor Subtyping in Pancreatic Cancer. Clin Cancer Res. 2020;26(1):82-92.
44. Andrade Barbosa B, van Asten SD, Oh JW, Farina-Sarasqueta A, Verheij J, Dijk F, et al. Bayesian log-normal deconvolution for enhanced in silico microdissection of bulk gene expression data. Nat Commun. 2021;12(1):6106.
45. Newman AM, Steen CB, Liu CL, Gentles AJ, Chaudhuri AA, Scherer F, et al. Determining cell type abundance and expression from bulk tissues with digital cytometry. Nat Biotechnol. 2019;37(7):773-82.
46. Chu T, Wang Z, Pe'er D, Danko CG. Cell type and gene expression deconvolution with BayesPrism enables Bayesian integrative analysis across bulk and single-cell RNA sequencing in oncology. Nat Cancer. 2022;3(4):505-17.
47. Chen Y, Wang Y, Chen Y, Cheng Y, Wei Y, Li Y, et al. Deep autoencoder for interpretable tissue-adaptive deconvolution and cell-type-specific gene analysis. Nat Commun. 2022;13(1):6735.
48. Paszke A, Gross S, Massa F, Lerer A, Bradbury J, Chanan G, et al. PyTorch: an imperative style, high-performance deep learning library. Proceedings of the 33rd International Conference on Neural Information Processing Systems: Curran Associates Inc.; 2019. p. Article 721.
49. Krebs S, Fischaleck M, Blum H. A simple and loss-free method to remove TRIzol contaminations from minute RNA samples. Anal Biochem. 2009;387(1):136-8.
