# Supplementary material for: Statescope: an integrative deconvolution framework for discovering cell states in tumors
Source: Nat Commun. 2026 Jul 6;17:8465. doi: 10.1038/s41467-026-74997-8 (PMC13478200; doi:10.1038/s41467-026-74997-8)
Supplement: Supplementary file 2 [file 41467_2026_74997_MOESM2_ESM.pdf]

nature portfolio

Corresponding author(s): Yongsoo Kim

Last updated by author(s): Jun 8, 2026

Reporting Summary

Nature Portfolio wishes to improve the reproducibility of the work that we publish. This form provides structure for consistency and transparency in reporting. For further information on Nature Portfolio policies, see our Editorial Policies and the Editorial Policy Checklist.

Statistics

For all statistical analyses, confirm that the following items are present in the figure legend, table legend, main text, or Methods section.

n/a	Confirmed	
☐	☒	The exact sample size (*n*) for each experimental group/condition, given as a discrete number and unit of measurement
☒	☐	A statement on whether measurements were taken from distinct samples or whether the same sample was measured repeatedly
☐	☒	The statistical test(s) used AND whether they are one- or two-sided<br>*Only common tests should be described solely by name; describe more complex techniques in the Methods section.*
☒	☐	A description of all covariates tested
☐	☒	A description of any assumptions or corrections, such as tests of normality and adjustment for multiple comparisons
☐	☒	A full description of the statistical parameters including central tendency (e.g. means) or other basic estimates (e.g. regression coefficient) AND variation (e.g. standard deviation) or associated estimates of uncertainty (e.g. confidence intervals)
☐	☒	For null hypothesis testing, the test statistic (e.g. *F*, *t*, *r*) with confidence intervals, effect sizes, degrees of freedom and *P* value noted<br>*Give P values as exact values whenever suitable.*
☐	☒	For Bayesian analysis, information on the choice of priors and Markov chain Monte Carlo settings
☒	☐	For hierarchical and complex designs, identification of the appropriate level for tests and full reporting of outcomes
☐	☒	Estimates of effect sizes (e.g. Cohen's *d*, Pearson's *r*), indicating how they were calculated

Our web collection on statistics for biologists contains articles on many of the points above.

Software and code

Policy information about availability of computer code

Data collection	No specific software was used for data collection
Data analysis	Statescope software and documentation is available on Github at https://github.com/tgac-vumc/Statescope/. Further documentation can be found at https://tgac-vumc.github.io/Statescopeweb/. Scripts to run ACE to copy number segments of the TCGA lung cohort can be found at https://github.com/tgac-vumc/TCGA_purity. BayesPrism (R-package (2.0)), CIBERSORTx (Docker version (1.0)) and TAPE (python package (vl.1.2)) were used according to documentation with default settings. scRNAseq processing was done using Scanpy (python package (vl.7.0)). Other scRNAseq signature creation steps were done with scran (v.1.34.0) and AutoGeneS (v.1.0.4)

For manuscripts utilizing custom algorithms or software that are central to the research but not yet described in published literature, software must be made available to editors and reviewers. We strongly encourage code deposition in a community repository (e.g. GitHub). See the Nature Portfolio guidelines for submitting code & software for further information.

Data

Policy information about availability of data

All manuscripts must include a data availability statement. This statement should provide the following information, where applicable:

- Accession codes, unique identifiers, or web links for publicly available datasets
- A description of any restrictions on data availability
- For clinical datasets or third party data, please ensure that the statement adheres to our policy

An complete overview of datasets and accession codes are available in Supplementary Table 1-2. Processed scRNA-seq datasets are publicly available: Stephenson et al (24) (E-MTAB-10026) , Salcher et al (3) (https://zenodo.org/records/7227571) and Werba et al (35) (GSE205013). Raw and processed bulk RNA-seq data from the PBMC samples with matched CyTOF are deposited in the NCBI Gene Expression Omnibus (GEO) under accession code GSE263756. Bulk RNA sequencing data from Hoek et al are deposited on GEO under accession code GSE64655. Access to bulk RNA and clinical data from the HMF cohort can be requested through https://www.hartwigmedicalfoundation.nl/en/data/data-access-request/ and is granted upon evaluation of the HMF Data Access Board. Access to the bulk RNA and clinical data from the POPLOAR/OAK cohort can be requested through European Genome-Phenome Archive database under accession code EGAD00001007703. TCGA copy number aberration-, RNA- and clinical data were downloaded from the GDC data portal (GDC). The TCGA cell state atlas results can be interacted with and found on the R2 platform (http://r2platform.com/statescope/).

Research involving human participants, their data, or biological material

Policy information about studies with human participants or human data. See also policy information about sex, gender (identity/presentation), and sexual orientation and race, ethnicity and racism.

Reporting on sex and gender	We did not collect any patient information including sex/gender on human participants donated their blood for our PBMC dataset, as they are used for only benchmarking purposes.
Reporting on race, ethnicity, or other socially relevant groupings	We did not collect any patient information including race/ethnicity on human participants donated their blood for our PBMC dataset, as they are used for only benchmarking purposes.
Population characteristics	We did not collect any patient information including age or any specific population characteristics on human participants donated their blood for our PBMC dataset, as they are used for only benchmarking purposes.
Recruitment	The patient recruitment was done prior to this study based on a liquid biopsy biobanking purpose.
Ethics oversight	Yes, the collection and analysis of the materials are done under the relevant METC approval. See Ethics statement.

Note that full information on the approval of the study protocol must also be provided in the manuscript.

Field-specific reporting

Please select the one below that is the best fit for your research. If you are not sure, read the appropriate sections before making your selection.

☒ Life sciences ☐ Behavioural & social sciences ☐ Ecological, evolutionary & environmental sciences

For a reference copy of the document with all sections, see nature.com/documents/nr-reporting-summary-flat.pdf

Life sciences study design

All studies must disclose on these points even when the disclosure is negative.

Sample size	A full overview of samples included in this study can be found in Supplementary Table 1<br><br>We included PBMC samples of 48 patients for which material was available.<br>We included 8 PBMC samples from Hoek et al.<br><br>We included public NSCLC scRNAseq data of 254 samples for pseudobulk generation to allow for sufficient size. We included 973 TCGA-NSCLC, 119 HMF and 891 POPLAR/OAK samples<br><br>We included public PDAC scRNAseq data from 27 samples.<br>We included 166 TCGA-PDAC samples
Data exclusions	Two PBMC samples with ~16% and ~so% of undefined fractions in CyTOF were excluded from analysis since they were not useable for evaluation.<br>We excluded TCGA samples with an ACE estimated purity of 100% before selection because they had no detectable copy number alterations.
Replication	We describe all preprocessing and analysis steps in the methods section. Identical UMAP visualizations as in the manuscript can not be reproduced.

Randomization	No randomization was performed
Blinding	For simullation experiments, different independent datasets were used for pseudobulk generation and signature creation

Reporting for specific materials, systems and methods

We require information from authors about some types of materials, experimental systems and methods used in many studies. Here, indicate whether each material, system or method listed is relevant to your study. If you are not sure if a list item applies to your research, read the appropriate section before selecting a response.

Materials & experimental systems

n/a	Involved in the study
☒	☐ Antibodies
☒	☐ Eukaryotic cell lines
☒	☐ Palaeontology and archaeology
☒	☐ Animals and other organisms
☒	☐ Clinical data
☒	☐ Dual use research of concern
☒	☐ Plants

Methods

n/a	Involved in the study
☒	☐ ChIP-seq
☒	☐ Flow cytometry
☒	☐ MRI-based neuroimaging

Plants

Seed stocks	*Report on the source of all seed stocks or other plant material used. If applicable, state the seed stock centre and catalogue number. If plant specimens were collected from the field, describe the collection location, date and sampling procedures.*
Novel plant genotypes	*Describe the methods by which all novel plant genotypes were produced. This includes those generated by transgenic approaches, gene editing, chemical/radiation-based mutagenesis and hybridization. For transgenic lines, describe the transformation method, the number of independent lines analyzed and the generation upon which experiments were performed. For gene-edited lines, describe the editor used, the endogenous sequence targeted for editing, the targeting guide RNA sequence (if applicable) and how the editor was applied.*
Authentication	*Describe any authentication procedures for each seed stock used or novel genotype generated. Describe any experiments used to assess the effect of a mutation and, where applicable, how potential secondary effects (e.g. second site T-DNA insertions, mosiacism, off-target gene editing) were examined.*
