# Supplementary material for: Statescope: an integrative deconvolution framework for discovering cell states in tumors
Source: Nat Commun. 2026 Jul 6;17:8465. doi: 10.1038/s41467-026-74997-8 (PMC13478200; doi:10.1038/s41467-026-74997-8)
Supplement: Supplementary file 3 [file 41467_2026_74997_MOESM3_ESM.pdf]

nature portfolio

Peer Review File

Statescope: An integrative deconvolution framework for discovering cell states in tumors

Corresponding Author: Dr Yongsoo Kim

This manuscript has been previously reviewed at another journal. This document only contains information relating to versions considered at Nature Communications.

This file contains all reviewer reports in order by version, followed by all author rebuttals in order by version.

Version 0:

Reviewer comments:

Reviewer #4

(Remarks to the Author)
This manuscript presents Statescope, a bulk-RNA–based deconvolution and cell-state discovery algorithm. The authors find that incorporating external information, such as malignant-cell fraction estimates, typically obtained from other modalities such as DNA copy-number, enables more accurate estimation of cell-type–specific expression profiles and identifies cellular states from bulk tissue.

In the last round of review, although both reviewers found the idea of including information such as malignant-cell fractions valuable, they raised several major questions. Reviewer 1 raised question on the novelty of the method over the previous BLADE method, as well as the robustness to cell-type annotation. Reviewer 2 was mainly concerned about 1) the assumption of a single malignant population 2) insufficient benchmarking against widely used tools, 3) narrow TCGA analysis and 4) robustness to noisy or biased.

In response, the authors in the revision 1) reframed the work and renamed the method Statescope to discovering cell states from bulk transcriptomes, adding a cNMF-based module 2) replaced subjective annotations with curated scRNA-seq atlas 3) added new benchmarking showing improved reconstruction of cell type–specific expression 4) expanded to the full TCGA cohort 5) added robustness experiments to test sensitivity of T-cell fraction priors on PBMC dataset.

Overall, I find the revised manuscript is clearly written and substantially improved, especially on the expanded biological analysis and more rigorous cell annotations. In the meantime, I still have several questions for authors to improve the manuscript on technical soundness.
1) To ensure robustness and reproducibility of the algorithm, please disclose all tunable hyper-parameters in the algorithm, identify key hyperparameters with sensivity analysis (especially those in the Bayesian model and cNMF), and provide rationale to select default. Especially, I found that in real data analysis, number of factors k in c-NMF is manually selected, its robustness is not reported.
2) The benchmarking is informative but still seems not entirely fair. I agree with Reviewer 2’s previous comments that, Statescope is the only method that receives external purity priors, yet its performance is compared directly to tools that cannot use such information. From a methodological standpoint, this makes it difficult to disentangle genuine algorithmic advantages from the simple benefit of having more input information. A cleaner evaluation would require an ablation-style design, e.g. systematically withholding/gradually increasing purity priors from Statescope to measure its baseline performance, or injecting analogous priors into other methods when possible (e.g., fixing or regularizing malignant fractions in BayesPrism, CIBERSORTx TAPE).
3) Relevant to the previous point, the robustness experiment conducted on PBMCs, where noise is injected into T-cell fractions, seems not reflect the types of bias that are more typical in tumor purity estimation. A more controlled and tumor-relevant robustness analysis would strengthen confidence. For instance, how the error in malignant cell fraction estimation using copy number by ACE affects the accuracy author’s algorithm?

(Remarks on code availability)
I have reviewed the code for its reproducibility. However, to maximize the impact and ensure wide usage by the community, I suggest the authors to provide a more thorough tutotial and a dedicated webpage for Statescope in the revisoin.

Reviewer #5

(Remarks to the Author)
Statescope provides a robust framework for discovering transcriptional states from bulk RNA-seq data. The pipeline ran successfully on both the provided PBMC dataset and an independent TCGA pancreatic cancer dataset. However, installation and execution require careful environment configuration, and some dependencies are sensitive to Python and package versions, please see below for the details. The authors need to make sure others can easily run their codes in various computing environments.

Also, the resolution of the figures (graphical abstract and main figures) is very low. While it was barely possible to see the data in the figures, the authors should improve their resolution in the revised version.

Installation errors:

Tried to install using python version 3.10.19
Collecting scipy (from Statescope)
Using cached scipy-1.3.0.tar.gz (23.6 MB)
Installing build dependencies ... error
error: subprocess-exited-with-error

× installing build dependencies for scipy did not run successfully.
│ exit code: 1
╰─> [59 lines of output]
Ignoring numpy: markers 'python_version == "3.5"' don't match your environment
Ignoring numpy: markers 'python_version == "3.6"' don't match your environment
Collecting wheel
Using cached wheel-0.46.3-py3-none-any.whl.metadata (2.4 kB)
Collecting setuptools
Using cached setuptools-80.10.2-py3-none-any.whl.metadata (6.6 kB)
Collecting Cython>=0.29.2
Using cached cython-3.2.4-cp310-cp310-
manylinux2014_x86_64.manylinux_2_17_x86_64.manylinux_2_28_x86_64.whl.metadata (7.5 kB)
Collecting numpy==1.14.5
Using cached numpy-1.14.5.zip (4.9 MB)
Installing build dependencies: started
Installing build dependencies: finished with status 'done'
Getting requirements to build wheel: started
Getting requirements to build wheel: finished with status 'done'
Preparing metadata (pyproject.toml): started
Preparing metadata (pyproject.toml): finished with status 'error'
error: subprocess-exited-with-error
× Preparing metadata (pyproject.toml) did not run successfully.
│ exit code: 1
╰─> [28 lines of output]
Running from numpy source directory.
<string>:369: UserWarning: Unrecognized setuptools command, proceeding with generating Cython sources and expanding templates
/tmp/pip-install-2wz8_dff/numpy_4726c3467c374f5ca081b1021d018be3/numpy/distutils/misc_util.py:464: SyntaxWarning: "is" with a literal. Did you mean "=="?
return is_string(s) and ('*' in s or '?' is s)
Traceback (most recent call last):
File "/home/umar/miniconda3/envs/statescope/lib/python3.10/site-
packages/pip/_vendor/pyproject_hooks/_in_process/_in_process.py", line 389, in <module>
main()
File "/home/umar/miniconda3/envs/statescope/lib/python3.10/site-
packages/pip/_vendor/pyproject_hooks/_in_process/_in_process.py", line 373, in main
json_out["return_val"] = hook(**hook_input["kwargs"])
File "/home/umar/miniconda3/envs/statescope/lib/python3.10/site-
packages/pip/_vendor/pyproject_hooks/_in_process/_in_process.py", line 175, in prepare_metadata_for_build_wheel
return hook(metadata_directory, config_settings)
File "/tmp/pip-build-env-cwgc4e3w/overlay/lib/python3.10/site-packages/setuptools/build_meta.py", line 378, in
prepare_metadata_for_build_wheel
self.run_setup()
File "/tmp/pip-build-env-cwgc4e3w/overlay/lib/python3.10/site-packages/setuptools/build_meta.py", line 518, in run_setup
super().run_setup(setup_script=setup_script)
File "/tmp/pip-build-env-cwgc4e3w/overlay/lib/python3.10/site-packages/setuptools/build_meta.py", line 317, in run_setup
exc(code, locals())

File "<string>", line 394, in <module>
File "<string>", line 373, in setup_package
File "/tmp/pip-install-2wz8_dff/numpy_4726c3467c374f5ca081b1021d018be3/numpy/distutils/__init__.py", line 8, in <module>
from . import ccompiler
File "/tmp/pip-install-2wz8_dff/numpy_4726c3467c374f5ca081b1021d018be3/numpy/distutils/ccompiler.py", line 17, in <module>
from numpy.distutils import log
File "/tmp/pip-install-2wz8_dff/numpy_4726c3467c374f5ca081b1021d018be3/numpy/distutils/log.py", line 13, in <module>
from numpy.distutils.misc_util import (red_text, default_text, cyan_text,
File "/tmp/pip-install-2wz8_dff/numpy_4726c3467c374f5ca081b1021d018be3/numpy/distutils/misc_util.py", line 15, in <module>
from distutils.msvccompiler import get_build_architecture
ModuleNotFoundError: No module named 'distutils.msvccompiler'
[end of output]

note: This error originates from a subprocess, and is likely not a problem with pip.
error: metadata-generation-failed

× Encountered error while generating package metadata.
╰─> numpy

note: This is an issue with the package mentioned above, not pip.
hint: See above for details.
[end of output]

note: This error originates from a subprocess, and is likely not a problem with pip.
ERROR: Failed to build 'scipy' when installing build dependencies for scipy

Solution

Executed the following commands to install stethoscope
• conda install -c conda-forge numpy scipy pandas scikit-learn
• pip install Statescope --no-deps

After this installed dependencies separately
#!pip install anndata
#!pip install statsmodels
#!pip install git+https://github.com/jurriant/autogenes.git
#!pip install dist/autogenes-1.0.4-py3-none-any.whl
#!pip install autogenes
#!pip install matplotlib
#!pip install deap
#!pip install cachetools
#!!pip install dill
#!pip install torchvision
#!pip install numba
!pip install seaborn

To install the autogenes executed the following commands:
git clone https://github.com/theislab/AutoGeneS
cd AutoGeneS
pip install dist/autogenes-1.0.4-py3-none-any.whl --no-deps

The authors should minimize the dependencies, or at least describe clearly in a readme file which Python and package versions should be used.

(Remarks on code availability)
Installation and execution require careful environment configuration, and some dependencies are sensitive to Python and package versions. I have listed these issues in the author comments box.

Version 1:

Reviewer comments:

Reviewer #4

(Remarks to the Author)

The authors have fully addressed my questions, and I have no further comments. I recommend the acceptance of the manuscript.

(Remarks on code availability)

Reviewer #5

(Remarks to the Author)
The authors have addressed my comments. Having a stable bioconductor package is great for a long-term sustainability of the tool.

(Remarks on code availability)

Open Access This Peer Review File is licensed under a Creative Commons Attribution 4.0 International License, which permits use, sharing, adaptation, distribution and reproduction in any medium or format, as long as you give appropriate credit to the original author(s) and the source, provide a link to the Creative Commons license, and indicate if changes were made.
In cases where reviewers are anonymous, credit should be given to 'Anonymous Referee' and the source.
The images or other third party material in this Peer Review File are included in the article's Creative Commons license, unless indicated otherwise in a credit line to the material. If material is not included in the article's Creative Commons license and your intended use is not permitted by statutory regulation or exceeds the permitted use, you will need to obtain permission directly from the copyright holder.
To view a copy of this license, visit https://creativecommons.org/licenses/by/4.0/

- *Black fonts = copy & paste of reviewer comments*
- *Blue fonts = author response to the comments*
- *Red fonts = relevant text with modification in the manuscript*

REVIEWER COMMENTS

Reviewer #4 (Remarks to the Author): Expert in single-cell RNA-seq, bioinformatics, machine learning, computational systems biology; replaces Reviewer #1 and Reviewer #2

This manuscript presents Statescope, a bulk-RNA–based deconvolution and cell-state discovery algorithm. The authors find that incorporating external information, such as malignant-cell fraction estimates, typically obtained from other modalities such as DNA copy-number, enables more accurate estimation of cell-type–specific expression profiles and identifies cellular states from bulk tissue.

In the last round of review, although both reviewers found the idea of including information such as malignant-cell fractions valuable, they raised several major questions. Reviewer 1 raised question on the novelty of the method over the previous BLADE method, as well as the robustness to cell-type annotation. Reviewer 2 was mainly concerned about 1) the assumption of a single malignant population 2) insufficient benchmarking against widely used tools, 3) narrow TCGA analysis and 4) robustness to noisy or biased.

In response, the authors in the revision 1) reframed the work and renamed the method Statescope to discovering cell states from bulk transcriptomes, adding a cNMF-based module 2) replaced subjective annotations with curated scRNA-seq atlas 3) added new benchmarking showing improved reconstruction of cell type–specific expression 4) expanded to the full TCGA cohort 5) added robustness experiments to test sensitivity of T-cell fraction priors on PBMC dataset.

Overall, I find the revised manuscript is clearly written and substantially improved, especially on the expanded biological analysis and more rigorous cell annotations. In the meantime, I still have several questions for authors to improve the manuscript on technical soundness.
1) To ensure robustness and reproducibility of the algorithm, please disclose all tunable hyper-parameters in the algorithm, identify key hyperparameters with sensivity analysis (especially those in the Bayesian model and cNMF), and provide rationale to select default. Especially, I found that in real data analysis, number of factors k in c-NMF is manually selected, its robustness is not reported.

We appreciate the reviewer’s comment to provide more rationale for the hyperparameter selection. For the Bayesian modelling we have hyperparameters for which we provide a default configuration, unlike the empirical Bayes framework used in predecessor, BLADE.

The rationale for the hyperparameters is to make them uninformative, as we do not have a good prior knowledge. The exception is α_{0j}^{t}=1000, which indicates a high confidence in the observed variability in single-cell gene expression data. These default configurations enable us to omit time-consuming hyperparameter tuning steps.

We provided the rationale to this in the method section as below:

In all Statescope applications, the default hyperparameter configurations are applied: α_{i}^{t}=1, α_{0j}^{t}=1000, κ_{0j}^{t}=1, s=1 and 100 optimization iterations. These hyperparameters are uninformative priors, except for the α_{0j}^{t}=1000 that puts a high confidence on observed gene expression variability in single-cell RNAseq data. These default configurations allow us to omit the time-consuming hyperparameter optimization based on the previous Empirical Bayes framework.

In the refinement step, we modified the objective function by down-weighting the second term $E_Q[log\ P(X|\theta)\ ]$ by a factor of 100. The rationale for this scaling factor is driven by the structural dimensionality of the model. In our empirical evaluations (e.g., lung and pancreatic cancer applications), we observed that the second term is naturally 15-20 times larger than the first term $E_Q[log\ P(Y|X,F,\sigma)\ ]$. This magnitude difference occurs because the second term evaluates the likelihood across a much larger parameter space (Gene x Sample x Cell Type) compared to the first term (Gene x Sample). In the initial deconvolution step, this dominant second term is highly beneficial as it strictly enforces the single-cell prior knowledge. However, the goal of the refinement step is to capture tumor-specific variations not present in the reference. By down-weighting the second term by a factor of 100, the first term becomes roughly 5-6 times larger than the second term. This effectively shifts the optimization focus, relaxing the strict adherence to the prior and allowing the objective function to prioritize absorbing the residuals in the bulk gene expression data.

We provided this rationale in the method section as below:

To address interpatient heterogeneity during the refinement step, we relax the model's reliance on prior cell-type-specific gene expression profiles by down-weighting the second term of the objective function, $E_Q[log\ P(X|\theta)\ ]$, by a factor of 100. This scaling factor is derived from the empirical observation that the second term is typically 15–20 times larger in magnitude compared to the first term. This size difference occurs because the second term evaluates a much larger parameter space (Genes × Samples × Cell Types) compared to the first term (Genes × Samples). During the initial deconvolution, this magnitude difference is advantageous as it strictly enforces adherence to the single-cell prior knowledge. However, to capture patient-specific tumor variations, the model must be allowed flexibility. By applying the $\frac{1}{100}$ scaling factor to the first term (representing bulk data reconstruction) becomes roughly 5–6 times larger than the second term. This shift in the

optimization landscape allows the estimated x_{ij}^{t} to deviate from the reference single-cell profiles, absorbing sample-specific residuals in the bulk gene expression data and thereby achieving a more accurate, patient-specific reconstruction.

For state discovery using cNMF, we adopted the same strategy to the EcoTyper framework (Luca et al., 2021), except for a cophenetic coefficient threshold of 0.9 instead of 0.95. We agree with the reviewer that selecting the optimal number of factors (k) is a critical parameter. Because this is a classic model selection problem lacking a single universal consensus metric, we employed a highly systematic, multi-step approach.

First, we evaluated a search space ranging from $k = 2$ up until $k = 9$. To quantitatively assess stability, we calculated the cophenetic correlation coefficients for each k, aiming for a stability threshold of approximately 0.90. Following the heuristic widely used in NMF applications (including EcoTyper), we selected the k located just before a significant drop in the cophenetic coefficient. In cases where multiple k values exhibited similar mathematical stability, we used biological interpretability as the decisive tie-breaker to ensure the resulting states were biologically meaningful rather than statistical artifacts.

To provide complete transparency regarding how these k values were chosen, we have added **Supplementary Figures 11 and 12**, which display the cophenetic coefficient plots across all cell types for both the NSCLC and PDAC TCGA state discovery. These figures are cited in the Methods section as below:

Based on the heuristic algorithm by Luca et al (10), the most stable value for k is selected as the optimal number of cell states. Here a minimal value of the cophenetic coefficient is used as a parameter to determine the stable clustering solutions (default=0.9). The dynamics of the cophenetic coefficients and our final choice of k for each cell type are reported in Supplementary Figures 11 and 12 for the NSCLC and the PDAC applications respectively.

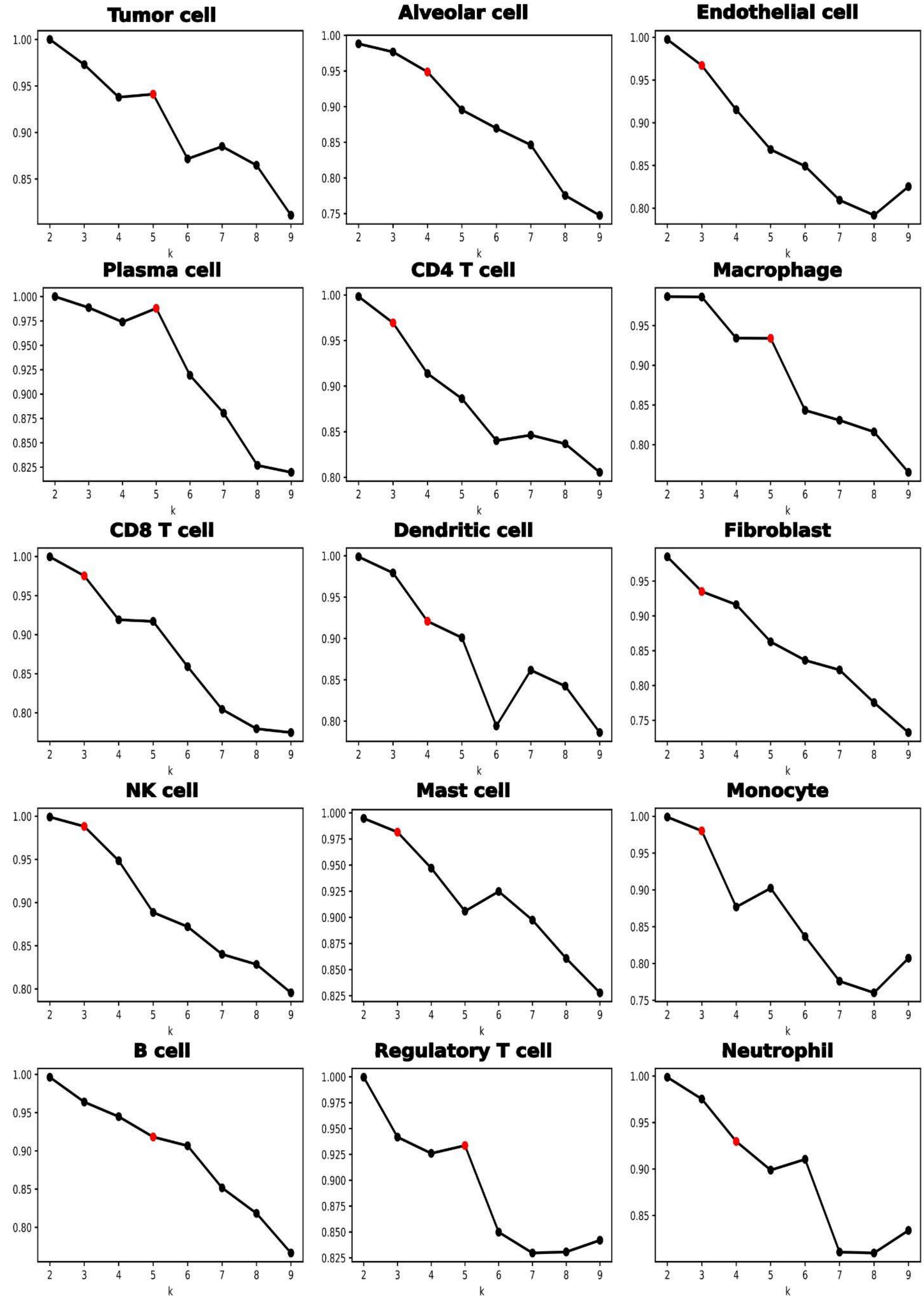

Supplementary Figure 11. Cophenetic coefficient plots for the NSCLC TCGA State Discovery.
Cophenetic coefficients of the initial cNMF runs (10 random initializations) for k's ranging from 2-9 for all cell types. The red dots represent the k we chose for the final cNMF run (100 random initializations).

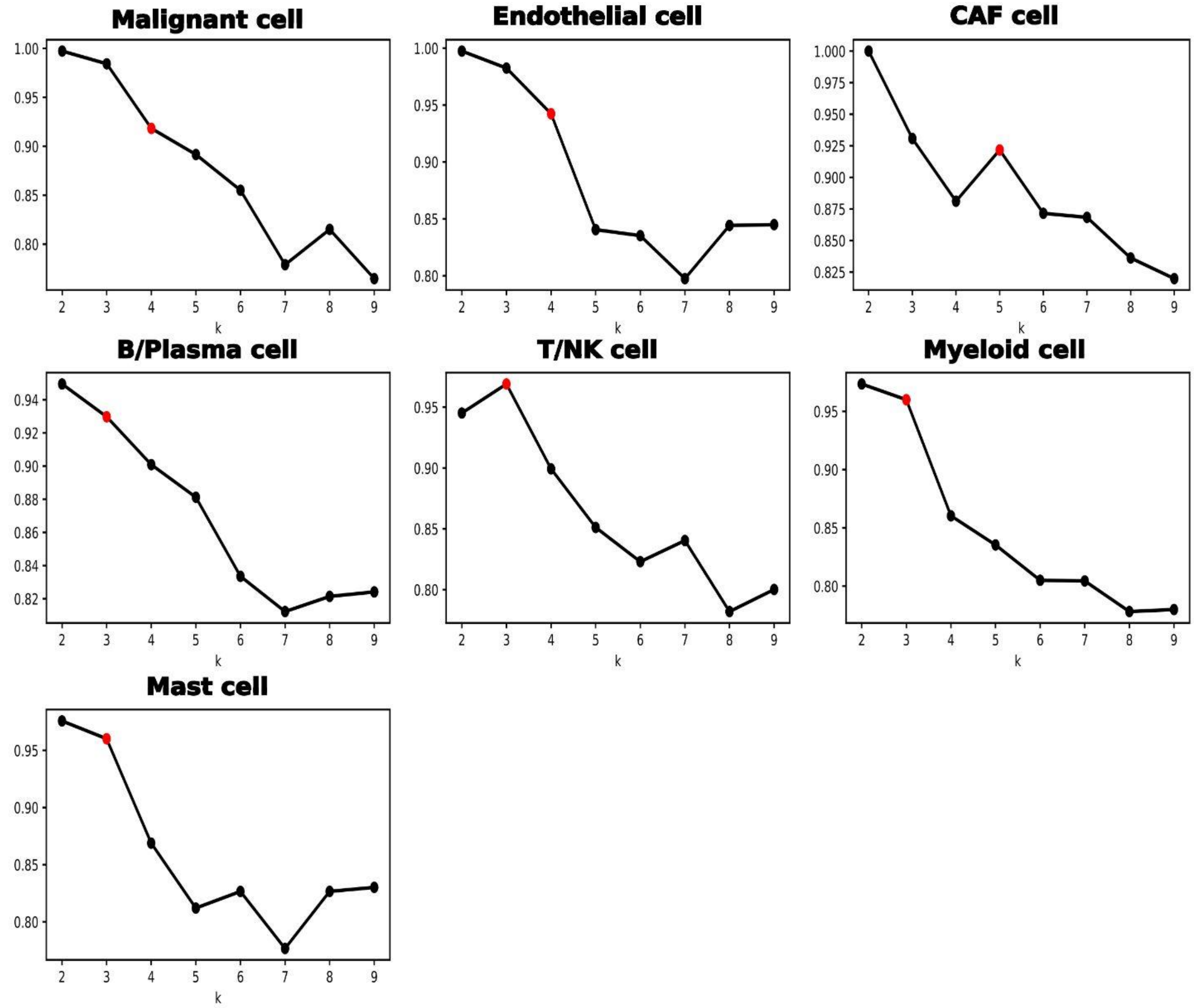

Supplementary Figure 12. Cophenetic coefficient plots for the PDAC TCGA State Discovery.
Cophenetic coefficients of the initial cNMF runs (10 random initializations) for k's ranging from 2-9 for all cell types. The red dots represent the k we chose for the final cNMF run (100 random initializations).

2) The benchmarking is informative but still seems not entirely fair. I agree with Reviewer 2's previous comments that, Statescope is the only method that receives external purity priors, yet its performance is compared directly to tools that cannot use such information. From a methodological standpoint, this makes it difficult to disentangle genuine algorithmic advantages from the simple benefit of having more input information. A cleaner evaluation would require an ablation-style design, e.g. systematically withholding/gradually increasing purity priors from Statescope to measure its baseline performance, or injecting analogous priors into other methods when possible (e.g., fixing or regularizing malignant fractions in BayesPrism, CIBERSORTx TAPE).

We thank the reviewer for raising this important point and agree that disentangling the genuine algorithmic advantages from the benefit of additional prior information is a crucial point.

First, we emphasize that the unique ability to seamlessly integrate external, DNA-derived tumor purity priors is a core architectural feature and major methodological contribution of Statescope. While we agree that injecting analogous priors into other methods would be an interesting theoretical exercise, modifying the underlying statistical frameworks and softwares of BayesPrism or CIBERSORTx to accept these external priors is technically non-trivial and falls outside the scope of this study.

However, we fully agree with the reviewer's suggestion to perform an ablation-style evaluation to establish Statescope's baseline performance. To this end, we have performed an additional benchmarking evaluation of Statescope *without* providing the external purity priors **(Supplementary Figure 3; see below)**. In this task evaluating the reconstruction of T cell gene expression profiles, Statescope without prior knowledge still significantly outperformed all other baseline methods. Furthermore, as expected, Statescope's performance improved even further when the external prior knowledge was reintroduced. This ablation study clearly demonstrates that Statescope possesses a distinct underlying algorithmic advantage over existing methods, which is then synergistically enhanced by its unique ability to leverage external purity data.

Accordingly, we provide this discussion in the result section as below:

In PBMC T cell gene expression profiling using the gold-standard dataset 2, Statescope achieved the highest concordance between predicted T cell profiles and FACS-sorted T cell profiles (PCC= 0.78 (range: 0.77-0.79)), significantly outperforming BayesPrism (PCC= 0.57 (range:0.56-0.58); one-sided Wilcoxon p-value=0.003906) and TAPE (PCC= 0.45 (range 0.41-0.49); one-sided Wilcoxon p-value=0.003906; Figure 1i). Without prior T cell fraction information, Statescope also outperformed both methods (PCC= 0.73 (range: 0.72-0.74); one-sided Wilcoxon p-value= 0.0078). However, the addition of this prior information caused a significant improvement in performance (one-sided Wilcoxon p-value= 0.0078; Supplementary Figure 3).

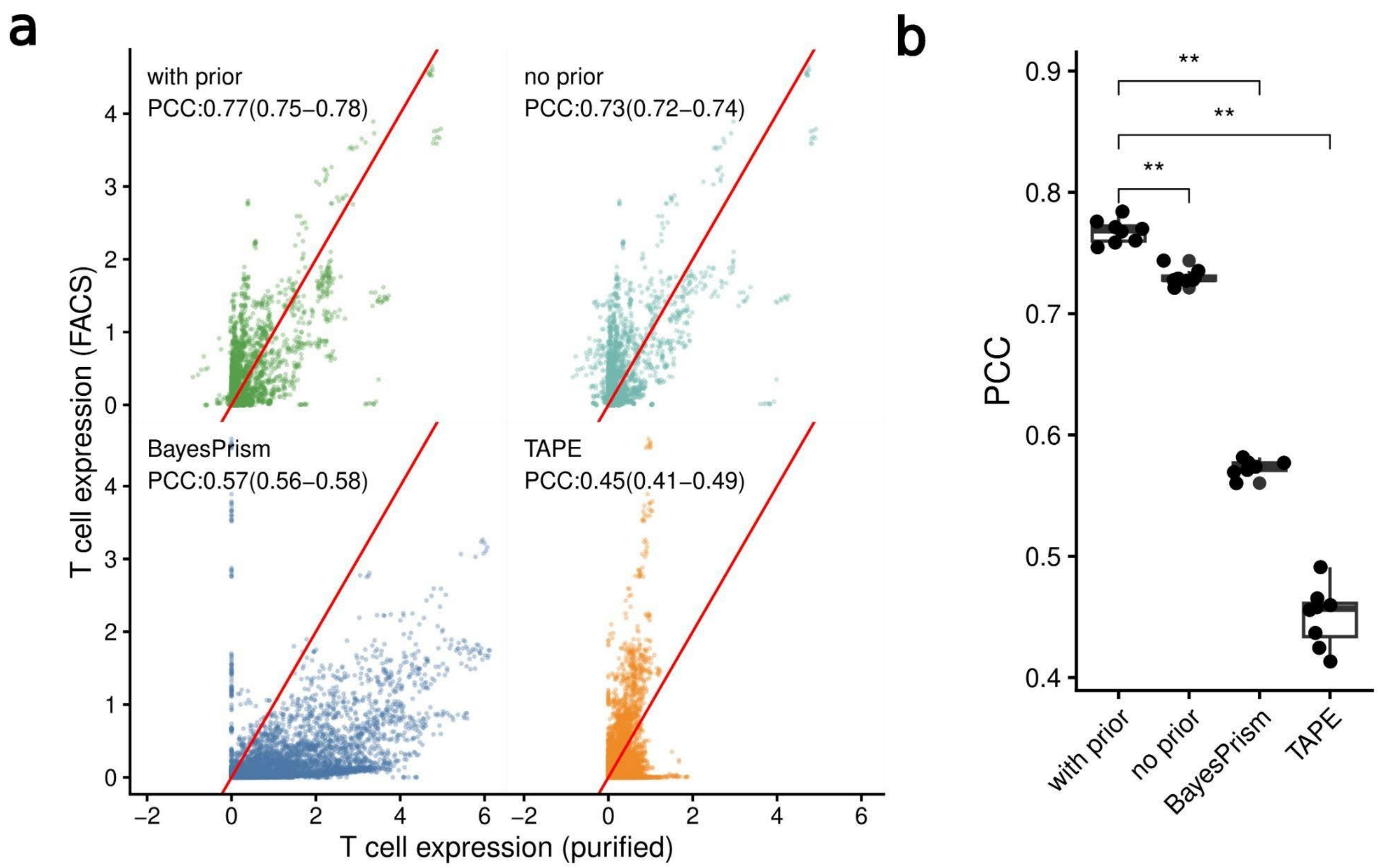

Supplementary Figure 3. Impact of T cell fraction prior on T cell gene expression profiles. Performance of cell type-specific gene expression estimation measured by PCC using predicted-T cell profiles and FACS-sorted T cell profiles in the gold-standard dataset 2 across 8 samples (**a**). Statescope performance is shown with T cell fraction prior (with prior) or without T cell fractions prior (no prior). PCC measuring T cell gene expression estimation performance per sample compared between the 3 tools, splitting Statescope into with T cell fraction prior and no T cell fraction prior (b).CIBERSORTx could not be applied in these evaluations due to limited sample size. Statistical significance in boxplot comparisons between Statescope with prior (with prior), Statescope with no prior (no prior) and other methods is determined using a one-sided, paired Wilcoxon rank-sum test and indicated as follows: **: p<=0.01

3) Relevant to the previous point, the robustness experiment conducted on PBMCs, where noise is injected into T-cell fractions, seems not reflect the types of bias that are more typical in tumor purity estimation. A more controlled and tumor-relevant robustness analysis would strengthen confidence. For instance, how the error in malignant cell fraction estimation using copy number by ACE affects the accuracy author's algorithm?

We appreciate the reviewer's valuable insight regarding the translation of our PBMC robustness experiments to real-world tumor applications. To address how realistic errors in malignant cell fraction estimation affect our algorithm, we quantified the actual noise levels expected in empirical tumor purity estimates to determine how they align with our simulated noise settings (low, intermediate, high).

To do this, we compared tumor purity estimates derived from two independent, orthogonal DNA-based methodologies: ACE (based on copy number profiles) and ABSOLUTE (based on variant allele frequencies). We observed a very strong concordance between these two independent estimates. Crucially, the variance between these two methods was actually lower than the variance injected into the "low noise" setting of our PBMC T-cell benchmark.

This observation reinforces two key points: first, that DNA-based tumor fraction estimation is highly robust and reliable in practice; and second, that our low noise setting of T cell fraction prior is actually a conservative representation of real-world tumor purity errors. Furthermore, as we demonstrate in **Supplementary Figure 2**, Statescope's Bayesian updating ensures that the posterior fraction estimation consistently improves upon the prior, successfully absorbing these minor estimation errors. We have now explicitly connected these findings in the main text in Result section as below:

Statescope was applied to characterize interpatient cell state heterogeneity for NSCLC using the The Cancer Genome Atlas (TCGA) dataset (N=973) (26, 27) (Figure 3a, Supplementary Table 1). First, we constructed prior knowledge of scRNA-seq signatures from the NSCLC scRNA-seq atlas by Salcher et al (3) (Supplementary Table 2) and predetermined malignant fractions for each of the TCGA samples from the DNA copy number aberrations with ACE (28) (see Methods for details). We confirmed that the ACE tumor fraction estimates were highly concordant with orthogonal, variant allele frequency-based estimates derived using ABSOLUTE (43) (Supplementary Note 6). Notably, the empirical deviation between these two methods was smaller than the 'low-noise' condition modeled in our PBMC T-cell benchmarking, confirming the high reliability of the DNA-derived priors and contextualizing the robustness of Statescope in real-world tumor applications.

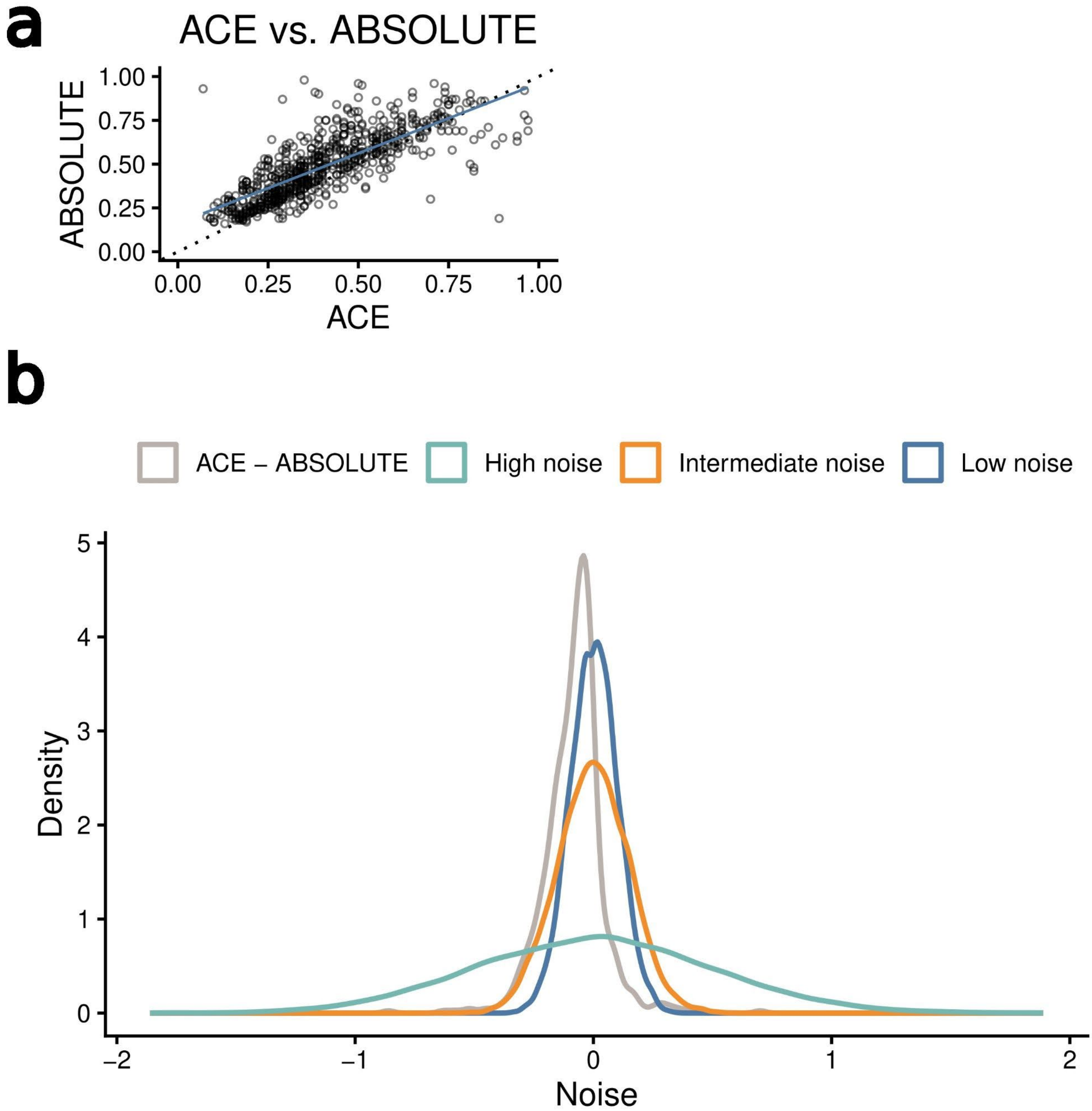

SN6 Figure 1: Comparison of different Gaussian noise levels with a more realistic estimation tool-based noise. Estimated tumor fractions by ABSOLUTE against estimated tumor fractions by ACE (a). Density plot comparing the low, intermediate and high Gaussian noise levels with the difference between the ACE and ABSOLUTE tumor fraction estimations (b).

Reviewer #4 (Remarks on code availability):

I have reviewed the code for its reproducibility. However, to maximize the impact and ensure wide usage by the community, I suggest the authors to provide a more thorough tutotial and a dedicated webpage for Statescope in the revisoin.

We appreciate the suggestion of the reviewer to add a webpage for Statescope and a more thorough tutorial. In light of this we have updated our webpage (Statescope | Statescope) and added our advanced tutorial to the tutorial section of this webpage. By following this tutorial, users can reproduce some of the key figures in the manuscript (Figure 4e and part of 4g, Supplementary Figure 12). Users can also find this tutorial straight from the Github page as well (https://github.com/tgac-vumc/Statescope). Finally, to further broaden accessibility and ensure wide adoption by the bioinformatics community, we have developed and officially published a Bioconductor R package (https://doi.org/doi:10.18129/B9.bioc.StatescopeR), which is accompanied by its own comprehensive vignette and basic tutorial on our new webpage.

Reviewer #5 (Remarks to the Author): Expert in bulk and single-cell RNA-seq, cancer multiomics, bioinformatics, machine learning, tumour microenvironment; replaces Reviewer #5

Statescope provides a robust framework for discovering transcriptional states from bulk RNA-seq data. The pipeline ran successfully on both the provided PBMC dataset and an independent TCGA pancreatic cancer dataset. However, installation and execution require careful environment configuration, and some dependencies are sensitive to Python and package versions, please see below for the details. The authors need to make sure others can easily run their codes in various computing environments.

We thank the reviewer for thoroughly testing our pipeline on multiple datasets and for bringing these environment configuration sensitivities to our attention.

Upon investigation, we traced the installation bottlenecks to an upstream dependency, the AutoGeneS package, which is incompatible with recent versions of pip (v24 and later). While this issue had been previously flagged in the AutoGeneS repository, it remained unresolved by the original authors. To provide a robust and permanent solution, we created a patched version of this dependency on PyPI (statescope-autogenes). We have updated the latest release of Statescope (v1.0.7) to depend on this patched version, fully resolving the pip installation conflicts.

Furthermore, to eliminate sensitivity to Python and package versions across different computing environments, we have added a strict conda environment definition file (environment.yml) to our GitHub repository. This explicitly pins all required package versions to guarantee highly reproducible installations.

We are confident these updates make the Python version of Statescope robust and easy to deploy. Lastly, to cater to a broader audience and leverage a highly stable dependency management ecosystem, we have also released Statescope as a Bioconductor R package (https://doi.org/10.18129/B9.bioc.StatescopeR), which strictly enforces fixed package versions to ensure long-term stability and reproducibility.

Also, the resolution of the figures (graphical abstract and main figures) is very low. While it was barely possible to see the data in the figures, the authors should improve their resolution in the revised version.
We appreciate the comments of the reviewer for making us aware of these issues. In this resubmitted version of the manuscript we have taken great care to ensure high resolution figures throughout the manuscript.

Installation errors:

Tried to install using python version 3.10.19
Collecting scipy (from Statescope)
Using cached scipy-1.3.0.tar.gz (23.6 MB)
Installing build dependencies ... error
error: subprocess-exited-with-error

× installing build dependencies for scipy did not run successfully.
│ exit code: 1
╰─> [59 lines of output]
Ignoring numpy: markers 'python_version == "3.5"' don't match your environment
Ignoring numpy: markers 'python_version == "3.6"' don't match your environment
Collecting wheel
Using cached wheel-0.46.3-py3-none-any.whl.metadata (2.4 kB)
Collecting setuptools
Using cached setuptools-80.10.2-py3-none-any.whl.metadata (6.6 kB)
Collecting Cython>=0.29.2
Using cached cython-3.2.4-cp310-cp310-
manylinux2014_x86_64.manylinux_2_17_x86_64.manylinux_2_28_x86_64.whl.metadata
(7.5 kB)
Collecting numpy==1.14.5
Using cached numpy-1.14.5.zip (4.9 MB)
Installing build dependencies: started
Installing build dependencies: finished with status 'done'
Getting requirements to build wheel: started
Getting requirements to build wheel: finished with status 'done'
Preparing metadata (pyproject.toml): started
Preparing metadata (pyproject.toml): finished with status 'error'
error: subprocess-exited-with-error
× Preparing metadata (pyproject.toml) did not run successfully.
│ exit code: 1
╰─> [28 lines of output]
Running from numpy source directory.
<string>:369: UserWarning: Unrecognized setuptools command, proceeding with
generating Cython sources and expanding templates
/tmp/pip-install-
2wz8_dff/numpy_4726c3467c374f5ca081b1021d018be3/numpy/distutils/misc_util.py:
464: SyntaxWarning: "is" with a literal. Did you mean "=="?
return is_string(s) and ('*' in s or '?' is s)
Traceback (most recent call last):

File "/home/umar/miniconda3/envs/statescope/lib/python3.10/site-packages/pip/_vendor/pyproject_hooks/_in_process/_in_process.py", line 389, in <module>
main()
File "/home/umar/miniconda3/envs/statescope/lib/python3.10/site-packages/pip/_vendor/pyproject_hooks/_in_process/_in_process.py", line 373, in main
json_out["return_val"] = hook(**hook_input["kwargs"])
File "/home/umar/miniconda3/envs/statescope/lib/python3.10/site-packages/pip/_vendor/pyproject_hooks/_in_process/_in_process.py", line 175, in prepare_metadata_for_build_wheel
return hook(metadata_directory, config_settings)
File "/tmp/pip-build-env-cwgc4e3w/overlay/lib/python3.10/site-packages/setuptools/build_meta.py", line 378, in prepare_metadata_for_build_wheel
self.run_setup()
File "/tmp/pip-build-env-cwgc4e3w/overlay/lib/python3.10/site-packages/setuptools/build_meta.py", line 518, in run_setup
super().run_setup(setup_script=setup_script)
File "/tmp/pip-build-env-cwgc4e3w/overlay/lib/python3.10/site-packages/setuptools/build_meta.py", line 317, in run_setup
exc(code, locals())
File "<string>", line 394, in <module>
File "<string>", line 373, in setup_package
File "/tmp/pip-install-2wz8_dff/numpy_4726c3467c374f5ca081b1021d018be3/numpy/distutils/__init__.py", line 8, in <module>
from . import ccompiler
File "/tmp/pip-install-2wz8_dff/numpy_4726c3467c374f5ca081b1021d018be3/numpy/distutils/ccompiler.py", line 17, in <module>
from numpy.distutils import log
File "/tmp/pip-install-2wz8_dff/numpy_4726c3467c374f5ca081b1021d018be3/numpy/distutils/log.py", line 13, in <module>
from numpy.distutils.misc_util import (red_text, default_text, cyan_text,
File "/tmp/pip-install-2wz8_dff/numpy_4726c3467c374f5ca081b1021d018be3/numpy/distutils/misc_util.py", line 15, in <module>
from distutils.msvccompiler import get_build_architecture
ModuleNotFoundError: No module named 'distutils.msvccompiler'
[end of output]

note: This error originates from a subprocess, and is likely not a problem with pip.
error: metadata-generation-failed

× Encountered error while generating package metadata.
╰─> numpy

note: This is an issue with the package mentioned above, not pip.
hint: See above for details.
[end of output]

note: This error originates from a subprocess, and is likely not a problem with pip.
ERROR: Failed to build 'scipy' when installing build dependencies for scipy

Solution

Executed the following commands to install stethoscope
• conda install -c conda-forge numpy scipy pandas scikit-learn
• pip install Statescope --no-deps

After this installed dependencies separately
#!pip install anndata
#!pip install statsmodels
#!pip install git+https://github.com/jurriant/autogenes.git
#!pip install dist/autogenes-1.0.4-py3-none-any.whl
#!pip install autogenes
#!pip install matplotlib
#!pip install deap
#!pip install cachetools
#!!pip install dill
#!pip install torchvision
#!pip install numba
!pip install seaborn

To install the autogenes executed the following commands:
git clone https://github.com/theislab/AutoGeneS
cd AutoGeneS
pip install dist/autogenes-1.0.4-py3-none-any.whl --no-deps

The authors should minimize the dependencies, or at least describe clearly in a readme file which Python and package versions should be used.

Reviewer #5 (Remarks on code availability):

Installation and execution require careful environment configuration, and some dependencies are sensitive to Python and package versions. I have listed these issues in the author comments box.

As answered above, we now provide a new version of the Python package (v1.0.7), which bypasses the dependency issue due to AutoGeneS. Furthermore, we provide the static conda environment on our Github page, which in principle should reproduce exact software versions for all dependencies used in the study.
